# Ubiquitin-specific protease 7 regulates macrophage polarization via pyruvate kinase M2-mediated metabolic reprogramming in severe acute pancreatitis

**DOI:** 10.1038/s41419-025-08081-2

**Published:** 2025-10-27

**Authors:** Yao Wu, Xudong Yao, Qiang Yu, Qingqing Yan, Xin Huang, Huajing Ke, Chao Peng, Liang Xia

**Affiliations:** 1 Department of Gastroenterology, Jiangxi Provincial Key Laboratory of Digestive Diseases, Jiangxi Clinical Research Center for Gastroenterology, Digestive Disease Hospital, The First Affiliated Hospital, Jiangxi Medical College, Nanchang University, Nanchang, Jiangxi Province PR China; 2 Gannan Medical University, School of the First Clinical Medicine, Ganzhou, Jiangxi Province PR China; 3 State Key Laboratory of Food Science and Resources, Nanchang University, Nanchang, Jiangxi Province PR China; 4 Department of Gastroenterology, Guangzhou First People’s Hospital, the Second Affiliated Hospital of South China University of Technology, Guangzhou, Guangdong Province PR China

**Keywords:** Acute inflammation, Cell division

## Abstract

Severe acute pancreatitis (SAP) is a life-threatening inflammatory condition, with macrophage polarization playing a pivotal role in its pathogenesis. This study aims to investigate the role and underlying mechanism of ubiquitin-specific protease 7 (USP7) in regulating macrophage polarization in SAP. The expression of USP7 in the pancreas of SAP mice were assessed to determine its involvement in the disease. Histological evaluation, immunofluorescence, flow cytometry, and Western blotting were utilized to phenotype macrophages and assess the expression of inflammatory markers in both mouse models and cell cultures. Seahorse assays were employed to measure the extracellular acidification rates (ECAR) and oxygen consumption rates (OCR). The interaction between USP7 and pyruvate kinase M2 (PKM2) was determined using co-immunoprecipitation (Co-IP) and ubiquitinated IP assays. Compound 3 K was administered to SAP mice for rescuing the impact of USP7 knockdown. USP7 expression was upregulated in pancreatic macrophages of mice with SAP, and its knockdown alleviated SAP, as evidenced by reduced serum amylase and lipase activities, as well as decreased expression of pro-inflammatory cytokines. USP7 knockdown also shifted macrophage polarization from M1 to M2 types, both in vivo and in vitro. Mechanistically, USP7 modulated the metabolic reprogramming of M1 macrophages by mediating PKM2 deubiquitination, which influenced its phosphorylation and nuclear translocation. Furthermore, a PKM2 inhibitor partially reversed the protective effects of USP7 knockdown in SAP mice, confirming that USP7’s regulatory functions depend on PKM2. USP7 regulates macrophage polarization in SAP through PKM2-mediated metabolic reprogramming, providing novel insights into the therapeutic targeting of SAP.

## Highlights

- USP7 played an important role for promoting inflammation in SAP mice
- USP7 promoted the M1-type polarization of macrophages in SAP mice
- USP7 promoted the pro-inflammatory phenotype of macrophages by regulating the intracellular function of PKM2
- USP7 regulated PKM2-mediated metabolic reprogramming of macrophages

## Introduction

Severe acute pancreatitis (SAP) is an inflammatory pancreatic disorder with an incidence rate exceeding 1 in 10,000, and it is frequently linked to multiple organ failure, systemic inflammatory response syndrome, and a high mortality [1–3]. However, there is still no therapeutic agents available to interrupt the course of the disease of SAP. Therefore, it is necessary and urgent to explore the pathogenesis of SAP and identify potential effective drugs.

Macrophages, categorized into the pro-inflammatory M1 and the anti-inflammatory M2 phenotypes, are essential inflammatory cells playing crucial roles in the pathogenesis of SAP [4]. In the initial stages of SAP, M1 macrophages infiltrate the pancreatic tissue, exacerbate inflammation through the persistent release of inflammatory mediators. Later on, the infiltration of M2 macrophages counteracts this response by exerting anti-inflammatory effects, thereby curbing the spread of inflammation [4, 5]. Two directions have been considered to be of theoretical and practical value for exploring the treatment options of SAP, including reducing the inflammatory cells infiltration and changing the type of cells infiltrated during SAP [5, 6]. With these insights, it is hypothesized that diminishing M1 macrophage polarization and enhancing M2 macrophage polarization could potentially alleviate SAP.

Accumulating studies have proved that the phenotype of immune cells is closely linked to their metabolism [7–9]. M1 macrophages, known for their pro-inflammatory characteristics, are marked by heightened glycolysis and reduced tricarboxylic acid (TCA) cycle activity, which are typically associated with the oxidative phosphorylation (OXPHOS) system [10, 11]. Furthermore, previous studies verified that downregulating glycolysis inhibits the polarization of macrophages M1, ameliorates inflammatory responses, and restores immune homeostasis [12, 13]. As a key determinant of macrophage metabolic reprogramming, pyruvate kinase M2 (PKM2) can influence glycolysis and OXPHOS by changing the ratio between its inactive and active forms [14, 15]. Among them, the monomeric and dimeric forms of PKM2 (inactive) promote macrophage glycolysis and inflammatory responses, while the tetrameric form of PKM2 (active) induces TCA cycling, OXPHOS and anti-inflammatory responses [14, 15]. Moreover, studies have found that PKM2 affects macrophage function through metabolic reprogramming [16–18]. However, the function of PKM2 in SAP macrophages has not been reported.

Previous studies have shown that ubiquitination modification is a key step in PKM2 phosphorylation and nuclear translocation [19, 20]. Ubiquitin-specific protease 7 (USP7), a key enzyme in the ubiquitination modification process, is widely recognized for its pivotal role in inflammatory responses. It is particularly noted for its involvement in mediating osteoarthritis via the inflammasome pathway and for its regulatory effects on cardiomyocyte pyroptosis triggered by sepsis [21–25]. In particular, USP7 promotes inflammasome activation and inflammatory factor release in macrophages [26]. Also, previous studies have demonstrated that USP7 inhibitors inhibited LPS-induced inflammatory responses by regulating ubiquitin-mediated protein degradation of downstream targets [27].

According to the evidence presented above, a hypothesis suggests that USP7 regulates PKM2 ubiquitination, mediates the metabolic reprogramming of M1 macrophages, and affects pancreatic injury in SAP.

## Materials and methods

### Animal experiments and sampling

The animals used for the current studies were male C57BL/6 N mice at 8–9 weeks of age with 22–25 g body weight purchased from HUNAN SJA Laboratory Animal Co., Ltd. (Hunan, China). Three distinct animal experiments were conducted, with mice randomly allocated to experimental groups. Each experiment was carried out in strict compliance with the guidelines provided in the Guide for the Care and Use of Laboratory Animals and was approved by the Animal Ethics Committee of The First Affiliated Hospital, Jiangxi Medical College, Nanchang University (Jiangxi, China, approval number: CDYFY-IACUC-202407QR227). In Experiment 1, 12 mice were randomly assigned to one of two groups, each consisting of 6 mice: the Sham group and the SAP group. Experiment 2 incorporated more diverse groupings. Here, 24 mice were randomly allocated to one of four groups, with 6 mice in each group: Sham, SAP, SAP plus adeno-associated virus (AAV)-sh-NC (where sh represents short hairpin RNA, and NC represents negative control), and SAP plus AAV-sh-USP7 (with sh-USP7 being the shRNA sequence targeting USP7). Experiment 3, the most extensive of the three, employed five different groups, each with 6 mice. The groups were as follows: Sham, SAP plus AAV-sh-NC, SAP plus AAV-sh-USP7, and SAP plus AAV-sh-USP7 plus Compound 3 K (a PKM2 inhibitor, sourced from APExBIO Technology LLC, B8217, Houston, USA). Each experiment was set up independently, ensuring the integrity and reliability of the results obtained from each grouping. The meticulous design of these experiments aimed at fostering a comprehensive understanding of the respective impacts of the varied conditions on the subject animals.

Sham mice were administered an equivalent volume of normal saline per injection. The SAP models were constructed following the methodology described by Liao et al. [28]. In brief, mice received six hourly caerulein (50 μg/kg) by intraperitoneal injections and then received a single 10 mg/kg lipopolysaccharide (LPS; Escherichia coli 055: B5, L2880, Sigma-Aldrich, USA) intraperitoneal injections after the last caerulein injection [29–31]. For in vivo USP7 knockdown, sh-USP7 was packaged into AAV8 and mice were injected intraperitoneally with AAV-sh-NC or AAV-sh-USP7 (2 × 10^12^ viral genomes/mL in a volume of 100 μL for each mouse; Hanbio Biotechnology, Shanghai, China) for 4 weeks before SAP induction. The dosage form of Compound 3 K was administered orally every 3 days with 5 mg/kg as described by the manufacturer.

All mice were anesthetized with 50 mg/kg pentobarbital 12 h following the final injection. Blood samples were subsequently collected via cardiac puncture to assess serum amylase and lipase activities, as well as cytokine concentrations. The pancreas was meticulously harvested and sectioned into three parts: one portion was fixed with paraformaldehyde and embedded in paraffin for histological analysis, another was immediately processed into a cell suspension for flow cytometry assays, and the final portion was snap-frozen in liquid nitrogen and stored at −80 °C for subsequent qPCR and Western blotting analyses.

### Cell culture and treatment

RAW 264.7 macrophage cells were obtained from the American Type Culture Collection (ATCC, USA), which performed STR authentication and mycoplasma contamination test. Cells were then cultured under meticulously controlled conditions. The culture medium consisted of Dulbecco’s modified Eagle’s medium (DMEM, ATCC) enriched with 10% fetal bovine serum and a combination of antibiotics (100 U/mL penicillin and 100 mg/mL streptomycin). The cells were incubated in an environment with 5% CO_2_ at 37 °C. The macrophages were then subdivided into seven distinct groups, each containing six samples, to facilitate a range of investigative procedures. The groups were as follows: Control, LPS/IFN-γ (treated with a co-induction of 200 ng/mL LPS and 2.5 ng/mL IFN-γ for a duration of 12 h), LPS/IFN-γ plus sh-NC, LPS/IFN-γ plus sh-USP7, IL-4 (treated with 10 ng/mL IL-4 for 12 h), IL-4 plus sh-NC, and IL-4 plus sh-USP7. For the transfection of sh-USP7 (5’-GCC GAA TTT AAC AGA GAG AAT-3’) or sh-NC, the cells were processed with the respective plasmids utilizing the Lipofectamine 3000 transfection kit (Invitrogen, Carlsbad, CA, USA). The transfection was performed in accordance with the manufacturer’s guidelines and was maintained for a period of 48 h.

### Macrophage separation and treatment for SAP mice

The mouse macrophages were obtained according to previously described [32]. Firstly, the mice were euthanized and femurs were dissected, followed by frush with PBS. After centrifugation at 300 × *g* for 5 min at 4 °C, cells were collected and cultured in RPMI-1640 medium containing 10% fetal bovine serum and 30 ng/mL macrophage colony-stimulating factor (M-CSF) for 5–7 days to induce differentiation into macrophages. The macrophages were than infected with lentiviral containing sh-USP7 or sh-NC vectors (Hanbio Biotechnology) and intraperitoneally injected at a dose of 2 × 10^6^ cells per mouse to assess its therapeutic effects in SAP models. Here, 24 mice were randomly allocated to one of four groups, with 6 mice in each group: Sham, SAP, SAP plus sh-NC transducted macrophages, SAP plus sh-USP7 transducted macrophages.

### Histological evaluation

Specimens underwent paraffin embedding before sectioning. Sections of 4 μm thickness were prepared and subsequently underwent hematoxylin-eosin (HE) staining. The histological assessment of pancreatitis involves a semi-quantitative scoring system that evaluates parameters such as edema, inflammation, and necrosis. Edema is scored based on the degree of interlobular and intralobular tissue separation, while inflammation is assessed by the presence and density of inflammatory cell infiltrates. Necrosis is scored by the extent of acinar cell death and tissue breakdown [33, 34]. For immunohistochemistry (IHC), sections were prepared to a thickness of 8 μm. These sections underwent dewaxing with xylene and rehydration through a series of graded alcohol solutions. Following this, they were incubated for 15 min in 3% hydrogen peroxide to quench endogenous peroxidase activity. To prevent nonspecific binding, sections were incubated with 3% bovine serum albumin in PBS for 1 h. Following this, the sections were incubated overnight with anti-USP7 (1:250, ab264422, Abcam, Cambridge, UK) in a saturated humidity chamber maintained at 4 °C. The chromogenic substrate employed was a combination of a biotinylated secondary antibody (1:2000, ab97049, Abcam) and 3,3’-diaminobenzidine, followed by a counterstain with Mayer’s hematoxylin. Sections underwent dehydration and were examined using an Axioskop-2 Plus phase contrast microscope (code: SKU-012, Carl Zeiss, Munich, Germany). Negative controls included tissue sections where primary antibodies were replaced with 2% bovine serum albumin in PBS.

### RNA extraction and quantification

Total RNA was meticulously extracted from tissues and cells utilizing an mRNA extraction kit (Tiangen, Beijing, China). Subsequently, quantitative PCR assays were conducted employing the SYBR Select Master Mix (Applied Biosystems) and 0.5 μL of cDNA, utilizing the ABI7300 system (Applied Biosystems, Foster City, CA, USA), all in strict accordance with the manufacturer’s protocols. The specific primers deployed in the assays are delineated in Table 1. To ascertain relative mRNA expression levels, the 2^-ΔΔCT^ method was employed, with GAPDH serving as the normalization control. This rigorous approach ensures accurate quantification and reliable comparative analysis of mRNA levels across different samples.

**Table 1:** Sequences of primers for qPCR analysis.

Gene	Primer sequence
USP7	Forward: 5′-TCCTCAGCAGTTGGTGGAACGA-3′
	Reverse: 5′-GCCACAAAACTGGTCCTCTGCA-3′
IL-1β	Forward: 5′-TGGACCTTCCAGGATGAGGACA-3′
	Reverse: 5′-GTTCATCTCGGAGCCTGTAGTG-3′
TNF-α	Forward: 5′-GGTGCCTATGTCTCAGCCTCTT-3′
	Reverse: 5′-GCCATAGAACTGATGAGAGGGAG-3′
IL-6	Forward: 5′-TACCACTTCACAAGTCGGAGGC-3′
	Reverse: 5′-CTGCAAGTGCATCATCGTTGTTC-3′
iNOS	Forward: 5′-GAGACAGGGAAGTCTGAAGCAC-3′
	Reverse: 5′-CCAGCAGTAGTTGCTCCTCTTC-3′
CD86	Forward: 5′-ACGTATTGGAAGGAGATTACAGCT-3′
	Reverse: 5′-TCTGTCAGCGTTACTATCCCGC-3′
Arg-1	Forward: 5′-CATTGGCTTGCGAGACGTAGAC-3′
	Reverse: 5′-GCTGAAGGTCTCTTCCATCACC-3′
Fizz1	Forward: 5′-CAAGGAACTTCTTGCCAATCCAG-3′
	Reverse: 5′-CCAAGATCCACAGGCAAAGCCA-3′
CD206	Forward: 5′-GTTCACCTGGAGTGATGGTTCTC-3′
	Reverse: 5’-AGGACATGCCAGGGTCACCTTT-3’
IL-10	Forward: 5′-CGGGAAGACAATAACTGCACCC-3′
	Reverse: 5′-CGGTTAGCAGTATGTTGTCCAGC-3′
GAPDH	Forward: 5′-CATCACTGCCACCCAGAAGACTG-3′
	Reverse: 5′-ATGCCAGTGAGCTTCCCGTTCAG-3′

### Protein extraction and Western blot

Total protein was meticulously extracted from tissues and cells utilizing RIPA lysis buffer (Pierce, Rockford, IL, USA), and concentrations were subsequently quantified using the BCA protein detection kit (Pierce). Proteins were then resolved via Sodium dodecyl sulfate-polyacrylamide gel electrophoresis (SDS-PAGE) and electrotransferred to polyvinylidene fluoride (PVDF) membranes. Post-transfer, membranes were probed with specific primary antibodies and incubated at 4 °C overnight. The primary antibodies used were as follows: anti-USP7 (1:500, #DF6931, Affinity Biosciences, Jiangsu, China), anti-iNOS (1:1000, #AF0199, Affinity Biosciences), anti-CD86 (1:500, #DF6332, Affinity Biosciences), anti-Arg-1 (1:1000, #DF6657, Affinity Biosciences), anti-Fizz1 (1:500, bs-1884R, Bioss, Beijing, China), anti-IL-1β (1:1000, #AF4006, Affinity Biosciences), anti-TNF-α (1:500, #AF7014, Affinity Biosciences), anti-IL-6 (bs-0782R, bs-0782R, Bioss), anti-CD206 (1:500, #DF4149, Affinity Biosciences), anti-IL-10 (1:1000, #DF6894, Affinity Biosciences), anti-GLUT1 (1:200, ab150299, Abcam), anti-HK-2 (1:1000, #DF6176, Affinity Biosciences), anti-PKM2 (1:500, #AF5234, Affinity Biosciences), anti-phospho (p)-PKM2 (1:100, #AF7231 and #DF2975, Affinity Biosciences), anti-LDHA (1:1000, bs-34202R, Bioss) and β-actin (1:2500, ab8227, Abcam). Following incubation, membranes were washed thrice and incubated with the corresponding HRP-conjugated secondary antibodies at room temperature for 1 h. Protein bands were visualized using enhanced chemiluminescence and quantified with Image J software, with β-actin serving as the normalization control. Full and uncropped Western blots, uploaded as “Supplementary materials-Western blot original images”.

### Co-immunoprecipitation (Co-IP) and ubiquitinated IP assay

Co-immunoprecipitation experiments were conducted to elucidate the interaction between USP7 and PKM2. Briefly, cell lysates containing 100 μg of protein were subjected to immunoprecipitation using 5 μg of anti-USP7 or anti-PKM2 antibodies. After an overnight incubation at 4 °C, 30 μL of Protein A agarose bead slurry was added to each sample, which were then gently rocked for 3 h at 4 °C. Subsequently, the samples were centrifuged for 30 s and washed, and 20 μL of SDS buffer was added before heating at 100 °C for 5 min. A 30 μL aliquot of each sample was loaded onto a 10% SDS-PAGE separating gel. Post-electrophoresis, the resolved proteins were transferred to PVDF membranes, blocked, and probed with anti-PKM2 primary antibody for 12 h at 4 °C. The signal was visualized using the electro-chemiluminescence method. Additionally, PKM2 protein, immunoprecipitated by anti-PKM2 from the RAW 264.7 cell lysates (100 μg of protein), was incubated with K48-linked ubiquitin chain peptides, followed by SDS electrophoresis and detection with ubiquitinated antibodies (Proteintech, Wuhan, China). IgG served as the negative control (NC).

### Immunofluorescence assay

The method employed for immunofluorescence staining of pancreatic tissues and RAW 264.7 cells was adapted from a previously described protocol [35]. In brief, sections were initially permeabilized with 0.5% Triton X-100 for 10 min and subsequently washed gently with PBS. This was followed by an incubation period with goat serum for 40 min, and then with primary antibodies such as anti-iNOS (1:100, #AF0199, Affinity Biosciences; 1:100, sc-7271, Santa Cruz Biotechnology), anti-Arg-1 (1:200, #DF6657, Affinity Biosciences; 1:100, sc-271430, Santa Cruz Biotechnology), anti-CD68 (1:200, #DF7518, Affinity Biosciences; 1:100, sc-20060, Santa Cruz Biotechnology), or anti-USP7 (1:25, #DF6931, Affinity Biosciences) diluted in blocking buffer, at 4 °C overnight. Following a 5-min gentle wash with PBS, the sections were exposed to the corresponding fluorescently conjugated secondary antibody for 2 h at room temperature. After another gentle wash with PBS, nuclei were stained with 4,6-diamidino-2-phenylindole (DAPI) for 10 min. Subsequently, the sections were washed, and the coverslips were mounted using a fluorescence decay-resistant mounting medium. Fluorescently labeled cells were visualized and photographed using a confocal laser-scanning microscope (Nikon, Tokyo, Japan).

### Flow cytometry assay

Pancreatic tissues were processed into single-cell suspensions and stained with primary antibodies: anti-F4/80 (1:25, ab6640, Abcam), anti-CD86 (1:600, ab242142, Abcam), and anti-CD206 (0.25 μg, 98031-1-RR, Proteintech). After incubation on ice in the dark for 30 min and washing with PBS, the cells were resuspended in 200 μL of PBS for analysis using a BD FACScan flow cytometer. The gating strategy began with a preliminary gate using FSC-A and FSC-H to include all single cells and exclude debris and doublets. Singlet gating was performed using FSC-A versus FSC-H, followed by the exclusion of dead cells using 7-AAD viability staining. Macrophages were identified as F4/80-positive cells, and subset analysis was conducted by identifying M1 macrophages as CD86-positive and F4/80-positive cells, and M2 macrophages as CD206-positive and F4/80-positive cells.

### Seahorse assay

Experiments were performed using an XF-96 Extracellular Flux Analyzer (Seahorse Bioscience, Agilent Technologies) to assess the Extracellular Acidification Rates (ECAR) and Oxygen Consumption Rates (OCR) of macrophages in vitro. ECAR measurements were taken at baseline and post the sequential addition of 25 mM glucose, 1–2 μM oligomycin, and 50 mM 2-deoxy-D-glucose (2-DG). Conversely, OCR measurements were recorded under basal conditions and subsequent to the addition of 1–2 μM oligomycin, 1.5 μM fluoro-carbonyl cyanide phenylhydrazone, 200 μM etomoxir, and a combination of 100 nM rotenone and 1 μM antimycin A; all reagents were procured from Sigma. Furthermore, biochemical kits were employed to quantify glucose consumption (E-BC-K234-M, Elabscience, Wuhan, China), lactate levels (E-BC-K044-M, Elabscience), and Lactate Dehydrogenase (LDH) activity (E-BC-K046-M, Elabscience), all in strict accordance with the manufacturers’ instructions.

### Enzyme-linked immunosorbent assay (ELISA)

Commercial ELISA kits, following the manufacturer’s instructions (Elabscience), were employed to quantify the serum concentrations of IL-1β (E-HSEL-M0001), TNF-α (E-EL-M3063) and IL-6 (E-EL-M0044c), as well as activities of amylase (E-BC-K007-M) and lipase (E-BC-K786-M).

### Statistical analysis

Data were represented as mean ± standard deviation (SD). Statistical analyses were conducted utilizing GraphPad Prism 8.0 software. For comparisons between two groups, a two-tailed and unpaired Student’s *t* test was employed. When comparing more than two groups, a one-way analysis of variance (ANOVA) followed by a Tukey’s test was utilized. The results of cell culture experiments were obtained from at least three independent replicates. For animal study, six mice per group were used. The sample size was predetermined based on published literatures and previous lab experience. No statistical methods were used to predetermine the sample size. No samples were excluded from the analysis. The investigator was blinded to the group allocation during the experiment and/or whenassessing the outcome. Normal distribution of data was verified using a Shapiro–Wilkinson test. The variance was similar between the groups that were being statistically compared. A *p* < 0.05 was deemed indicative of statistical significance in all analyses performed.

## Results

### USP7 is highly expressed in the pancreatic macrophages of SAP mice

**Fig. 1:**
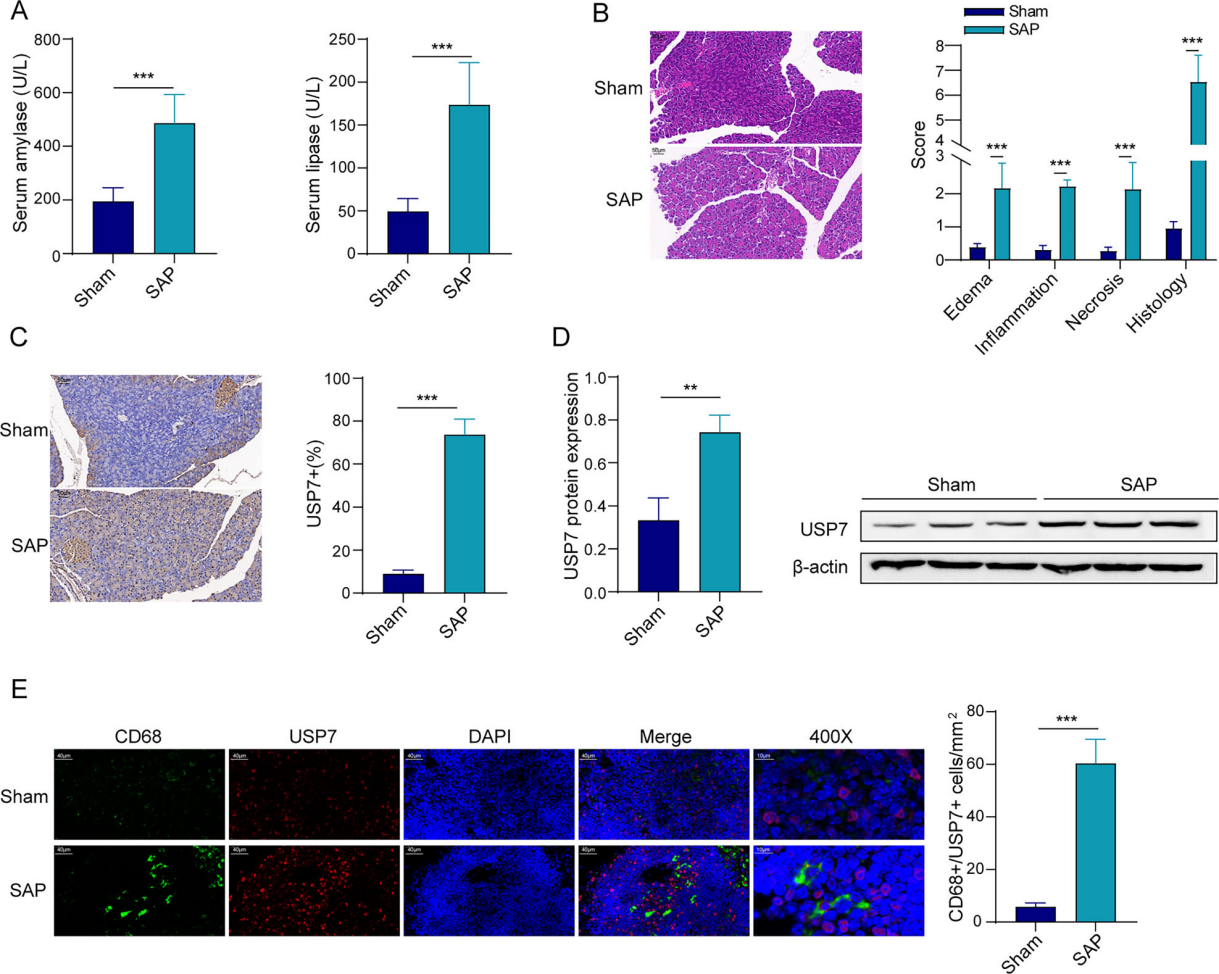
USP7 was highly expressed in the pancreatic macrophages of SAP mice. **A** Serum amylase and lipase activities from the ELISA. **B** Pancreatic tissue damage according to the HE staining. **C** USP7 expression in pancreatic tissues based on the IHC staining. **D** Representation of USP7 protein expression from the results of Western blot from three independent experiments. **E** CD68 and USP7 were expressed in similar positions in pancreatic tissues according to immunofluorescence. *n* = 3 for (**D**), *n* = 6 for (**A**–**C**, **E**). **p < 0.01, ***p < 0.001.

To analyze the relationship between USP7 and SAP, USP7 expression was evaluated in the mouse model of SAP. As shown in Fig. 1A, SAP induction significantly increased serum activities of amylase and lipase when compared with the sham group. Moreover, pancreatic tissues from SAP mice showed more pronounced edema, inflammation, and necrosis, along with higher histological scores compared those pancreatic tissues from sham group mice (Fig. 1B). These results indicated a non-surgical SAP model was successfully established. Additionally, USP7 expression was significantly upregulated in pancreatic tissues of SAP mice according to the results from IHC and Western blot (Fig. 1C, D). Particularly, dual immunofluorescence staining revealed significantly higher expression of USP7 in macrophages (CD68^+^) that had infiltrated the pancreas of SAP mice, as compared to the sham group (Fig. 1E). Taken together, USP7 was highly expressed in SAP pancreatic tissue and expressed in macrophages.

### USP7 knockdown alleviates SAP in mice

**Fig. 2:**
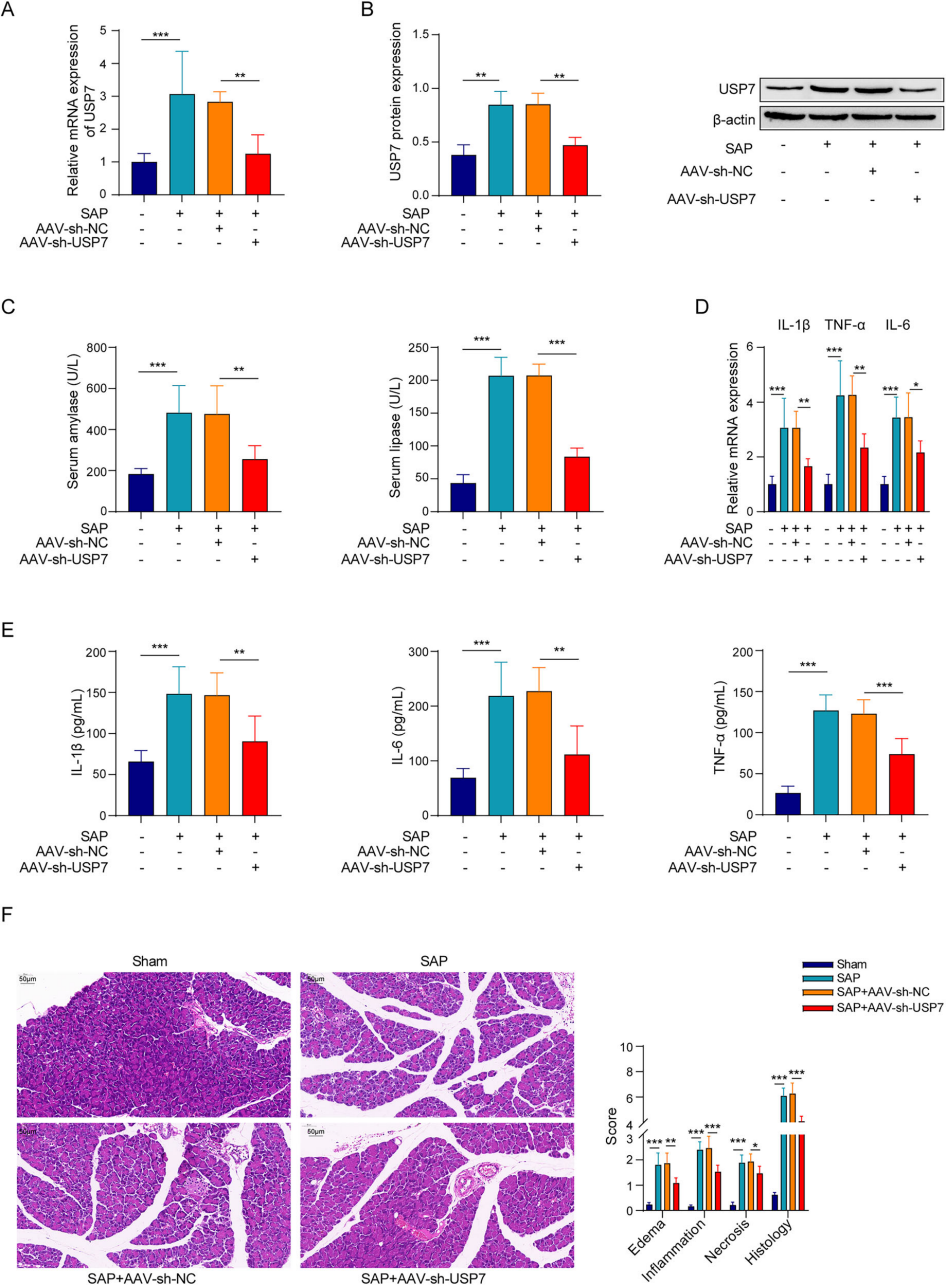
USP7 knockdown alleviated SAP in mice. **A** USP7 mRNA expression based on the qPCR. **B** USP7 protein expression based on the Western blot. **C** Serum amylase and lipase activities from the ELISA. **D** IL-1β, TNF-α, IL-6 mRNA expression in pancreatic tissues from the results of qPCR. **E** Serum concentrations of IL-1β, TNF-α and IL-6 from the ELISA. **F** Pancreatic tissue damage according to the HE staining. *n* = 3 for (**B**), *n* = 6 for (**A**, **C**–**F**). *p < 0.05, **p < 0.01, ***p < 0.001.

To verify whether USP7 had an effect on SAP, AAV-sh-USP7 was used to inhibit USP7 expression in SAP model. AAV-sh-USP7 significantly downregulated USP7 expression in mRNA and protein levels in the pancreas tissues of SAP mice (Fig. 2A, B), indicating the knockdown of USP7 was successfully performed. Phenotypically, USP7 inhibition decreased the serum activities of amylase and lipase (Fig. 2C), as well as the reduced pancreatic mRNA expression and serum concentration of main pro-inflammatory cytokines including IL-1β, TNF-α and IL-6 (Fig. 2D, E). Furthermore, USP7 knockdown resulted in a significant reduction of pancreatic histology scores in SAP mice, including significant alleviation in edema, inflammation, and necrosis (Fig. 2F). For further specify the impact of USP7 downregulation in macrophage on the progression of SAP, we separated macrophages in vitro, knocked down USP7, and injected into SAP mice. Similar effects such as decreased inflammatory cytokines in serum and ameliorated pancreatic histology were also observed (Fig. S1). Therefore, the results mentioned above showed that inhibition of USP7 could alleviate SAP.

### USP7 regulates macrophage polarization in the pancreatic tissues of SAP mice

**Fig. 3:**
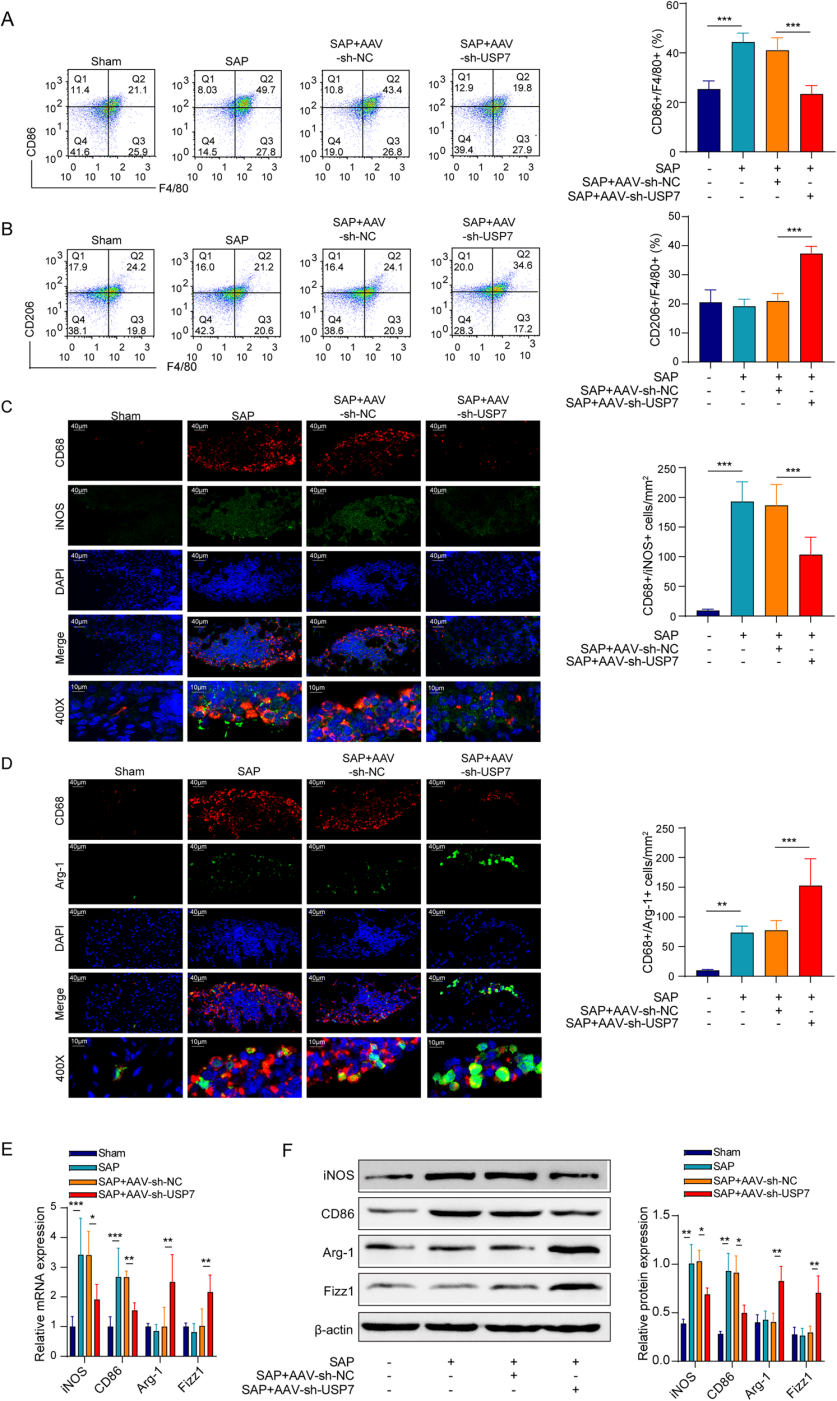
USP7 regulated macrophage polarization in the pancreatic tissues of SAP mice. **A**, **B** Pancreatic macrophages were isolated and the proportion of CD86/F4/80 and CD206/F4/80 positive cells was measured by flow cytometry, respectively. **C** Analysis of the number of M1 macrophages in pancreatic tissues based on immunofluorescence detection of CD68/iNOS. **D** Analysis of the number of M2 macrophages in pancreatic tissues based on immunofluorescence detection of CD68/Arg-1. **E** iNOS, CD86, Arg-1 and Fizz1 mRNA expression in pancreatic tissues from the results of qPCR. **F** iNOS, CD86, Arg-1 and Fizz1 protein levels in pancreatic tissues from the results of Western blot. *n* = 3 for (**F**), *n* = 6 for (**A**–**E**). *p < 0.05, **p < 0.01, ***p < 0.001.

As previously discussed, USP7 deficiency alleviates inflammation in pancreatic tissues, indicating that USP7 may play a role in modulating macrophage polarization. After the induction of SAP, USP7 silencing decreased the ratio of CD86^+^ F4/80^+^ cells and increased the ratio of CD206^+^ F4/80^+^ cells in macrophages infiltrating the pancreas (Fig. 3A, B). Furthermore, USP7 inhibition reduced the number of M1 type (CD68^+^/iNOS^+^) macrophages and expanded the number of M2 type (CD68^+^/Arg-1^+^) macrophages in the pancreatic tissues of SAP mice (Fig. 3C, D). In addition, USP7 knockdown downregulated the expression of M1 macrophage markers including iNOS and CD86, and upregulated the expression of M2 macrophage markers including Arg-1 and Fizz1 in the pancreatic tissues of SAP mice in the levels of mRNA and protein, respectively (Fig. 3E, F). Collectively, USP7 knockdown inhibited the M1 polarization and promoted the M2 polarization of macrophages in pancreatic tissues of SAP mice.

### USP7 affects the macrophage phenotype in vitro

**Fig. 4:**
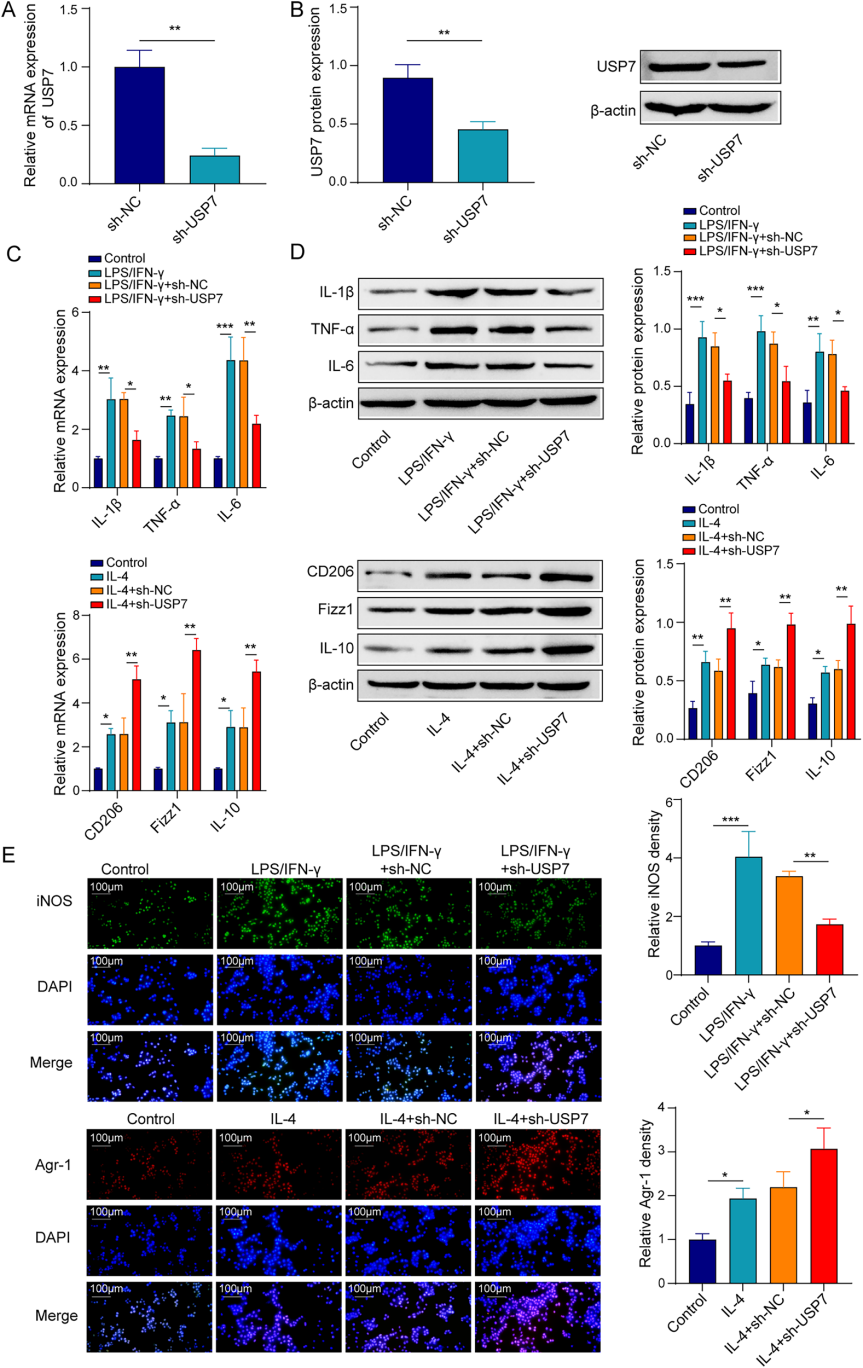
USP7 affected the macrophage phenotype in vitro. **A** USP7 mRNA expression from the results of qPCR. **B** USP7 protein level from the results of Western blot. **C** iNOS, CD86, Arg-1, Fizz1, IL-1β, TNF-α and IL-6 mRNA expression from the results of qPCR in RAW 264.7 macrophages. **D** iNOS, CD86, Arg-1, Fizz1, IL-1β, TNF-α and IL-6 protein levels from the results of Western blot in RAW 264.7 cells. **E** Immunofluorescence of iNOS and Arg-1 in RAW 264.7 cells. *n* = 3. *p < 0.05, **p < 0.01, ***p < 0.001.

Mouse monocytic macrophage leukemia cell line RAW264.7 was used to validate the intervention effect of USP7 on macrophage phenotyping in vitro. Compared to the sh-NC transfection, the sh-USP7 plasmid transfection inhibited the USP7 mRNA and protein expression in RAW264.7 cells (Fig. 4A, B). In addition, LPS/IFN-γ treatment significantly upregulated the expression of pro-inflammatory markers (M1 type) including IL-1β, TNF-α and IL-6 in RAW264.7 cells, while USP7 knockdown partly reversed the treatment effect of LPS/IFN-γ. In addition, IL-4 treatment significantly upregulated the expression of anti-inflammatory markers (M2 type) including CD206, Fizz1, and IL-10 in RAW264.7 cells, and sh-USP7 enhanced the treatment effect of IL-4 on RAW264.7 cells (Fig. 4C, D). Similarly, sh-USP7 decreased the fluorescence intensity of the M1-type polarization marker iNOS whereas enhanced the fluorescence intensity of the M2-type polarization marker Arg-1 under the LPS/IFN-γ and IL-4 treatment (Fig. 4E). Briefly, USP7 regulated the macrophage phenotype in vitro.

### USP7 modulates metabolic reprogramming of the M1 macrophages

**Fig. 5:**
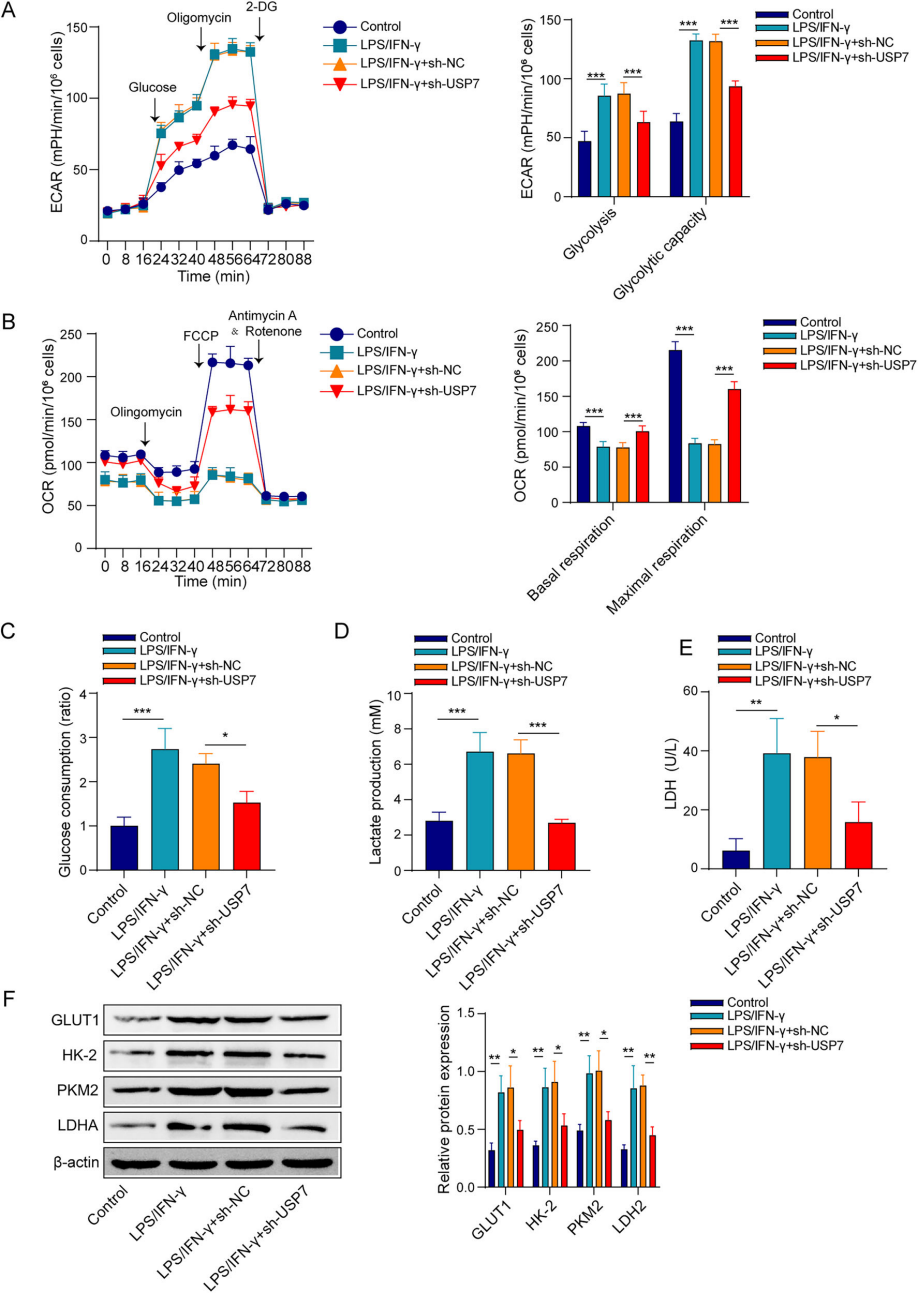
USP7 modulated metabolic reprogramming of the M1 macrophages. **A**, **B** ECAR and OCR analysis from the results of Seahorse assay, respectively. **C**–**E** The results of glucose consumption, lactate production, LDH activity based on biochemical kits. **F** GLUT1, HK-2, PKM2 and LDHA protein expression from the results of Western blot. *n* = 3. *p < 0.05, **p < 0.01, ***p < 0.001.

The ECAR and OCR of RAW264.7 cells under LPS/IFN-γ treatment were measured to assess the glycolytic activity and mitochondrial respiration characteristic of M1-type macrophages, respectively. After LPS/IFN-γ induction, RAW264.7 macrophages exhibited typical M1 macrophage metabolic characteristics, including increased glycolysis and glycolytic capacity, but decreased basal and maximal respiration (Fig. 5A, B). However, sh-USP7 partially reversed the treatment effect of LPS/IFN-γ on the metabolic characteristics of RAW264.7 macrophages (Fig. 5A, B). Moreover, sh-USP7 inhibited the increased glucose consumption, lactate production and LDH activity under the LPS/IFN-γ treatment (Fig. 5C–E). Also, LPS/IFN-γ treatment upregulated the protein expression of GLUT1, HK-2, PKM2, LDHA (lactate dehydrogenase α) in RAW264.7, which were suppressed by the sh-USP7 transfection (Fig. 5F).

### USP7 mediates PKM2 deubiquitination and alteres its phosphorylation and nuclear translocation

**Fig. 6:**
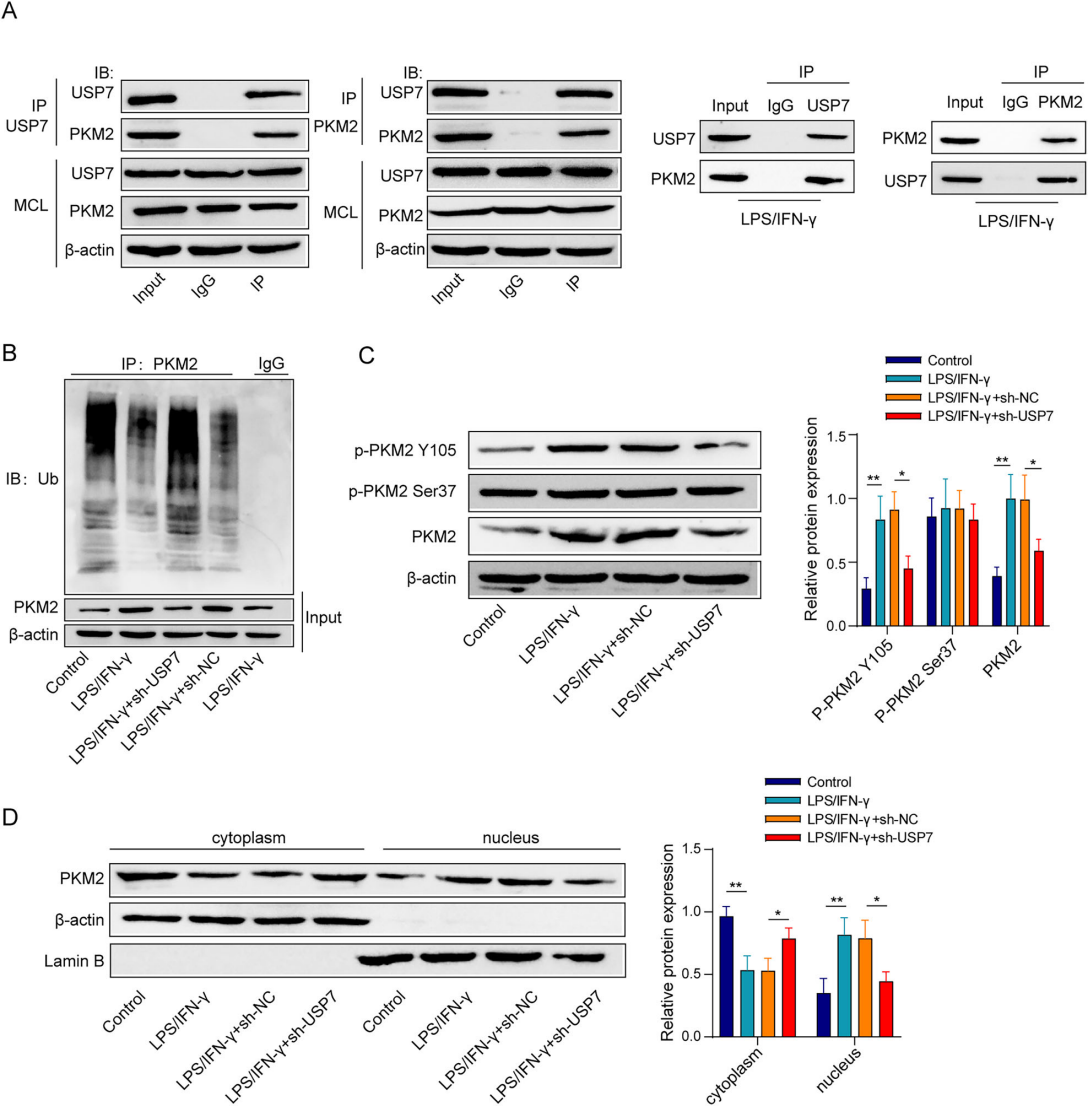
USP7 mediated PKM2 deubiquitination and altered its phosphorylation and nuclear translocation. **A** The interaction between USP7 and PKM2 was identified based on the Co-IP results. **B** Ubiquitination level of PKM2 protein based on the results from the immunoprecipitation and ubiquitinated antibody detection. **C** PKM2 and p-PKM2 protein levels from the results of Western blot. **D** PKM2 protein expression in the cell nucleus and cytoplasm from the results of Western blot. *n* = 3. *p < 0.05, **p < 0.01.

As shown in Fig. 6A, the interaction relationship between USP7 and PKM2 was identified based on the result from Co-IP under normal and acute inflammation conditions. In addition, sh-USP7 promoted PKM2 ubiquitination, which partly reversed the inhibitory effect of LPS/IFN-γ on PKM2 ubiquitination (Fig. 6B). Moreover, LPS/IFN-γ promoted the phosphorylation of PKM2 Y105, but had no effect on Ser37 phosphorylation, and sh-USP7 could inhibit the phosphorylation of PKM2 Y105 induced by LPS/IFN-γ (Fig. 6C). Furthermore, LPS/IFN-γ induction promoted PKM2 transfer from cytoplasm to nucleus, but sh-USP7 inhibited PKM2 transferring into nucleus (Fig. 6D). Together, USP7 directly promoted phosphorylation and nuclear translocation of PKM2 by mediating its deubiquitination.

### PKM2 inhibitor partially reverses the protective effect of USP7 knockdown on SAP

**Fig. 7:**
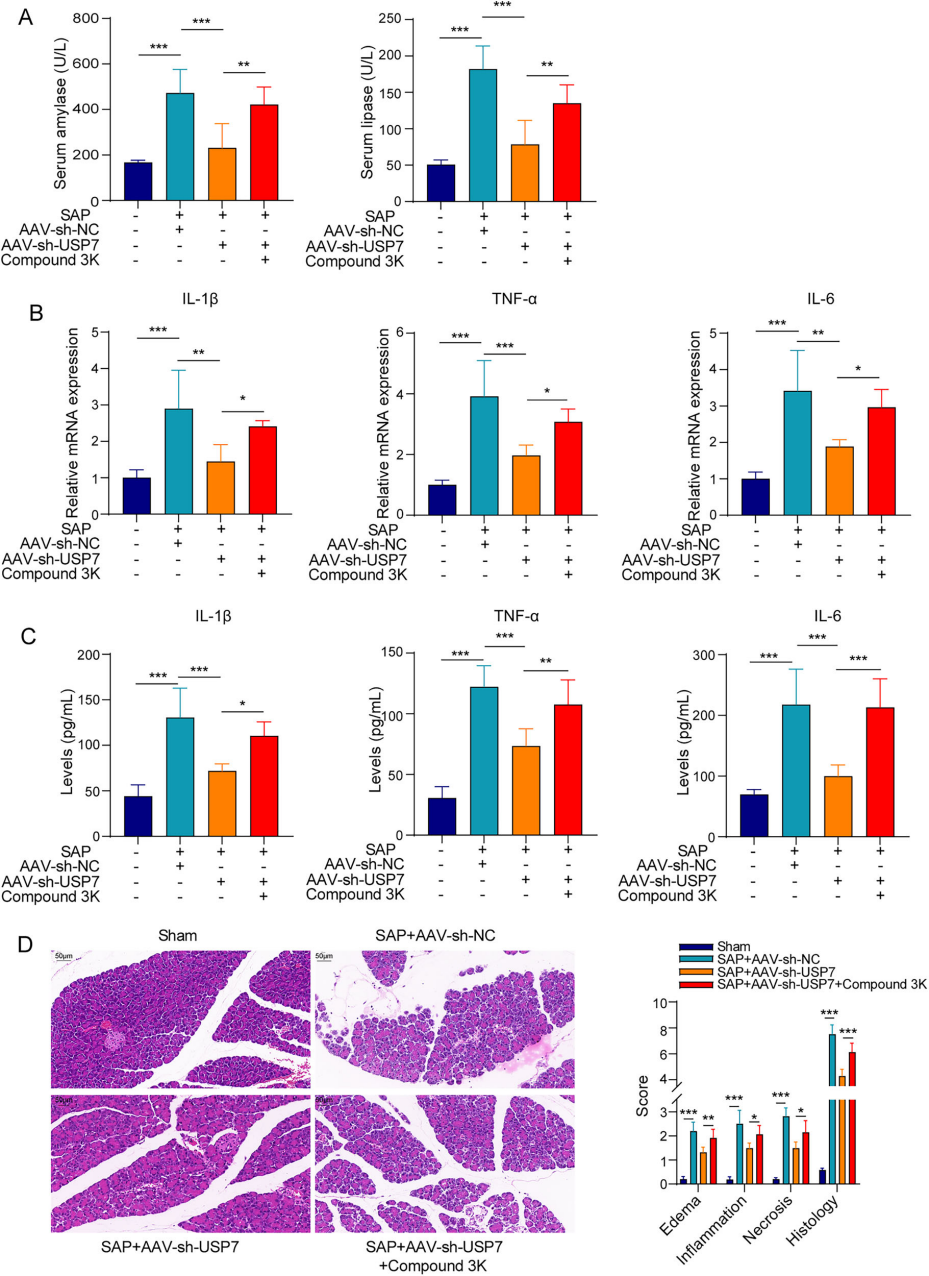
PKM2 inhibitor partly reversed the protective effect of USP7 knockdown on SAP. **A** Serum amylase and lipase activities from the ELISA. **B** IL-1β, TNF-α, IL-6 mRNA expression in pancreatic tissues from the results of qPCR. **C** Serum concentrations of IL-1β, TNF-α and IL-6 from the ELISA. **D** Pancreatic tissue damage according to the HE staining. *n* = 6. *p < 0.05, **p < 0.01, ***p < 0.001.

As shown at Fig. 7A, the PKM2 inhibitor Compound 3 K significantly increased serum amylase and lipase activities in AAV-sh-USP7 treated SAP mice. In addition, Compound 3 K subverted the inhibitory effect of AAV-sh-USP7 on IL-1β, TNF-α, IL-6 mRNA expression in pancreatic tissues and serum inflammatory factor levels from SAP mice (Fig. 7B, C). Moreover, the alleviating effect of AAV-sh-USP7 on edema, inflammatory cell infiltration and necrosis of pancreatic tissues in SAP mice was also limited under Compound 3 K treatment (Fig. 7C, D). Totally, PKM2 was crucial to the protective effect of USP7 knockdown on SAP.

### PKM2 inhibitor affectes the regulatory effect of USP7 knockdown on macrophage polarization in SAP pancreatic tissues

**Fig. 8:**
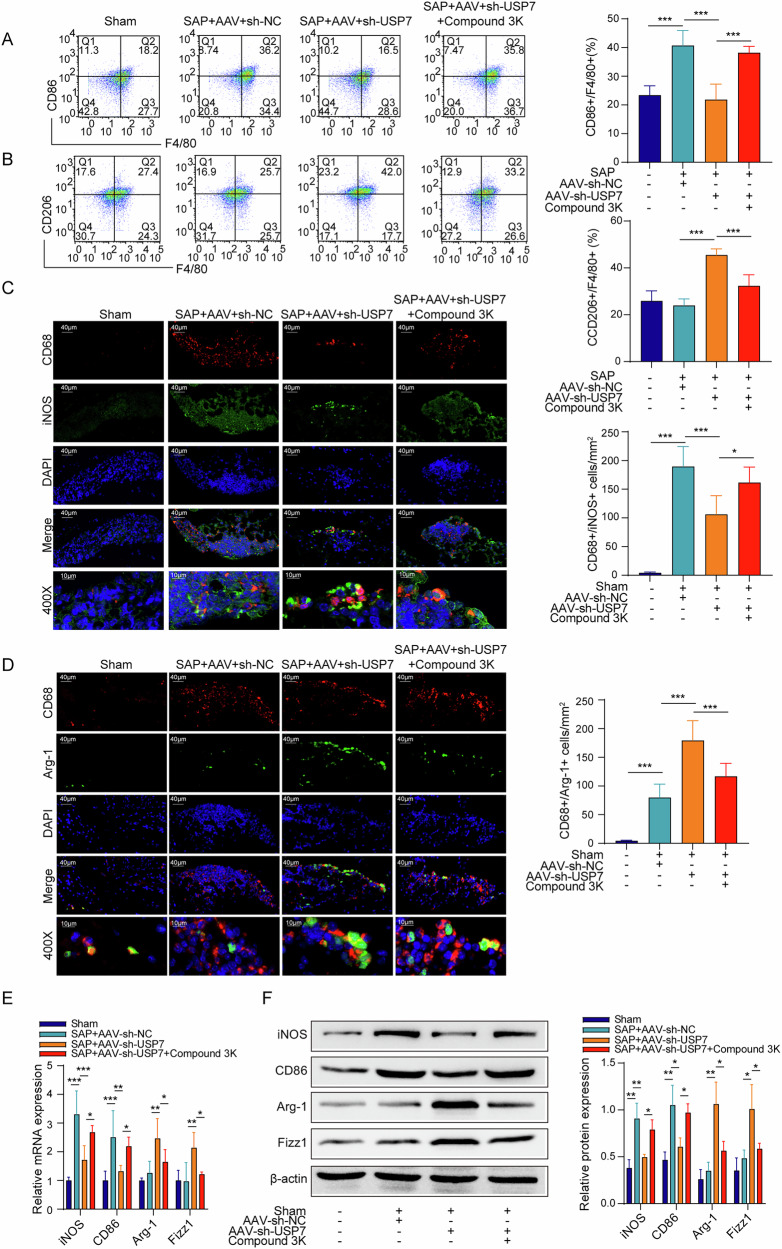
PKM2 inhibitor affected the regulatory effect of USP7 knockdown on macrophage polarization in SAP pancreatic tissues. **A**, **B** Pancreatic macrophages were isolated and the proportion of CD86/F4/80 and CD206/F4/80 positive cells was measured by flow cytometry, respectively. **C** Analysis of the number of M1 macrophages in pancreatic tissues based on immunofluorescence detection of CD68/iNOS. **D** Analysis of the number of M2 macrophages in pancreatic tissues based on immunofluorescence detection of CD68/Arg-1. **E** iNOS, CD86, Arg-1 and Fizz1 mRNA expression in pancreatic tissues from the results of qPCR. **F** iNOS, CD86, Arg-1 and Fizz1 protein levels in pancreatic tissues from the results of Western blot. *n* = 3 for (**F**), n = 6 for (**A**–**E**). *p < 0.05, **p < 0.01, ***p < 0.001.

As shown in Fig. 8A, B, Compound 3 K partially reversed the influence of AAV-sh-USP7 on the proportion of CD86/F4/80-positive cells and CD206/F4/80-positive cells in pancreatic macrophages of SAP mice. Also, Compound 3 K partially reversed the effect of AAV-sh-USP7 on the number of M1 (CD68^+^/iNOS^+^) macrophages and M2 type (CD68^+^/Arg-1^+^) macrophages in the pancreatic tissues of SAP mice (Fig. 8C, D). Moreover, Compound 3 K remarkably repressed the regulatory effect of USP7 knockdown on iNOS, CD86, Arg-1, Fizz1 mRNA and protein levels in pancreatic tissues of SAP mice (Fig. 8E, F). These results validate that the regulation of USP7 in macrophage polarization was obviously dependent on PKM2.

## Discussion

SAP is an acute condition characterized by a high mortality rate, posing a significant threat to human health and safety. Previous studies have identified macrophage infiltration associated with the intense inflammatory response as a key factor in the pathogenesis of SAP [36, 37]. In the current study, USP7 was identified as a key protein influencing the macrophage pro-inflammatory phenotype both in SAP mice and macrophage in vitro. Furthermore, it was identified that USP7 regulated the macrophage polarization via PKM2 mediated metabolic reprogramming.

It is widely recognized that macrophage polarization and SAP are closely related [36–39]. Emphatically, M1-type polarization of macrophages directly promotes the inflammatory response, a key factor contributing to tissue damage in patients with SAP, whereas M2-type polarization exhibits anti-inflammatory effects that counteract those of M1 macrophages in this context [40, 41]. Therefore, the polarized phenotype of macrophages was an important target for SAP therapy. For instance, abdominal paracentesis drainage ameliorated SAP by regulating the polarization of peritoneal macrophages in rat model [42]. Similarly, paeonol inhibited the M1 macrophage polarization thereby reduced serum levels of amylase, lipase, IL-1β and IL-6 and alleviated the histopathological manifestations of pancreatic tissue in a mild acute pancreatitis model [43]. As a protein associated with ubiquitination modifications, USP7 was involved in the inflammatory response of macrophages. USP7 could modulate the anti-tumor immune response by reprogramming tumor-associated macrophages in lung cancer [26]. Inhibition of USP7 results in phenotypical and functional changes in M2 macrophages, favoring the proliferation of CD8^+^ T cells and promoting the infiltration of M1 macrophages and IFN-γ^+^CD8^+^ T cells [26]. USP7 inhibitor attenuated the release of pro-inflammatory factors, suppresses mRNA expression of pro-inflammatory enzymes, and inhibits signaling pathways related to inflammation in macrophages stimulated with LPS [44]. Our study firstly confirmed that USP7 affected SAP by regulating macrophage polarization, and USP7-knockdown macrophages can also achieve the therapeutic effects in SAP models. Further, the results also showed that USP7 knockdown alters the metabolic characteristics of M1 macrophages, which are hallmarked by decreased glycolysis and reduced LDH activity.

Increasing studies have proved that the phenotype of immune cells is closely linked with their metabolism [7, 8, 45]. Macrophage metabolic reprogramming influences the inflammatory response and disease progression. This reprogramming involves shifts between glycolysis and oxidative phosphorylation, which are essential for the activation and function of M1 and M2 macrophages [46]. The pro-inflammatory M1 macrophages, characterized by increased glycolysis, contribute to the exacerbation of inflammation in SAP, while M2 macrophages, with their elevated oxidative phosphorylation, play a role in resolution and repair [14, 47]. PKM2 is an isoenzyme of the glycolytic enzyme pyruvate kinase and an important regulator of aerobic glycolysis, promoting lactate production and metabolic reprogramming [20]. Previous studies have shown that deubiquitination of PKM2 promoted glycolysis and exacerbated inflammation and the progression of cancer [35, 48]. Recent studies revealed the critical role of ubiquitination and phosphorylation of PKM2 in cancer progression and metabolic alterations, notably in driving aerobic glycolysis and tumor growth through complex molecular interactions and regulatory mechanisms [49, 50]. The regulation of macrophage phenotype by PKM2 has also been extensively demonstrated [16–18, 51]. However, there are still no studies showing the ubiquitination of PKM2 in macrophages and the mechanisms affecting metabolic reprogramming. Our results demonstrate that USP7 directly influences PKM2 function through deubiquitination, which in turn affects the phosphorylation and nucleation status of PKM2. Specifically, we observed that USP7-mediated deubiquitination of PKM2 promotes the M1 phenotype, which is associated with pro-inflammatory responses, by enhancing glycolysis and suppressing oxidative phosphorylation. This is consistent with the established role of PKM2 in modulating the Warburg effect, which is often observed in activated immune cells and is characterized by increased glucose uptake and lactate production. Furthermore, our data suggest that USP7 is also involved in the regulation of PKM2 nuclear translocation, which has been implicated in the control of gene expression and inflammatory signaling. The introduction of compound 3 K, a widely verified PKM2 inhibitor [52, 53], has effectively reversed the impacts of sh-USP7 on the SAP mice, which further demonstrated the USP7 knockdown protects SAP mice by interfering PKM2 activity. These findings provide a novel perspective on the interplay between USP7, PKM2, and macrophage metabolic reprogramming in SAP.

There are several limitations in this study. Firstly, while our experiments provide evidence for the role of USP7 in regulating macrophage polarization in SAP, the study is primarily focused on a murine model, which may not fully replicate the complexity of human SAP. The translation of these findings to human pathology will require further validation in clinical samples. Secondly, although our study highlights USP7’s role in macrophage polarization, emerging evidence suggests its broader pathological impact across multiple pancreatic cell types during SAP. USP7 knockdown reduces inflammation and pancreatic acinar cell injury during acute pancreatitis progression by inhibiting autophagy activation [54]; additionally, USP7 is an essential regulator for driving pancreatic endocrine lineage development, which may cause post-pancreatitis diabetes mellitus [55, 56]. Therefore, the potential impact of USP7 on other cell populations in the pancreas upon SAP should be further addressed.

**Fig. 9:**
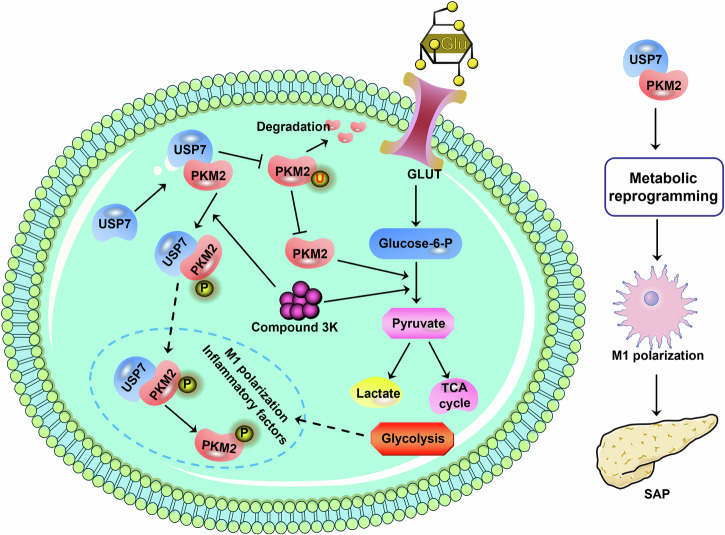
Schematic illustration how USP7 regulates the macrophage polarization via PKM2-mediated metabolic reprogramming in SAP. USP7 regulates the metabolic reprogramming of M1 macrophages by mediating PKM2 deubiquitination, thereby affecting its phosphorylation and nuclear translocation.

In summary (Fig. 9), the current study provided evidence that USP7 affected macrophage polarization by intervening in metabolic reprogramming, and aggravated pancreatic injury of SAP mice. Mechanistically, we found that USP7 altered metabolic reprogramming of macrophages via the deubiquitination of PKM2, thereby promoting pro-inflammatory M1-type polarization in SAP mice.

## Supplementary information

Figure S1

Western blot original images

## Data Availability

The datasets used or analyzed during the current study are available from the corresponding author on reasonable request.
