# Supplementary material for: Ubiquitin-specific protease 7 regulates macrophage polarization via pyruvate kinase M2-mediated metabolic reprogramming in severe acute pancreatitis
Source: Cell Death Dis. 2025 Oct 27;16(1):764. doi: 10.1038/s41419-025-08081-2 (PMC12559371; doi:10.1038/s41419-025-08081-2)

**Figure S1. The therapeutic effects of USP7-knockdown macrophages on SAP.**

(A-B) Serum amylase and lipase activity from the ELISA. (C-E) Serum concentrations of IL-1β, TNF-α and IL-6 from the ELISA. (F) Pancreatic tissue damage according to the HE staining. (G) iNOS, CD86, and Arg-1 protein levels in pancreatic tissues from the results of Western blot. n = 3 for (G), n = 6 for (A-F). ***p* <0.01, ****p* <0.001.


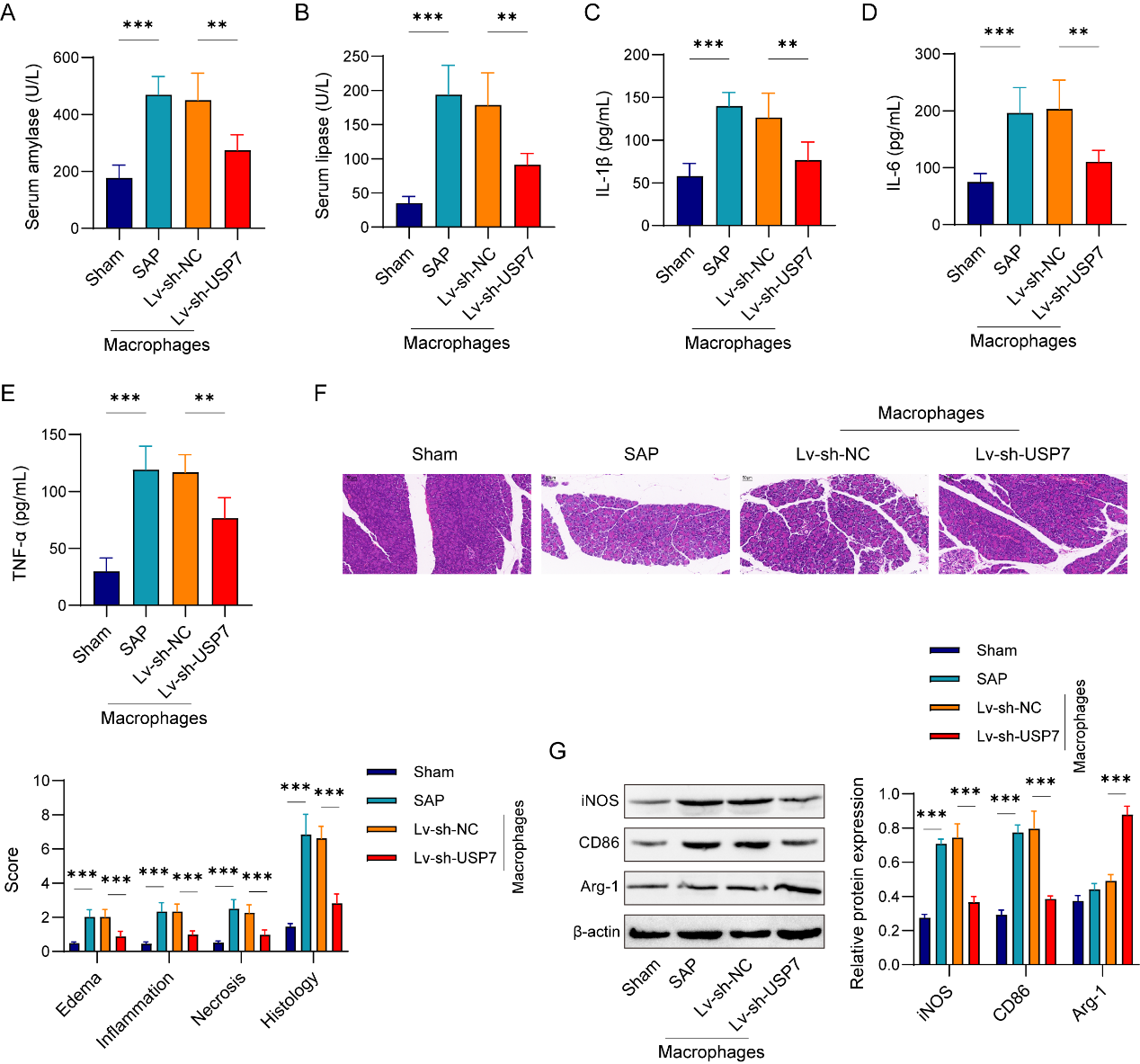

Supplement: Supplementary file 1 — Figure S1 [file 41419_2025_8081_MOESM1_ESM.docx]
