# Supplementary material for: Ubiquitin-specific protease 7 regulates macrophage polarization via pyruvate kinase M2-mediated metabolic reprogramming in severe acute pancreatitis
Source: Cell Death Dis. 2025 Oct 27;16(1):764. doi: 10.1038/s41419-025-08081-2 (PMC12559371; doi:10.1038/s41419-025-08081-2)
Supplement: Supplementary file 2 — Western blot original images [file 41419_2025_8081_MOESM2_ESM.docx]

**Western blot original images**

**Figure 1D**

USP7


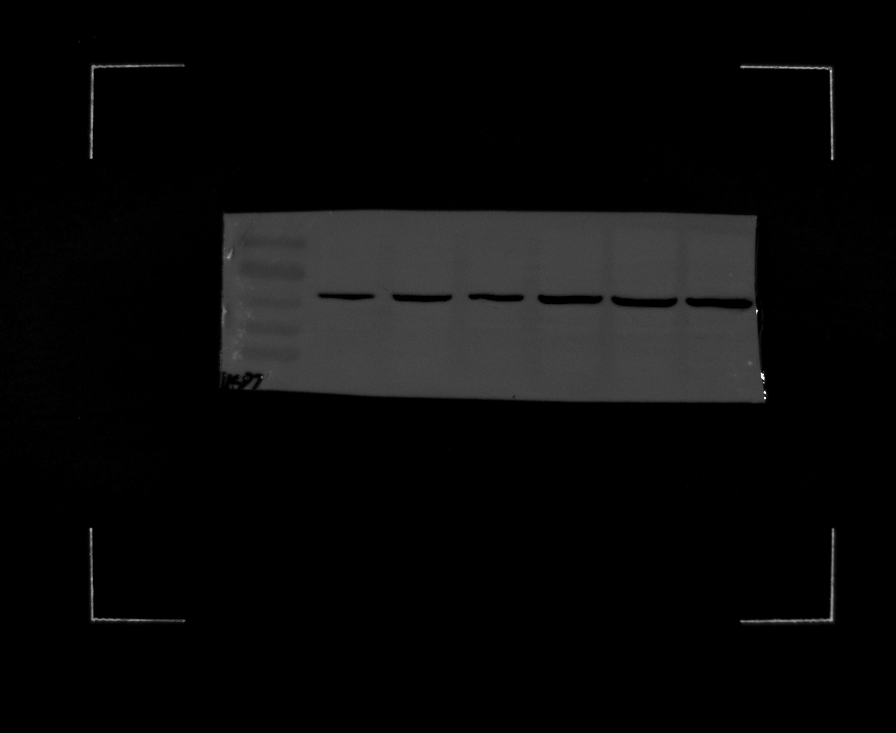

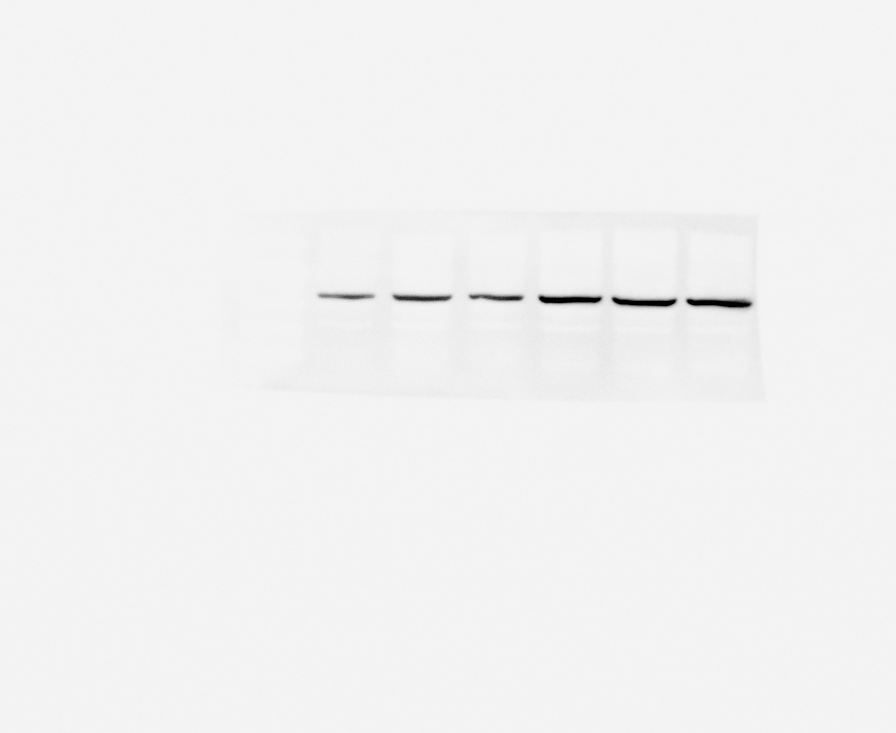


β-actin


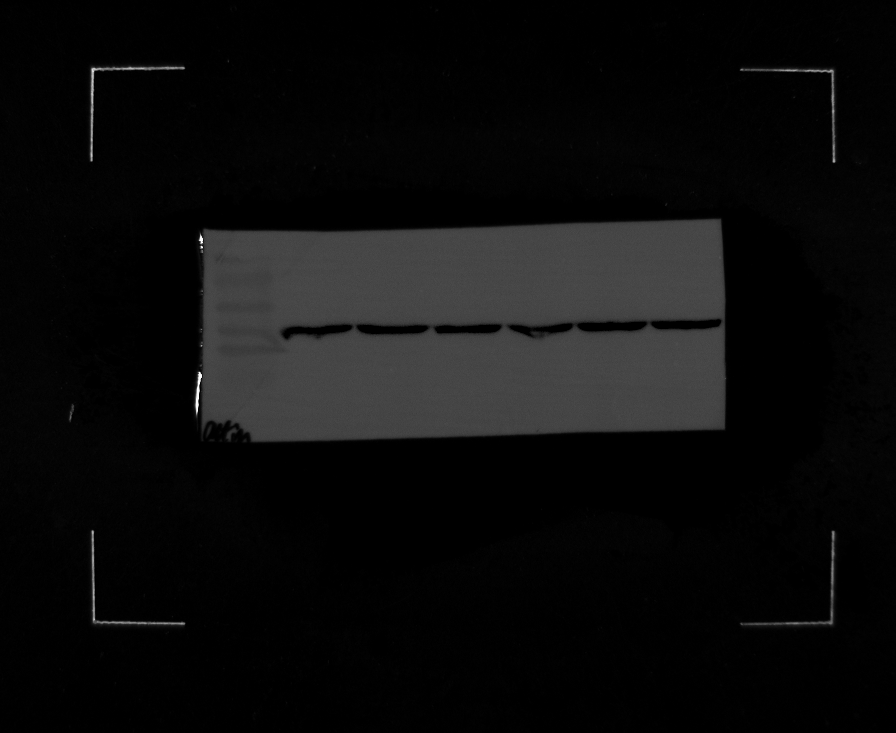

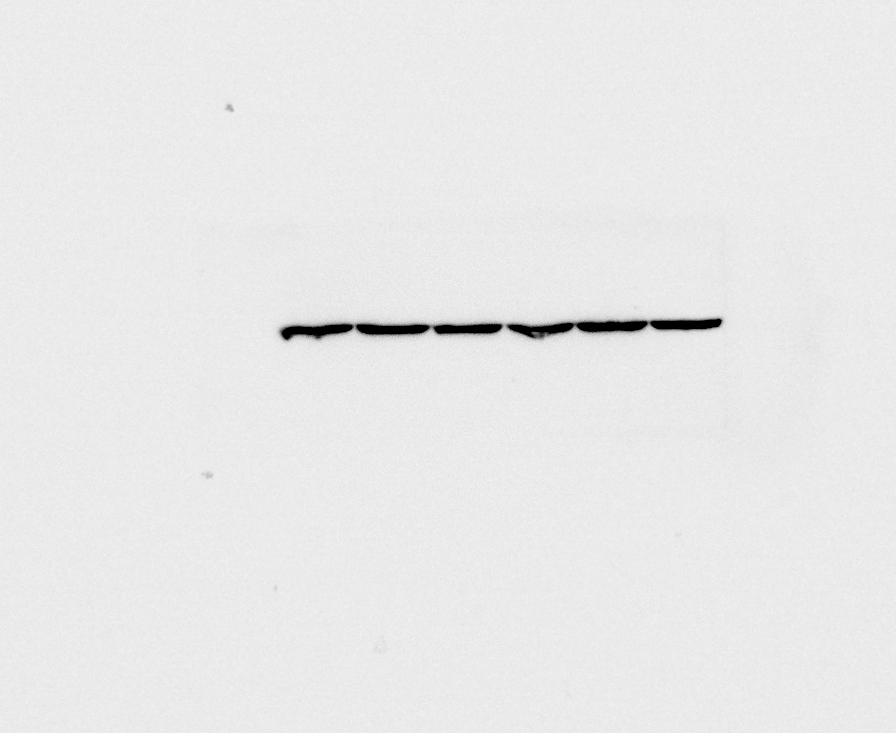


**Figure 2B**

USP7


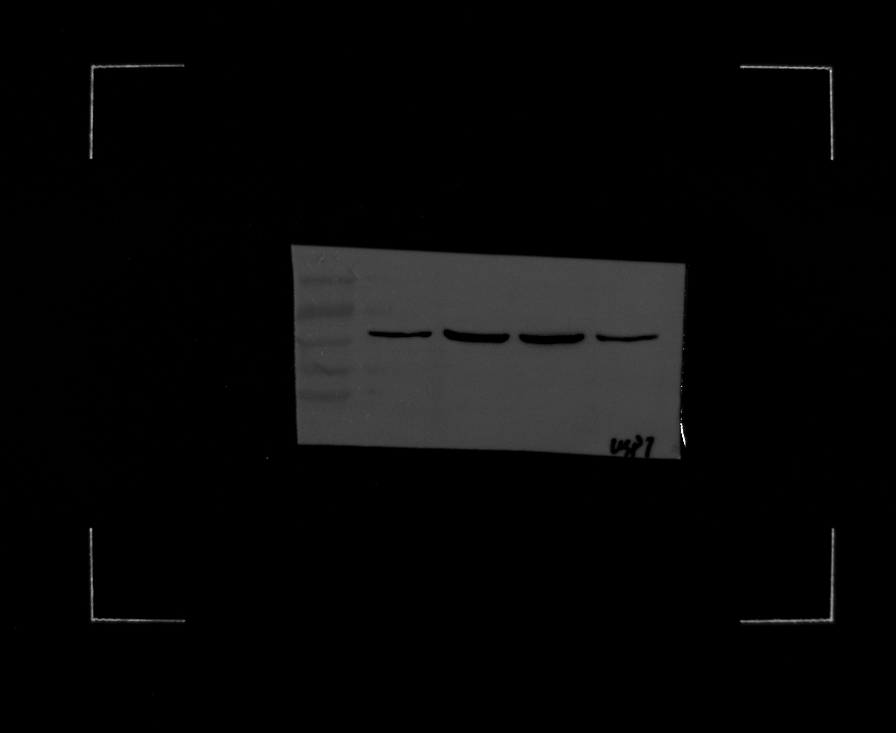

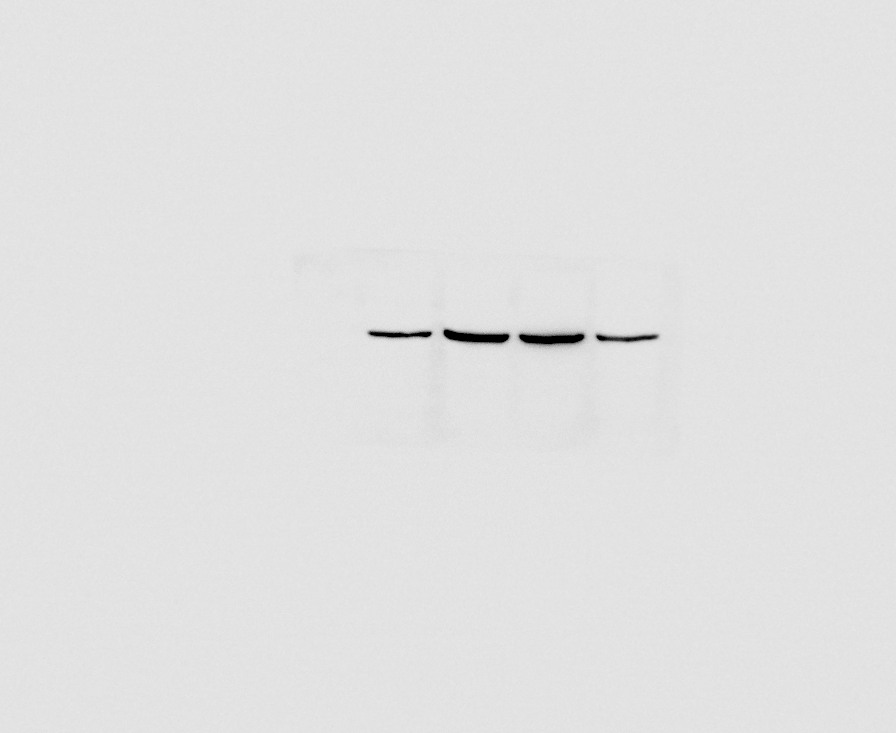


β-actin


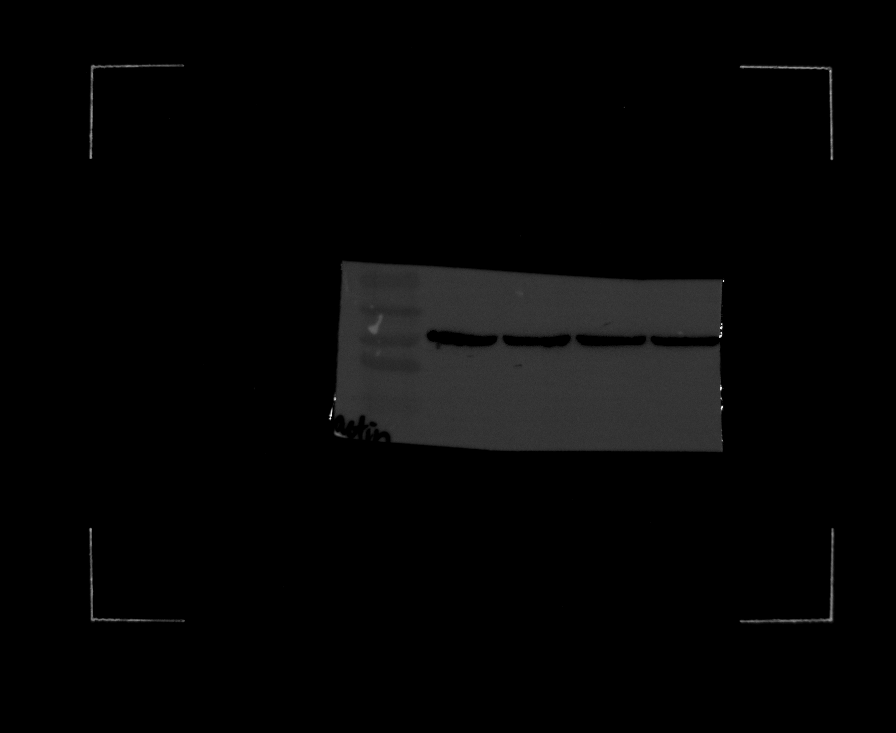

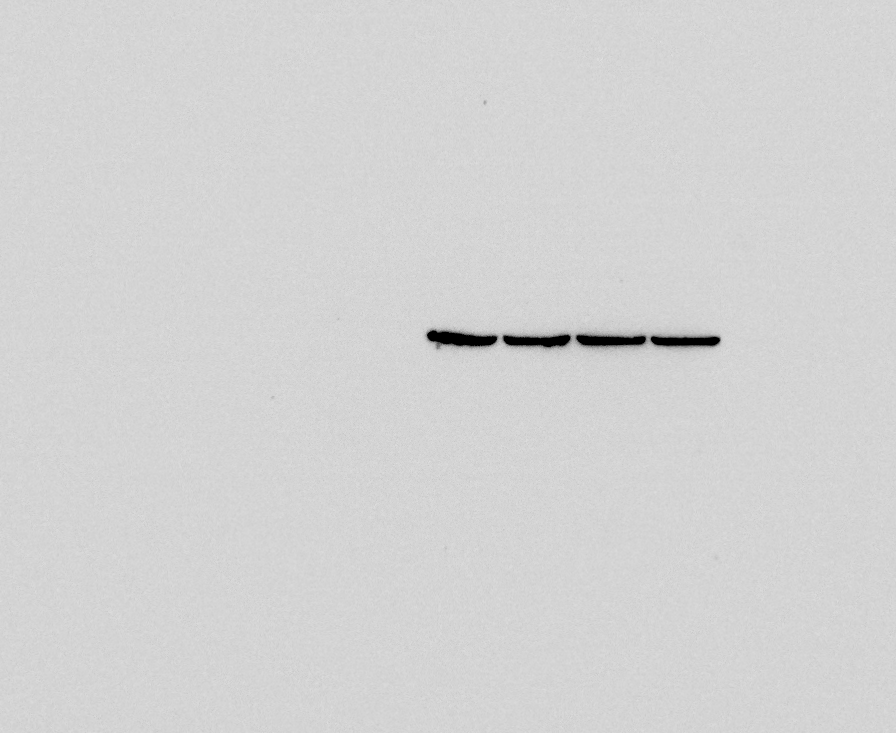


**Figure 3F**

iNOS


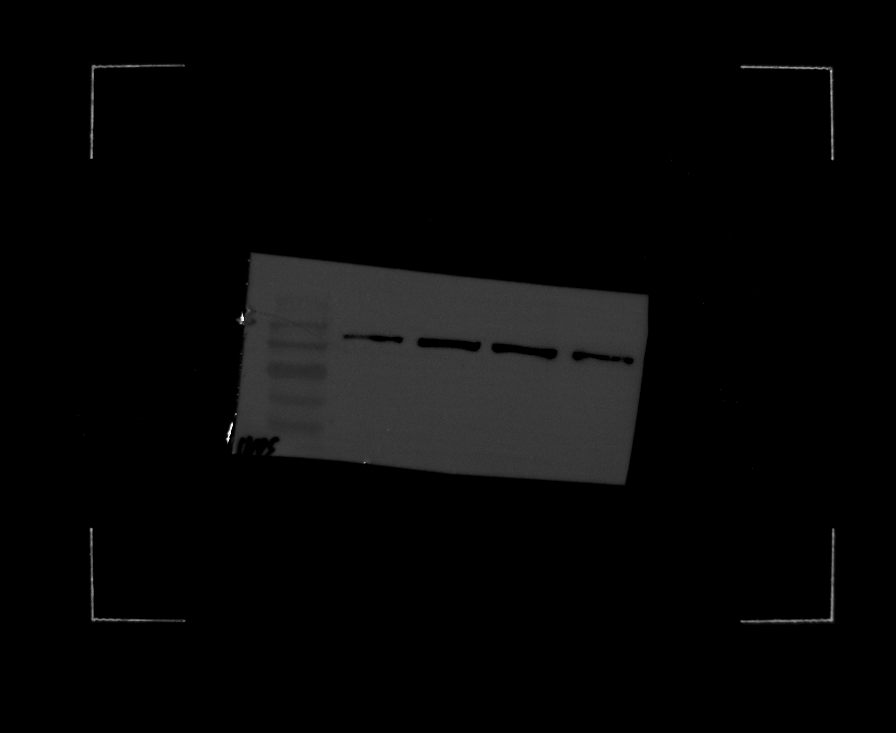

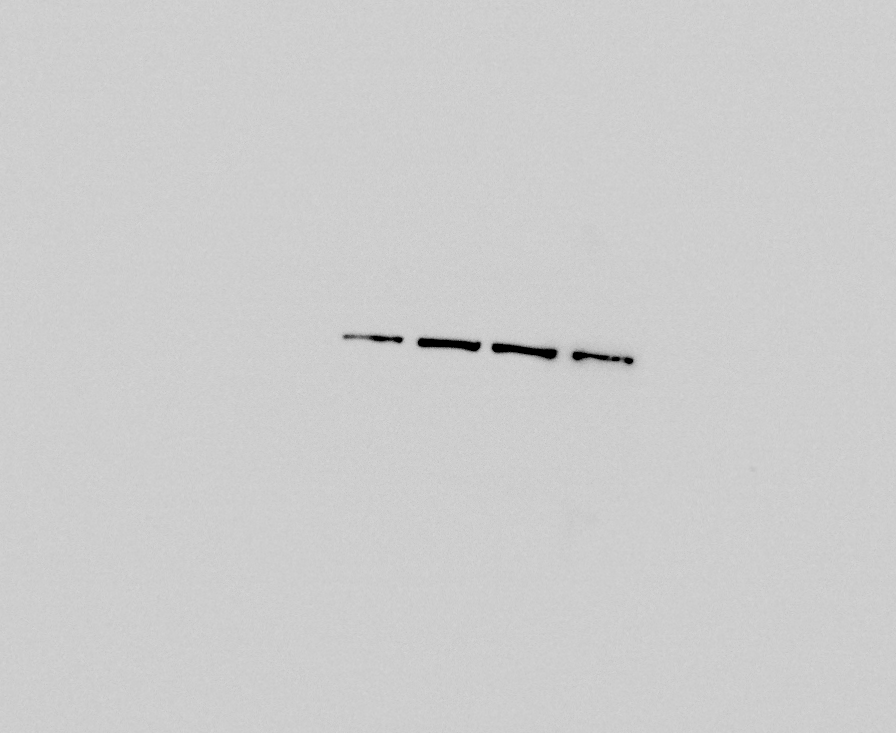


CD86


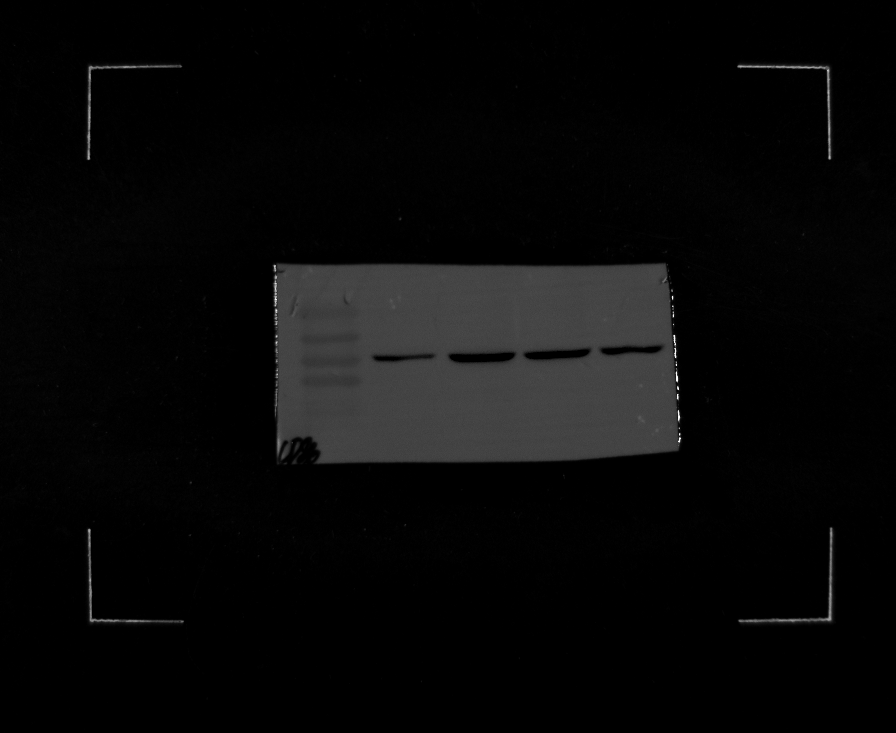

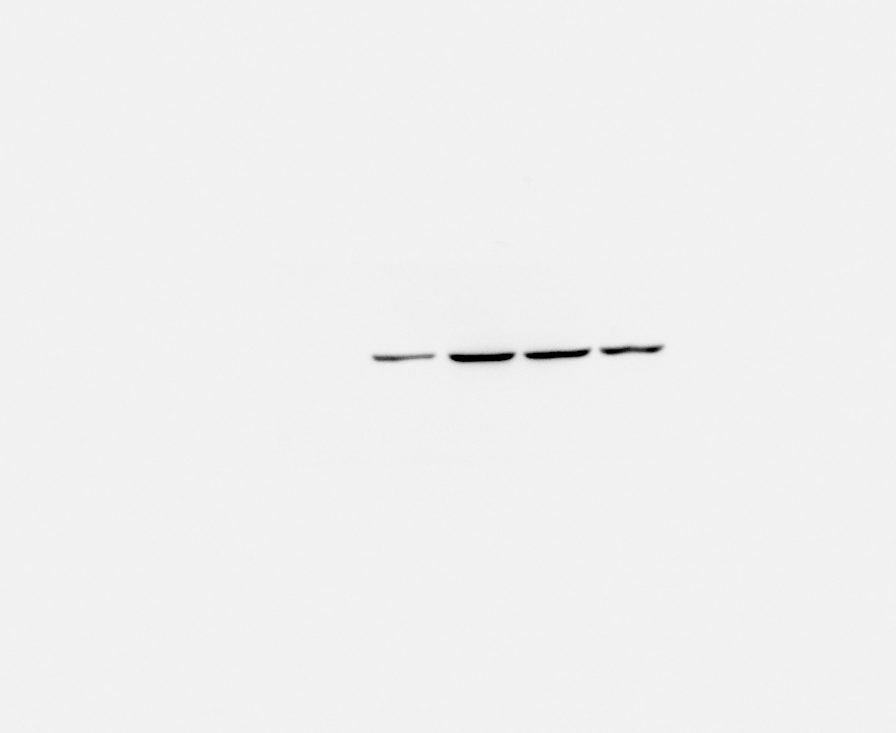


Arg-1


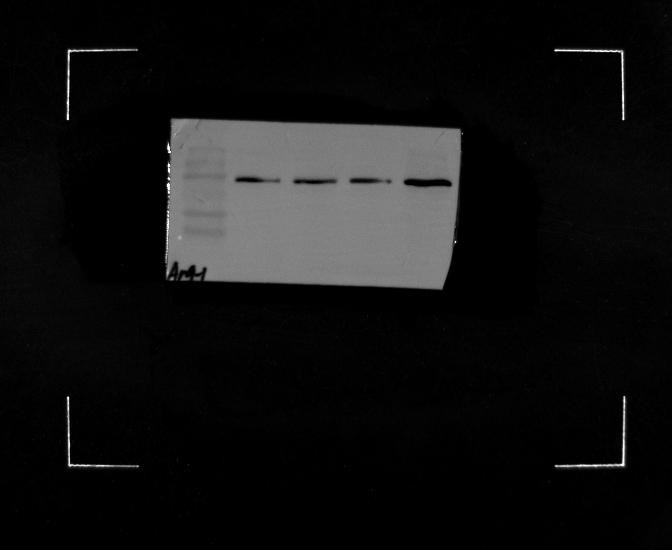

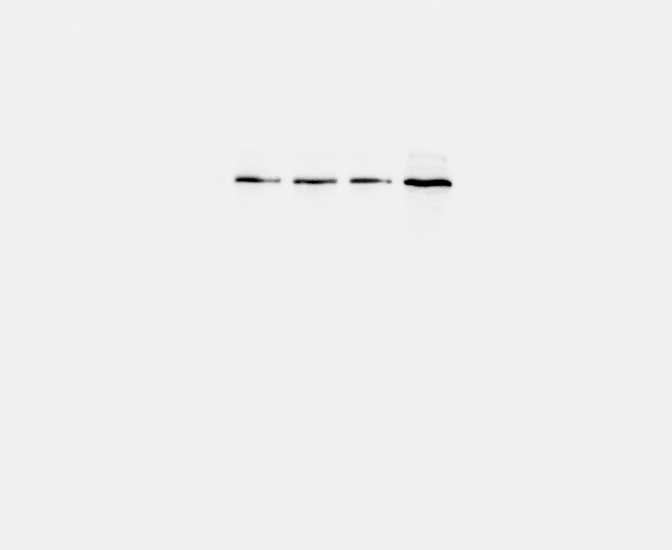


Fizz1


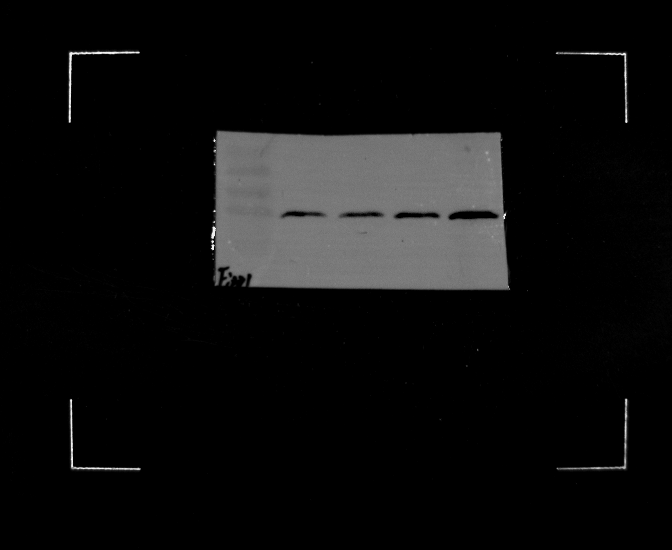

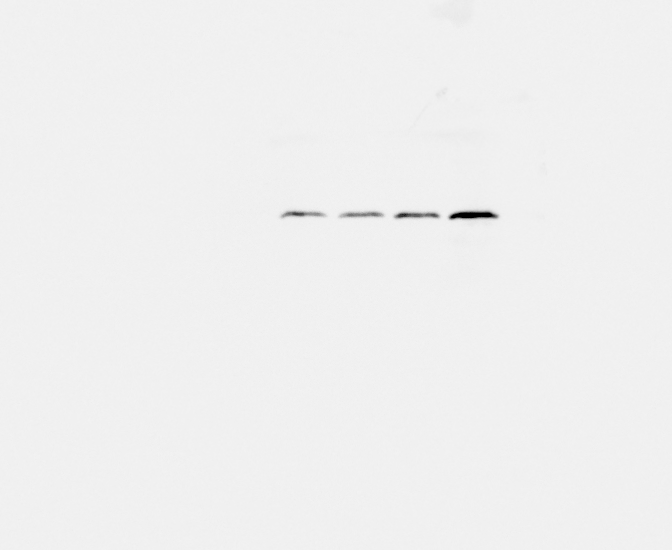


β-actin


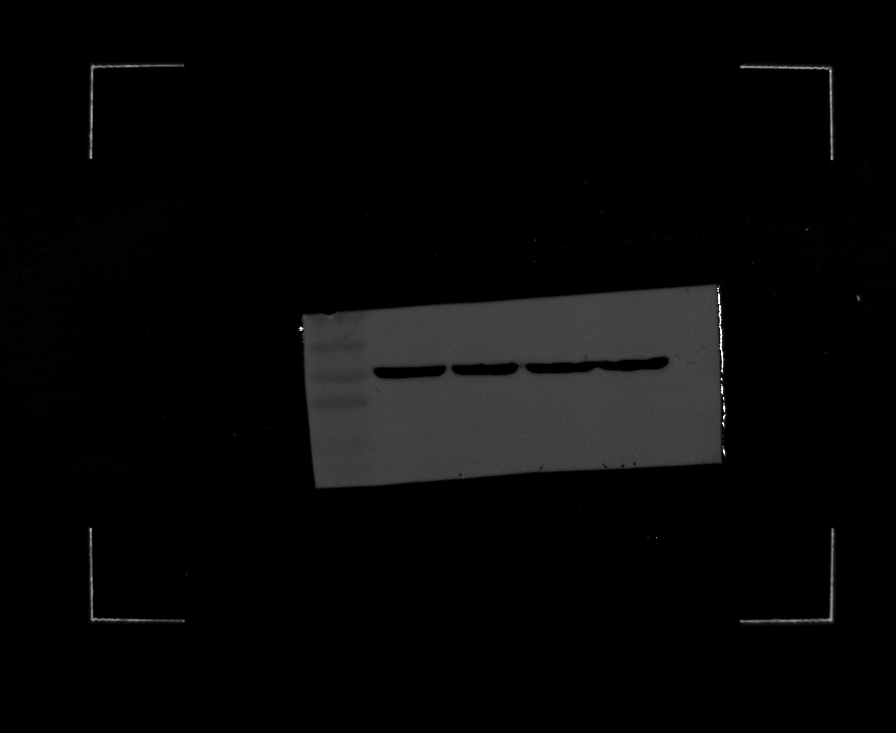

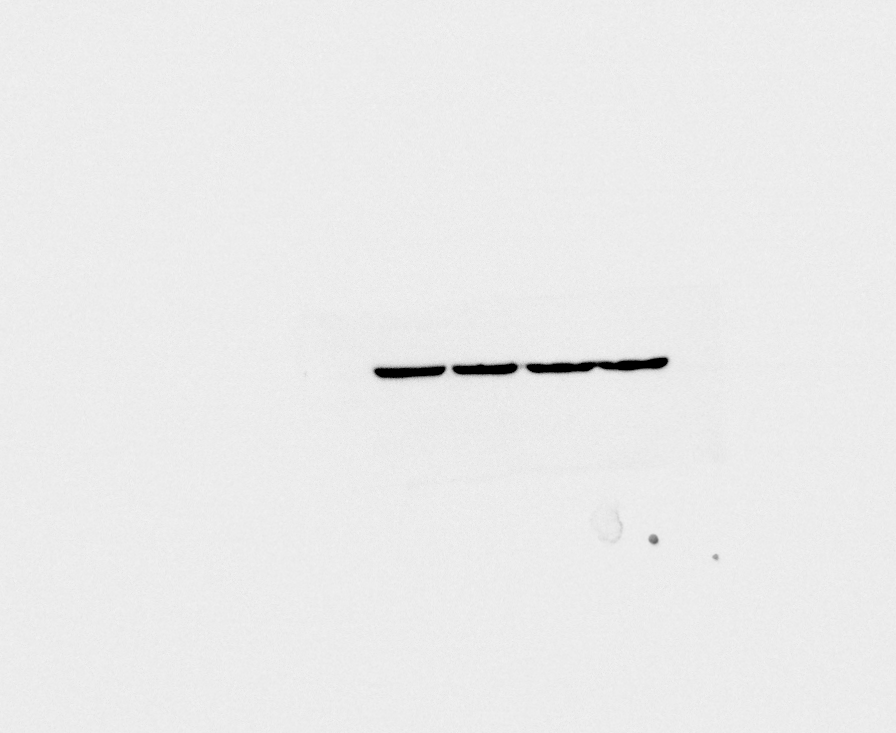


**Figure 4B**

USP7


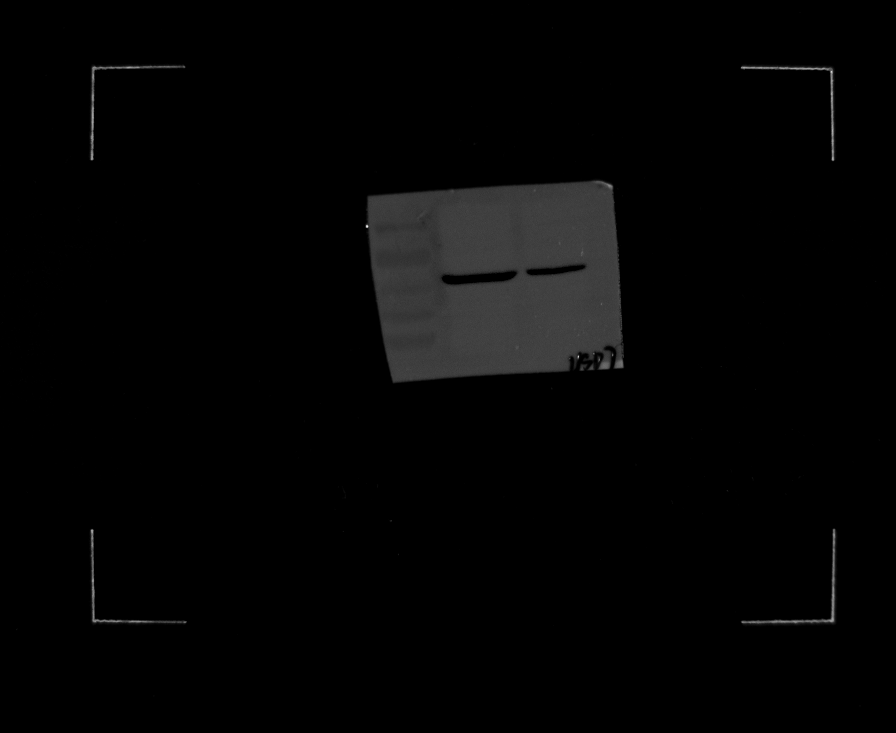

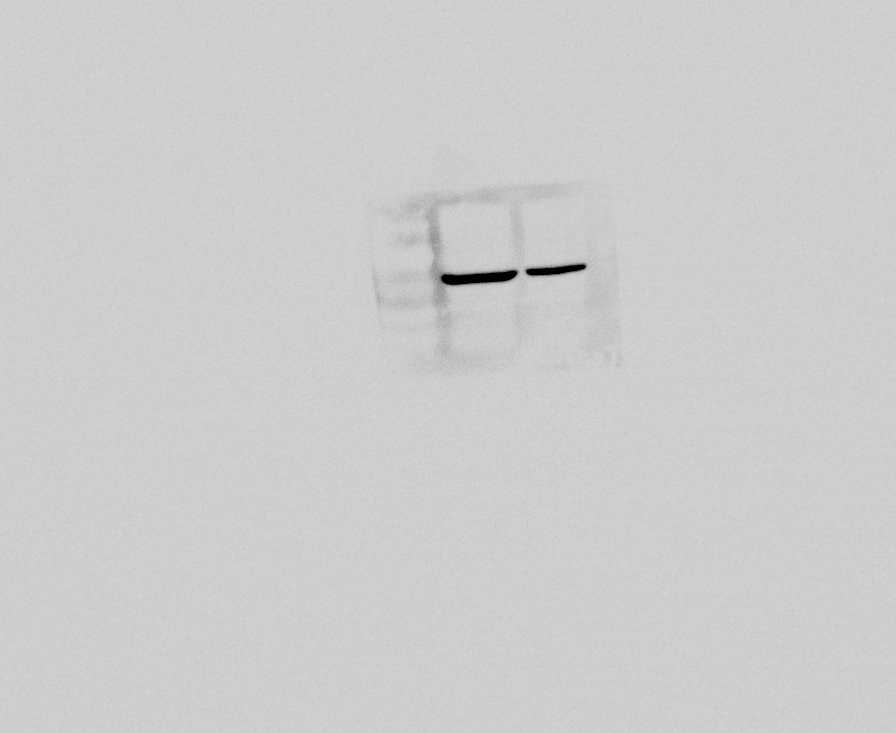


β-actin


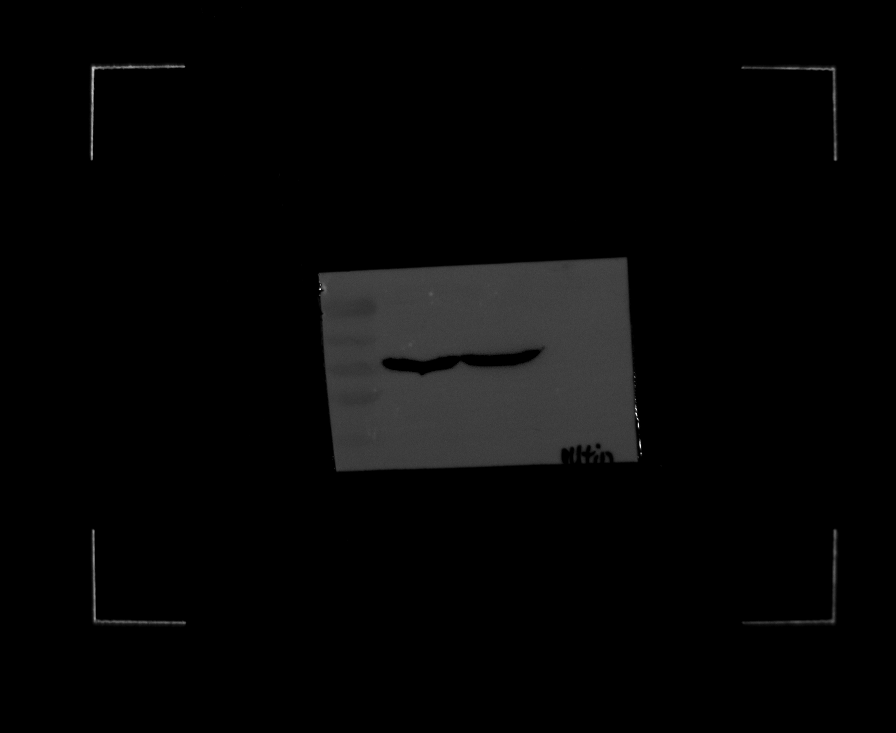

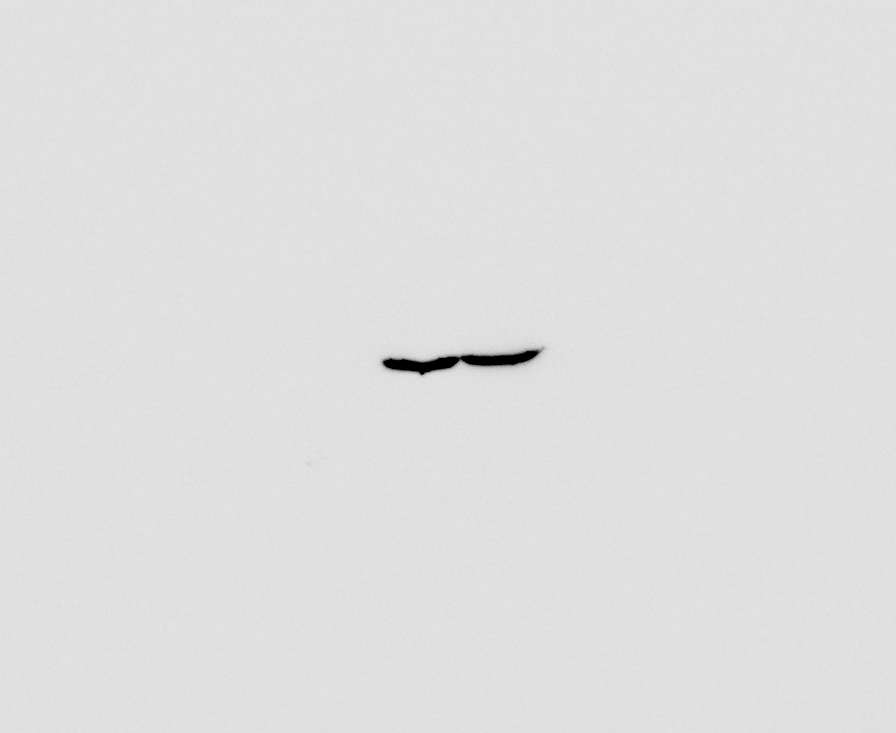


**Figure 4D**

IL-1β


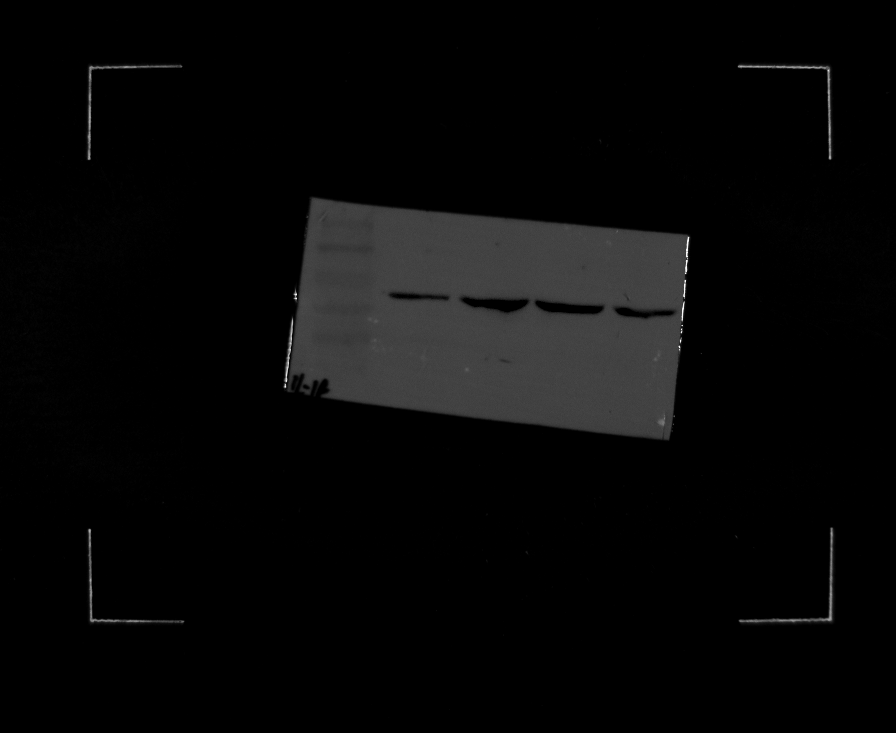

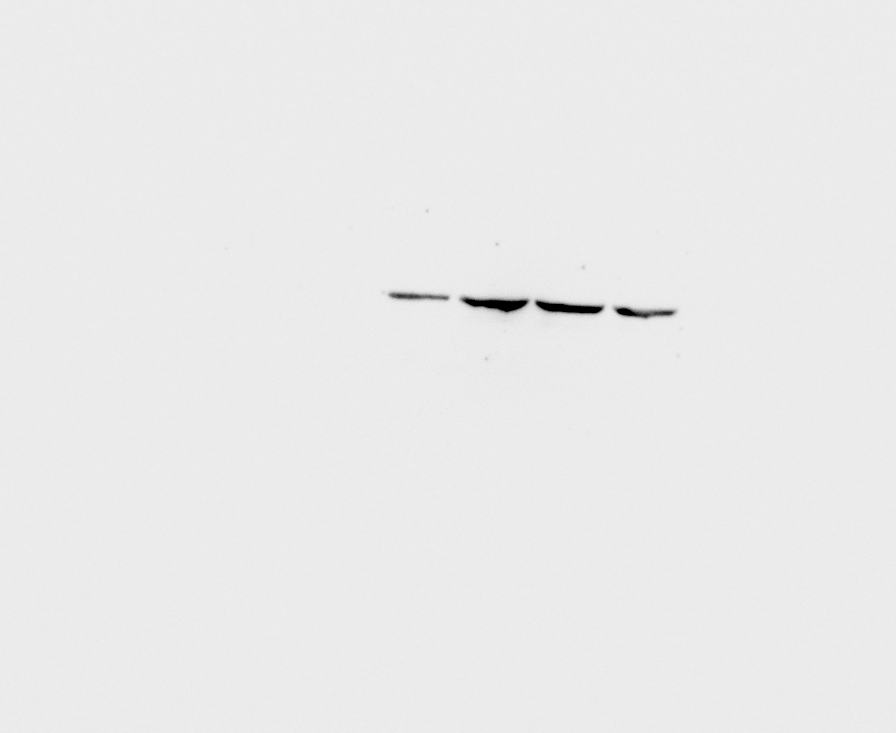


TNF-α


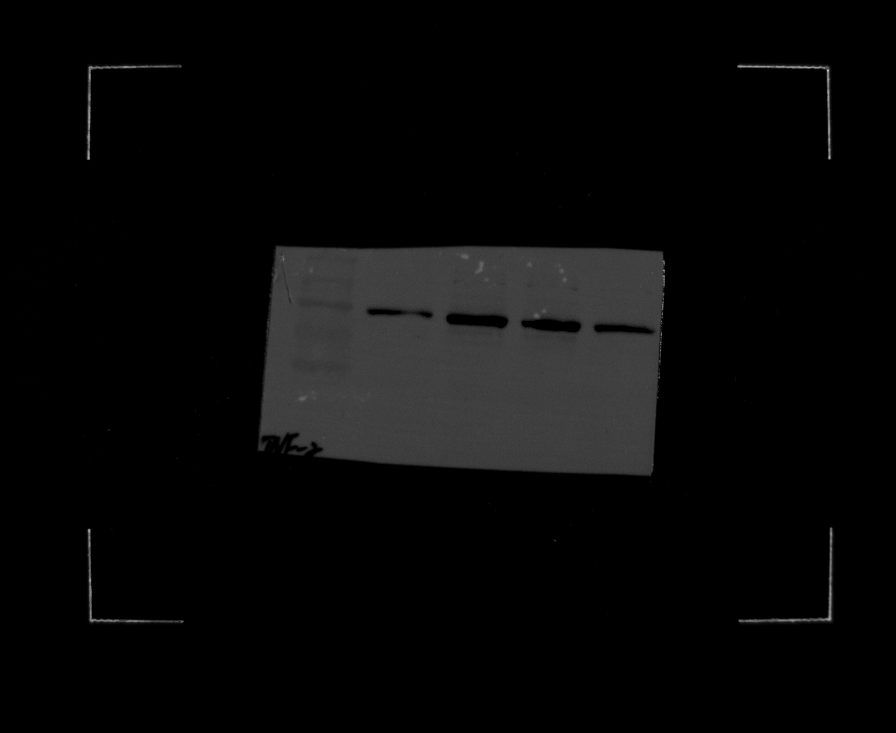

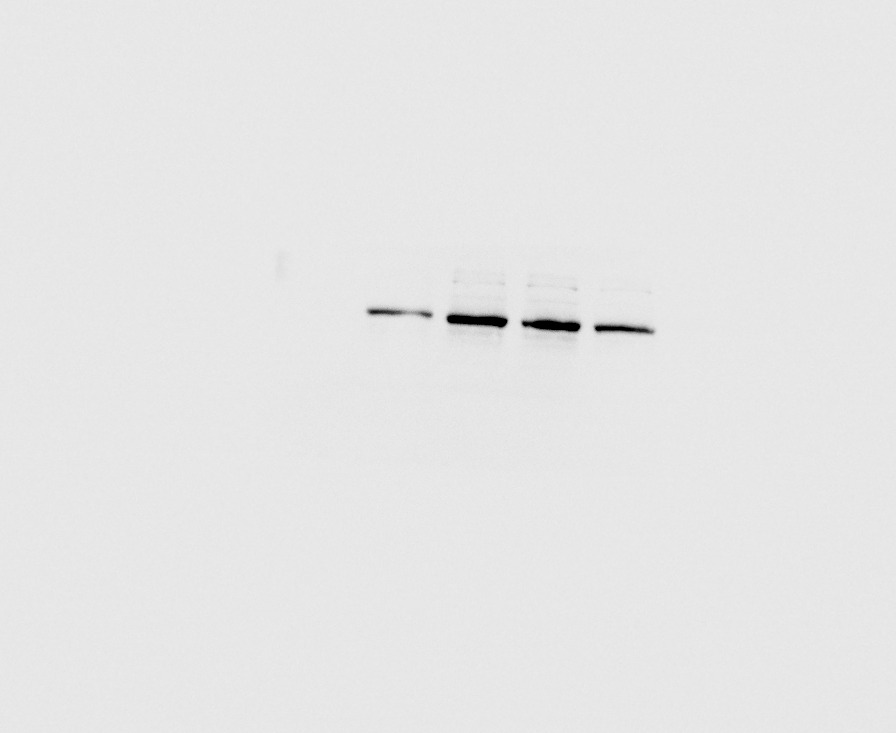


IL-6


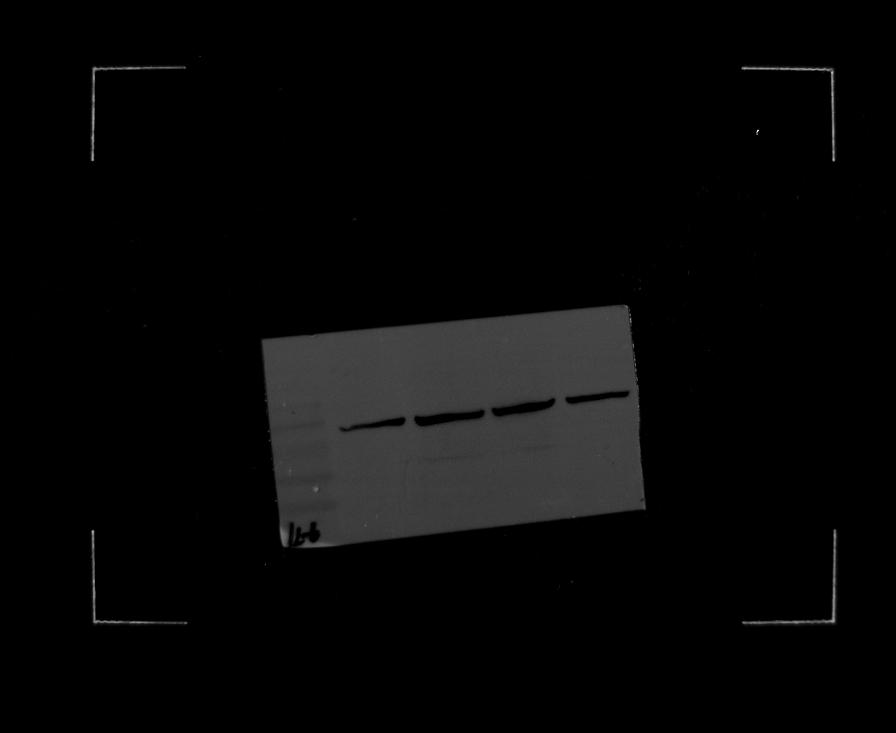

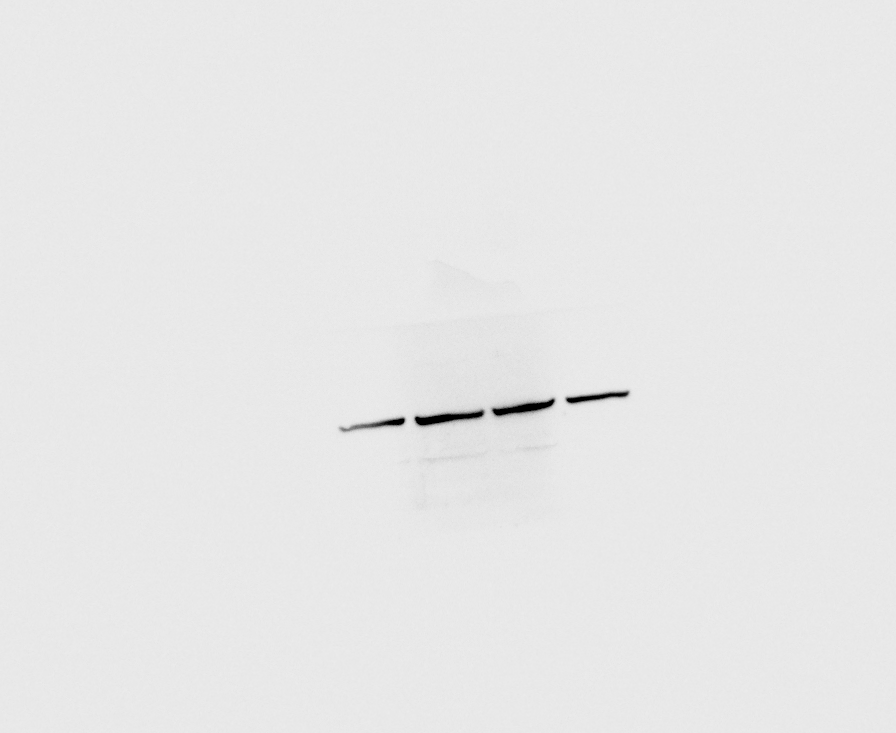


β-actin


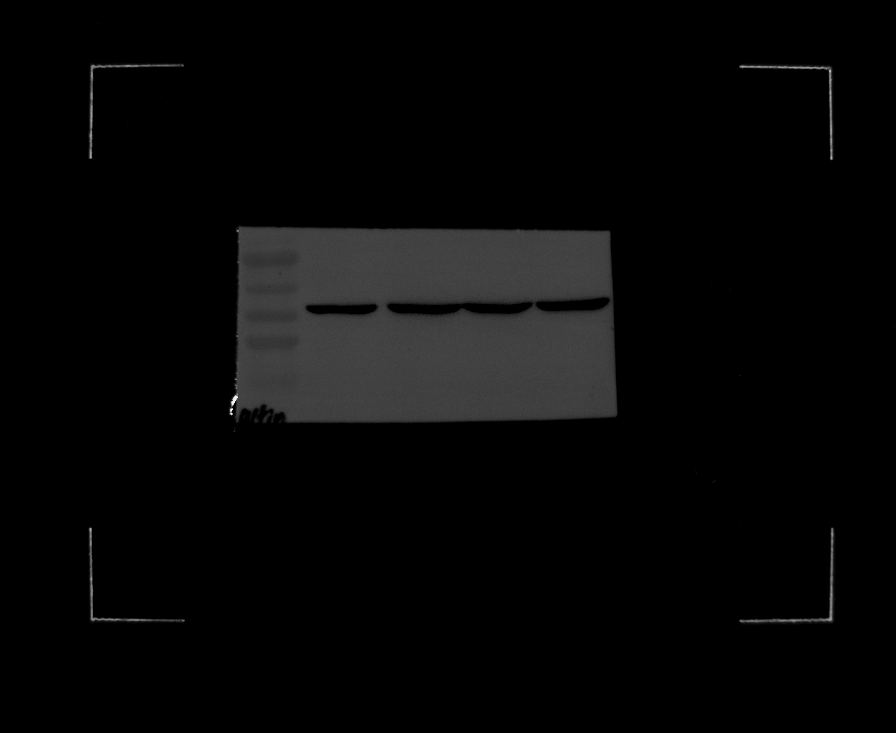

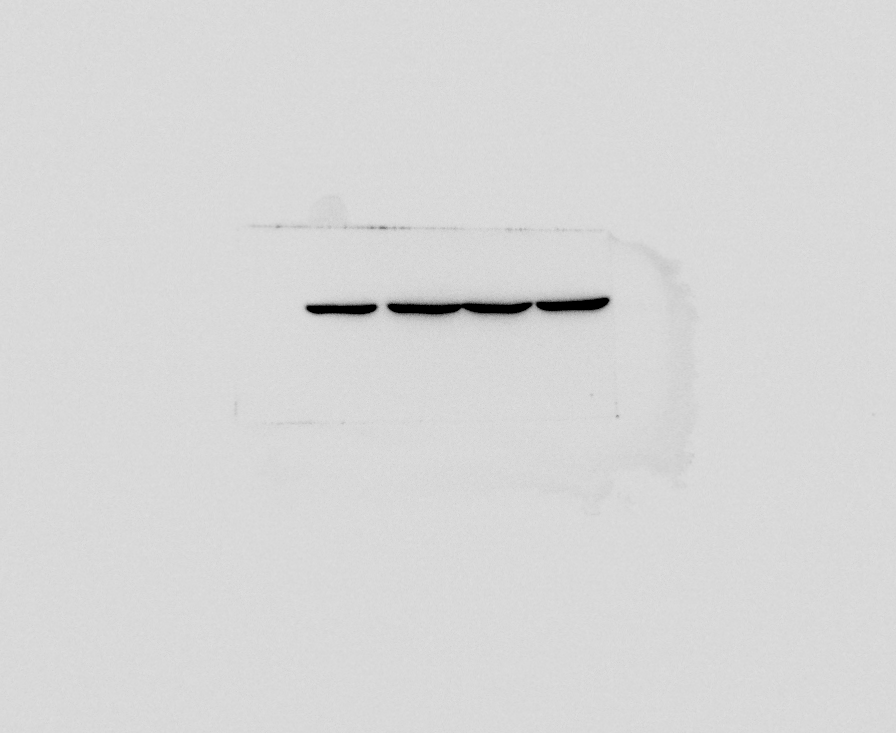


CD206


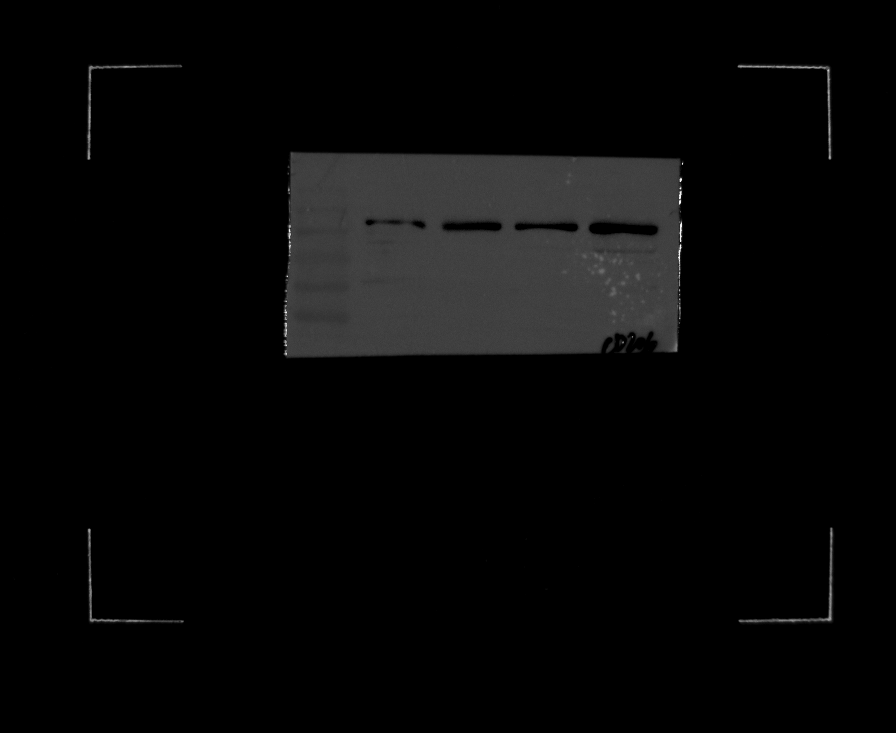

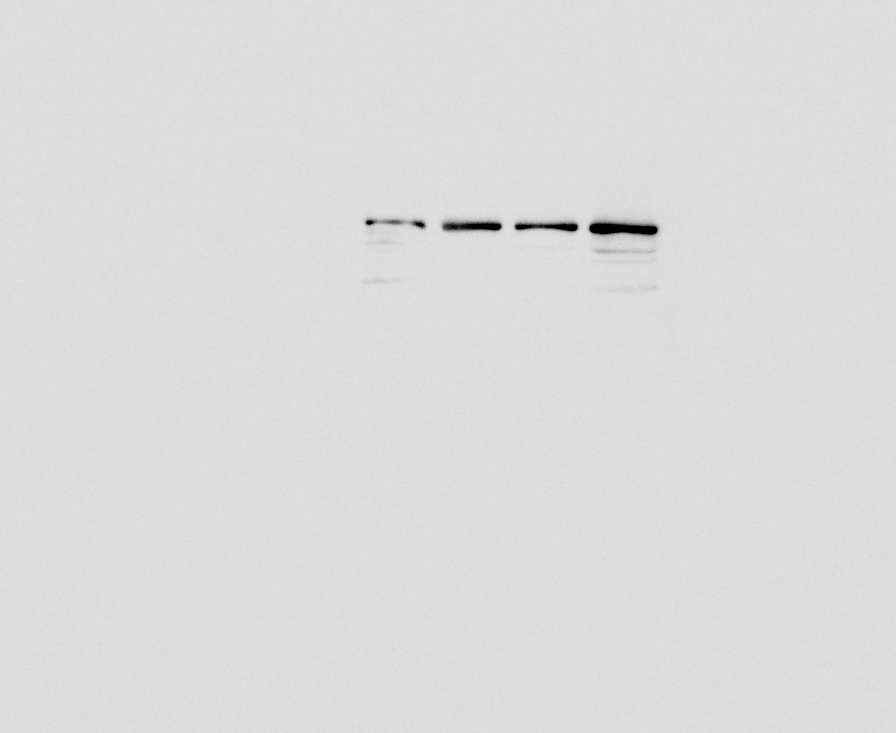


Fizz1


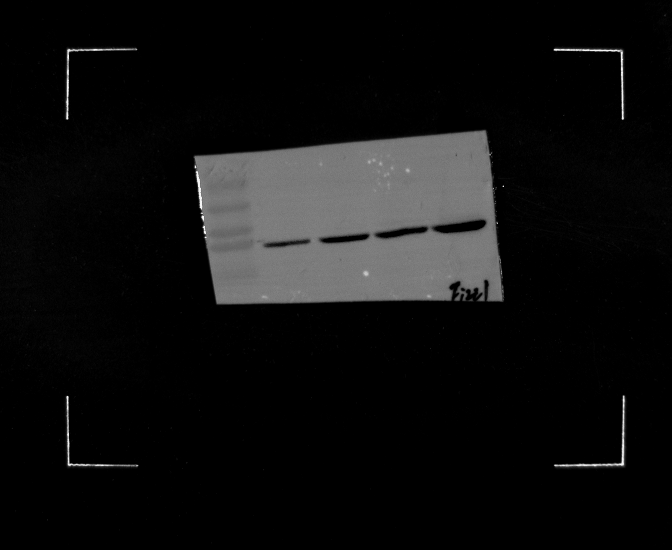

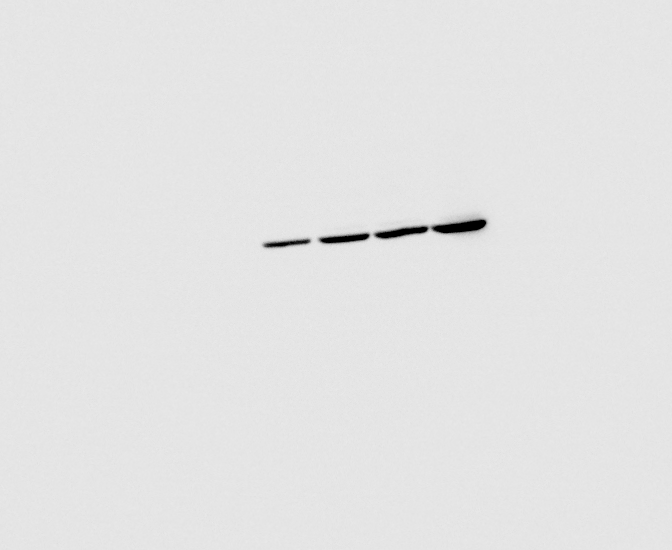


IL-10


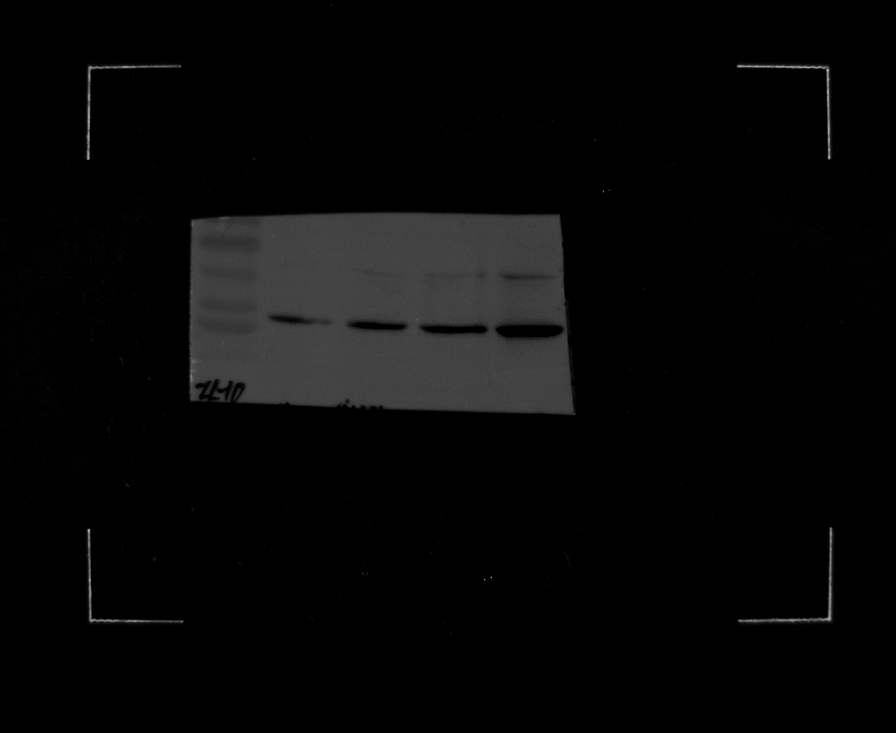

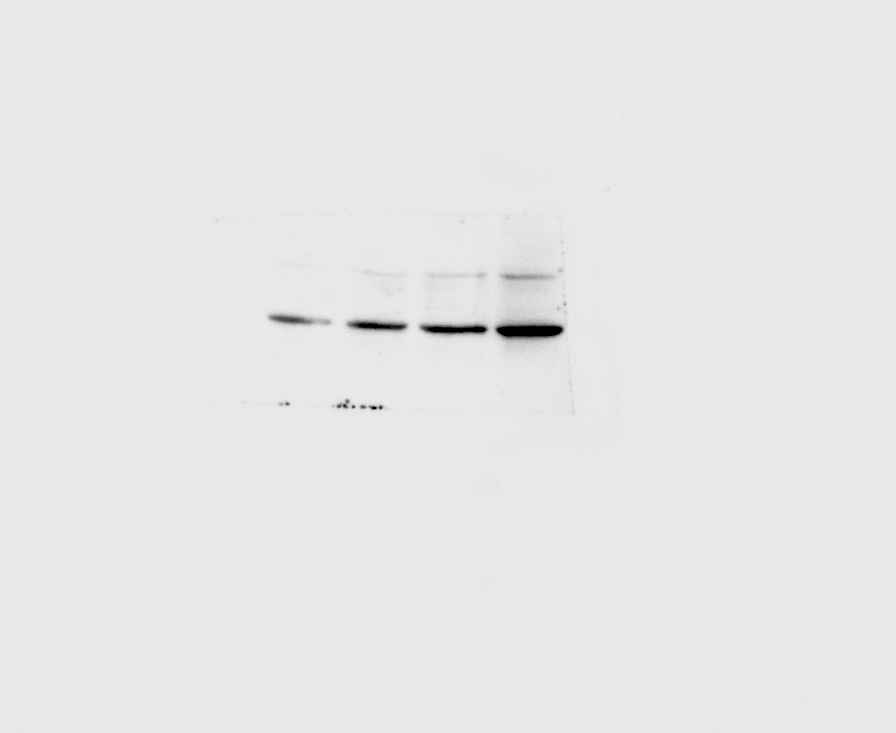


β-actin


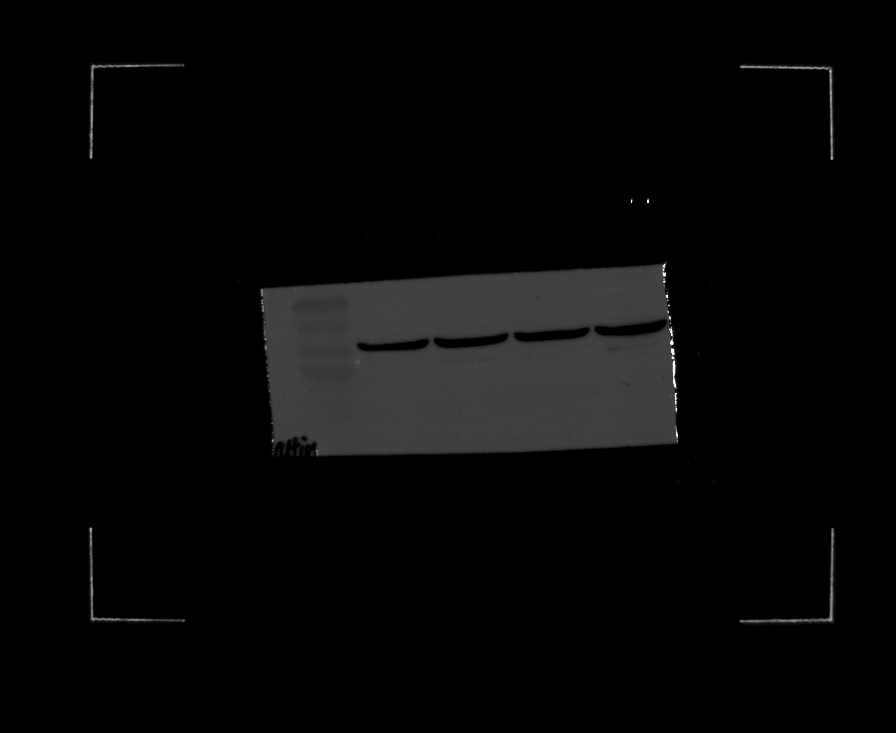

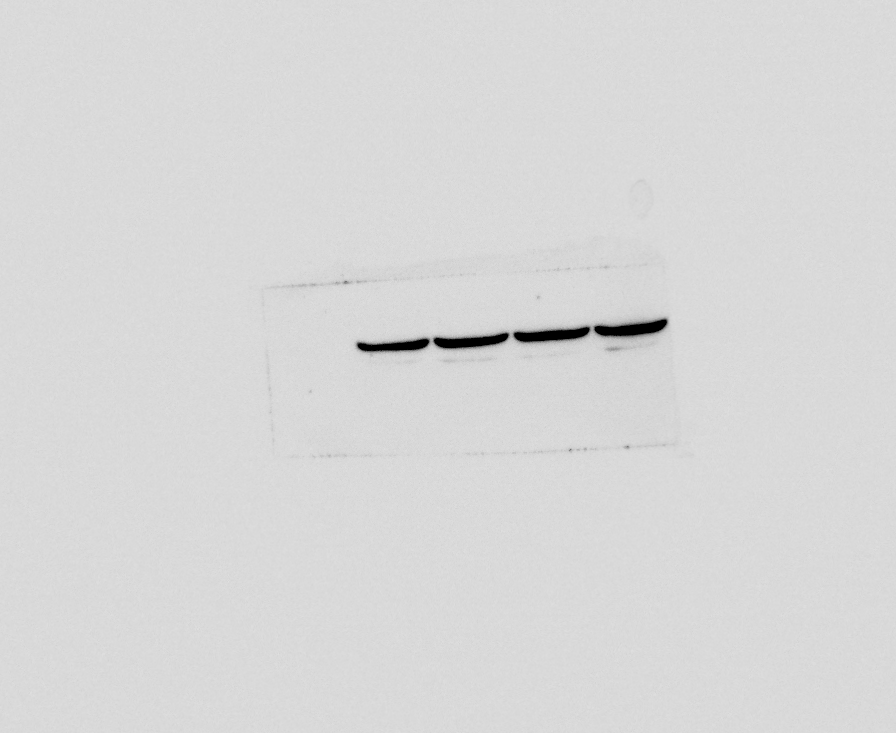


**Figure 5F**

GLUT1


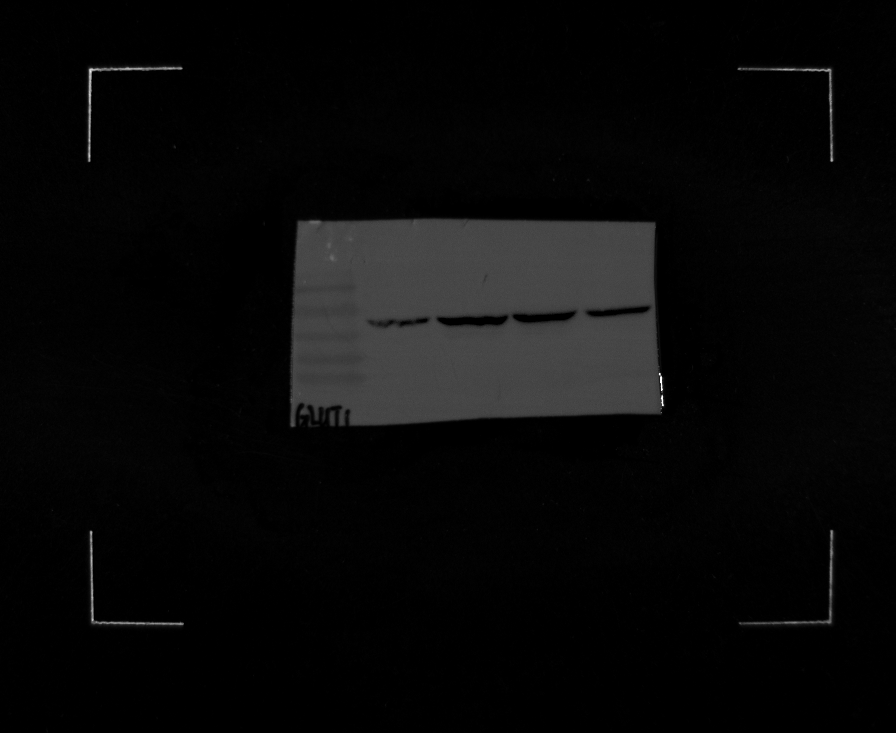

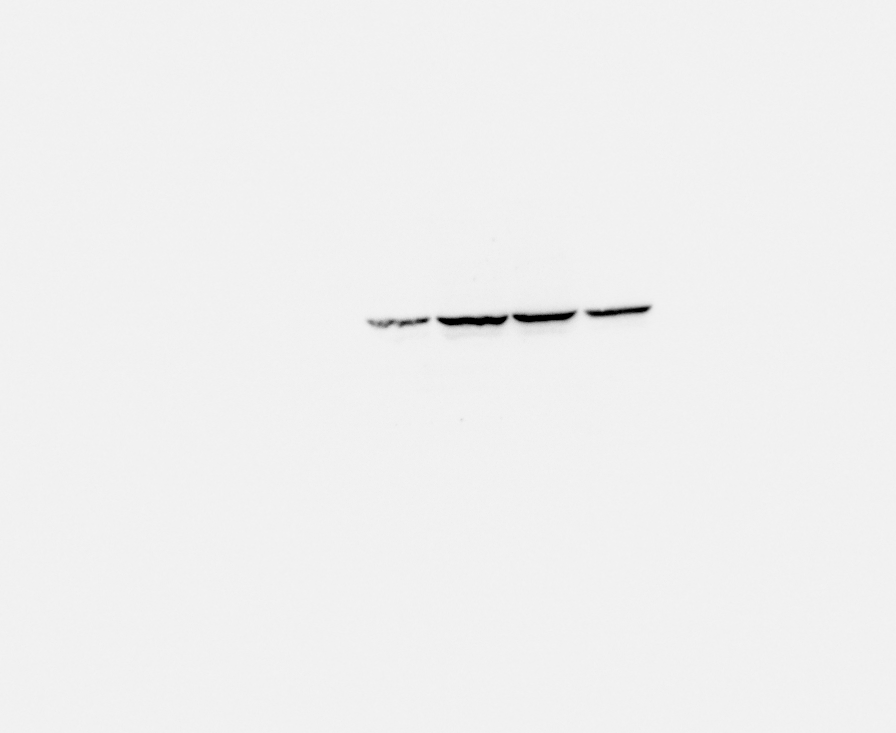


HK-2


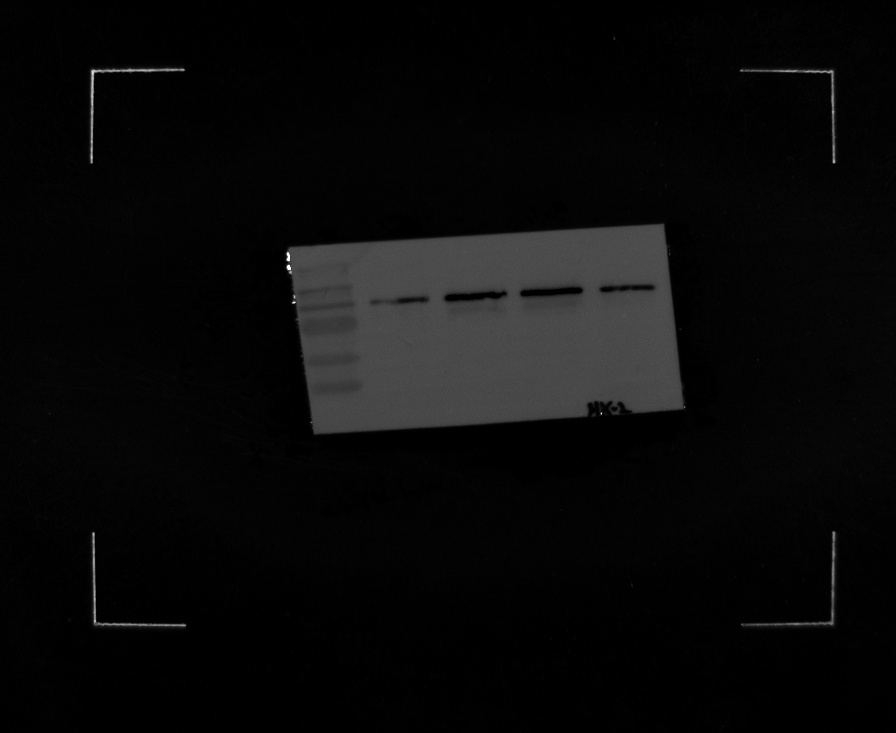

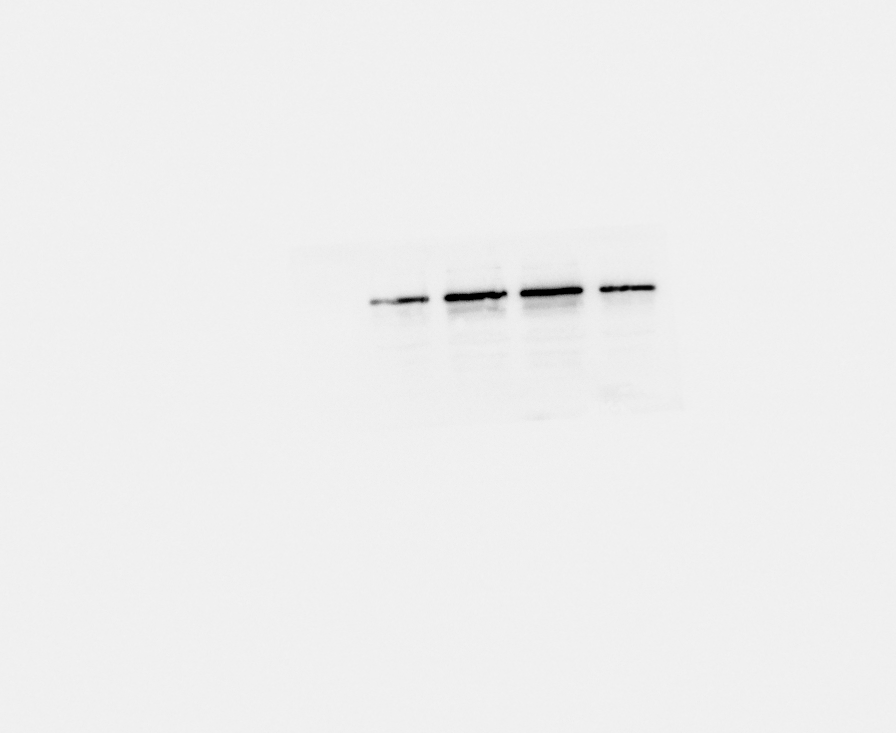


PKM2


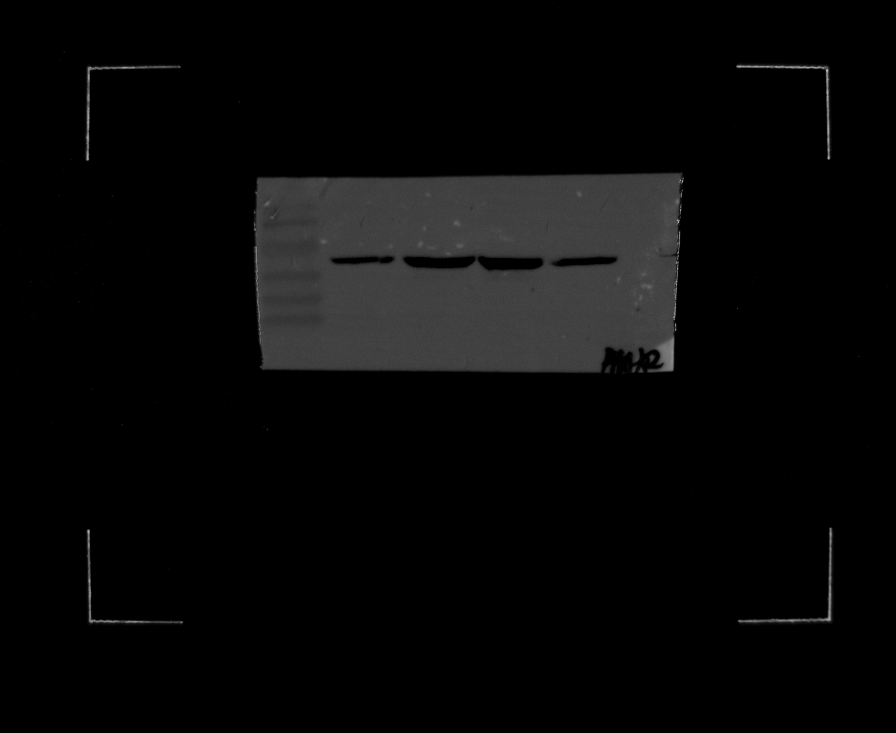

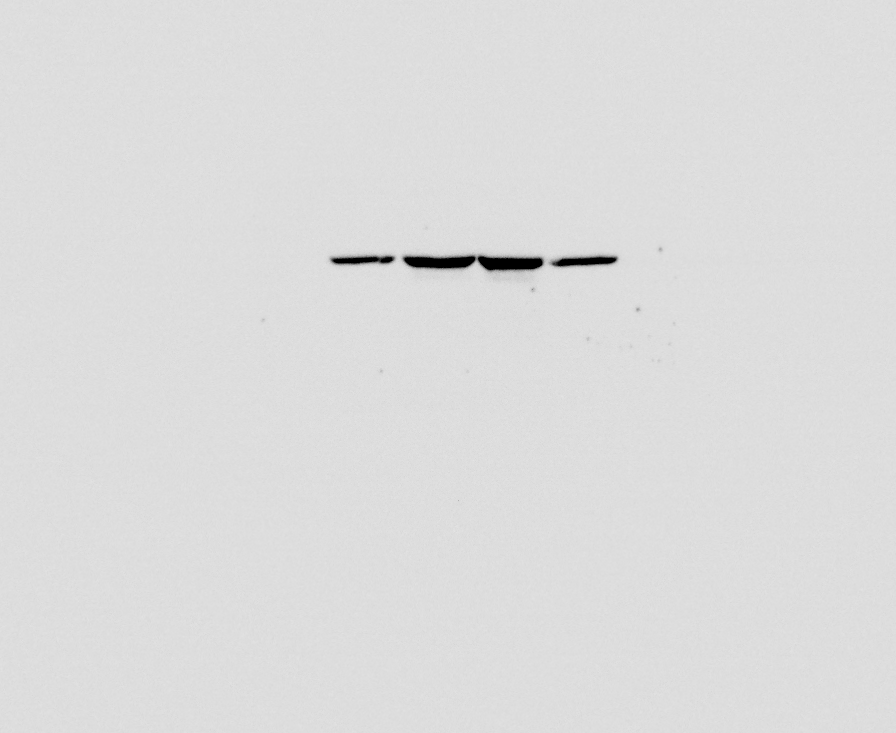


LDHA


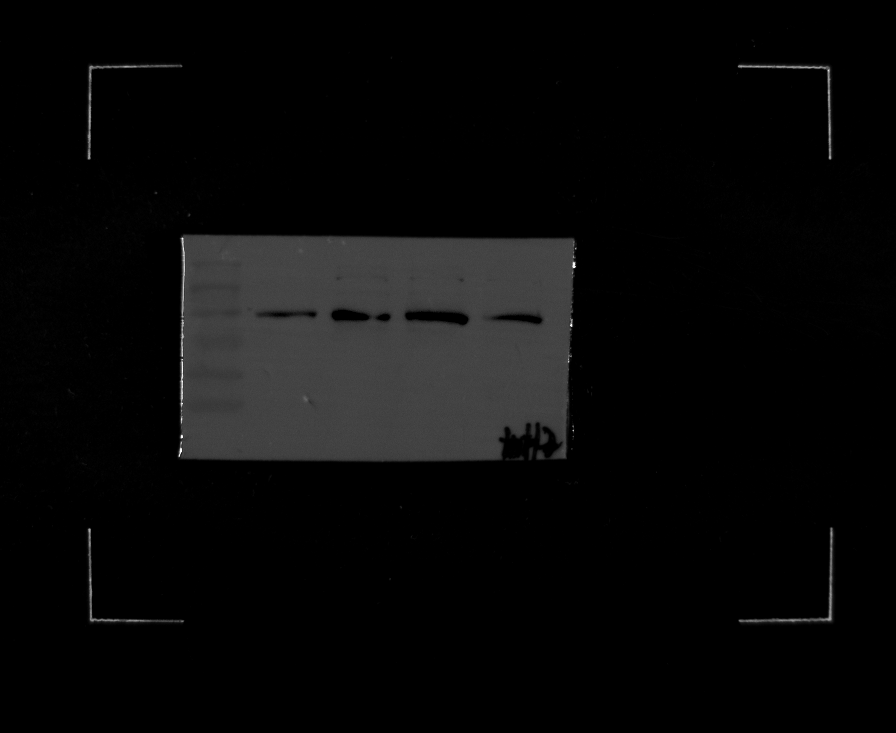

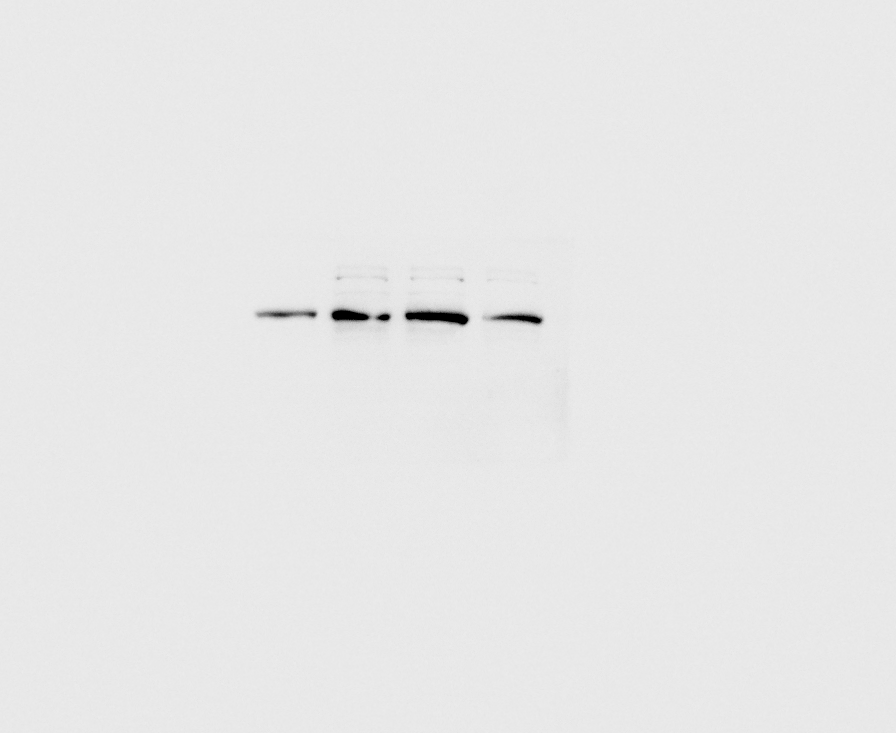


β-actin


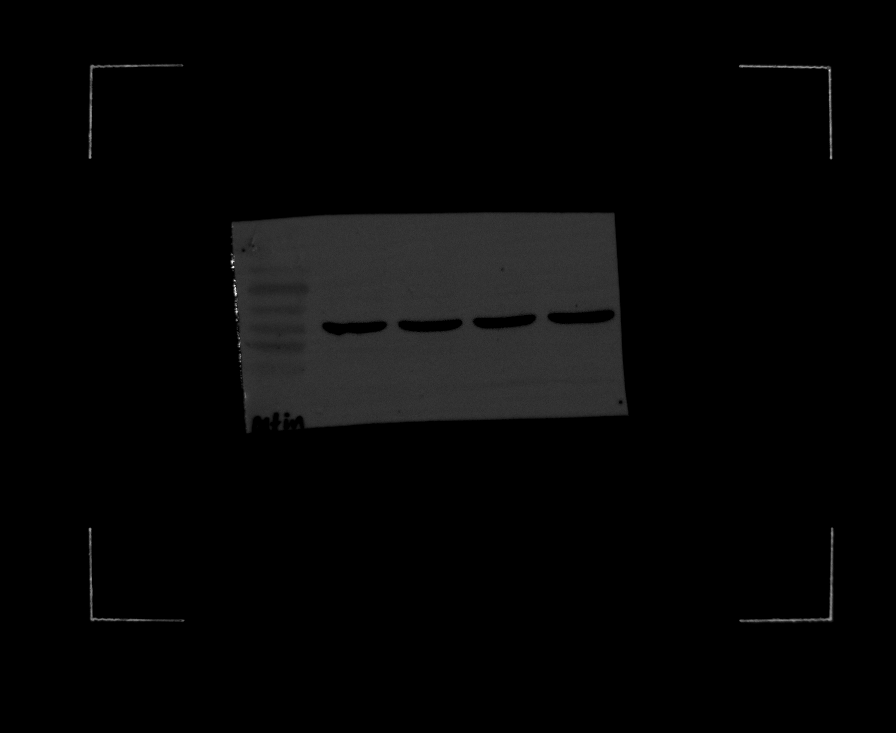

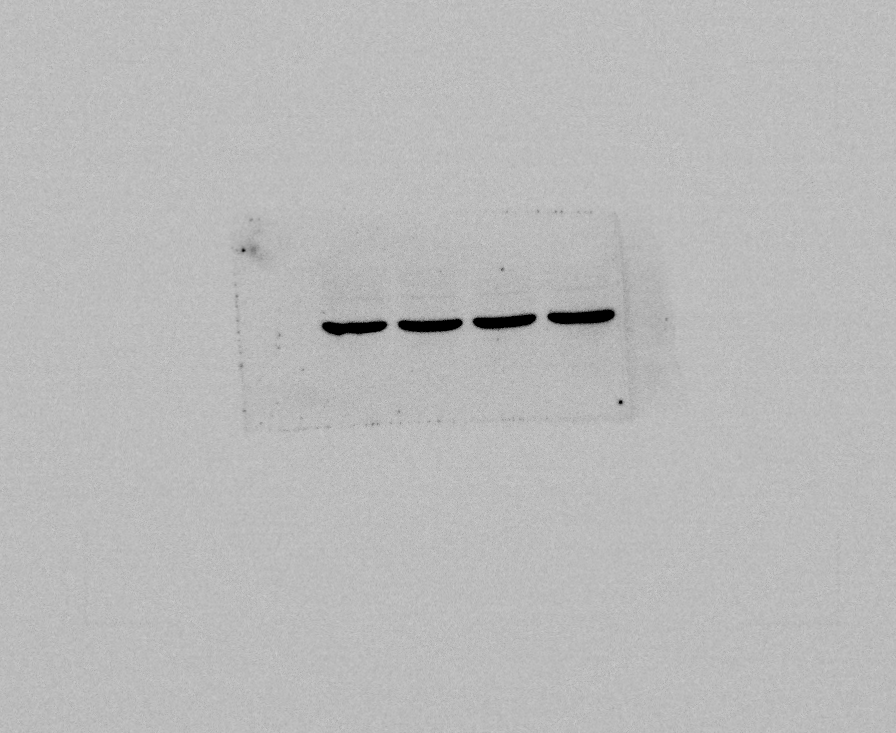


**Figure 6A**

IP-USP7

PKM2


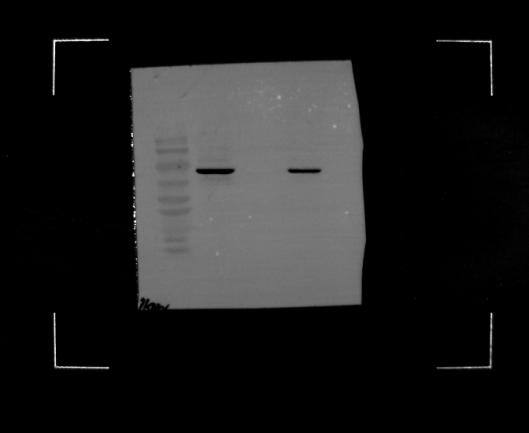

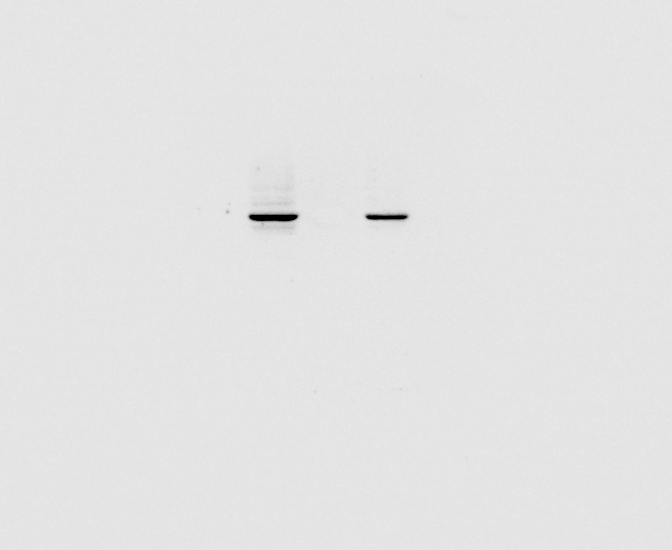


IP-USP7

USP7


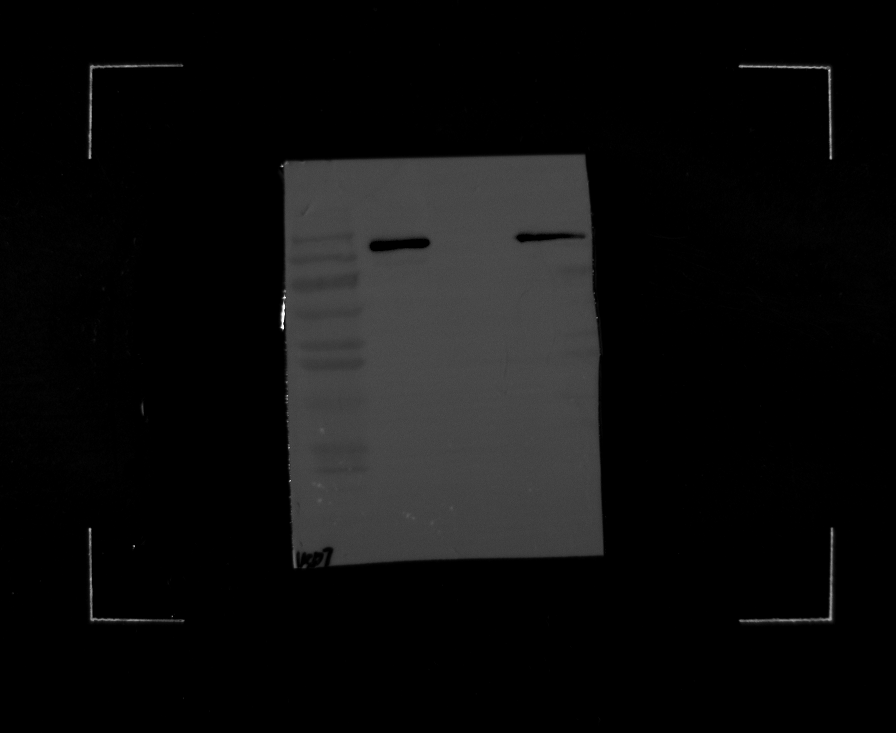

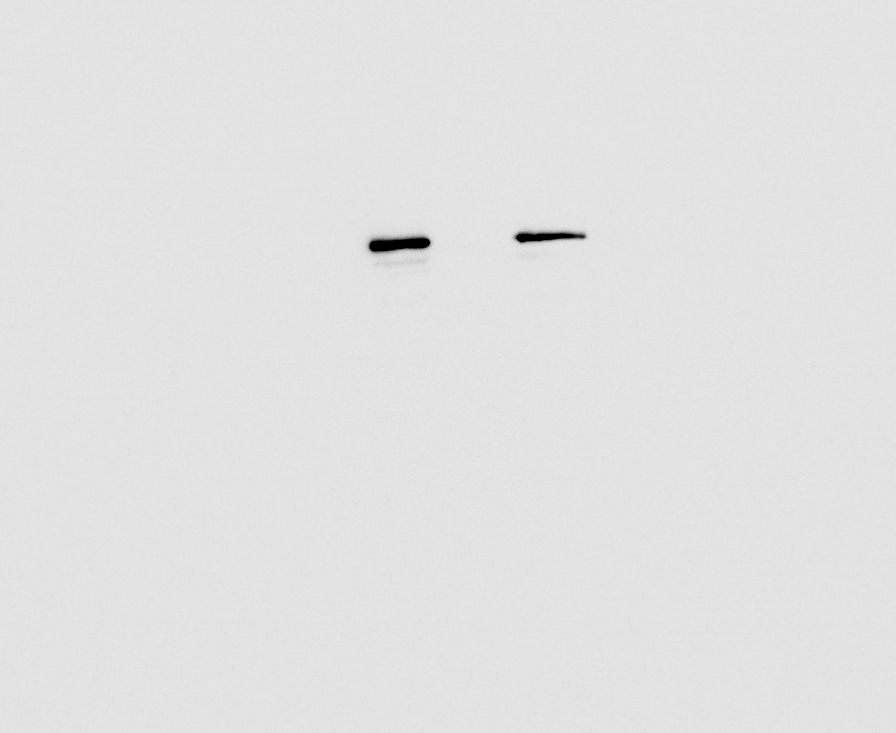


MCL

PKM2


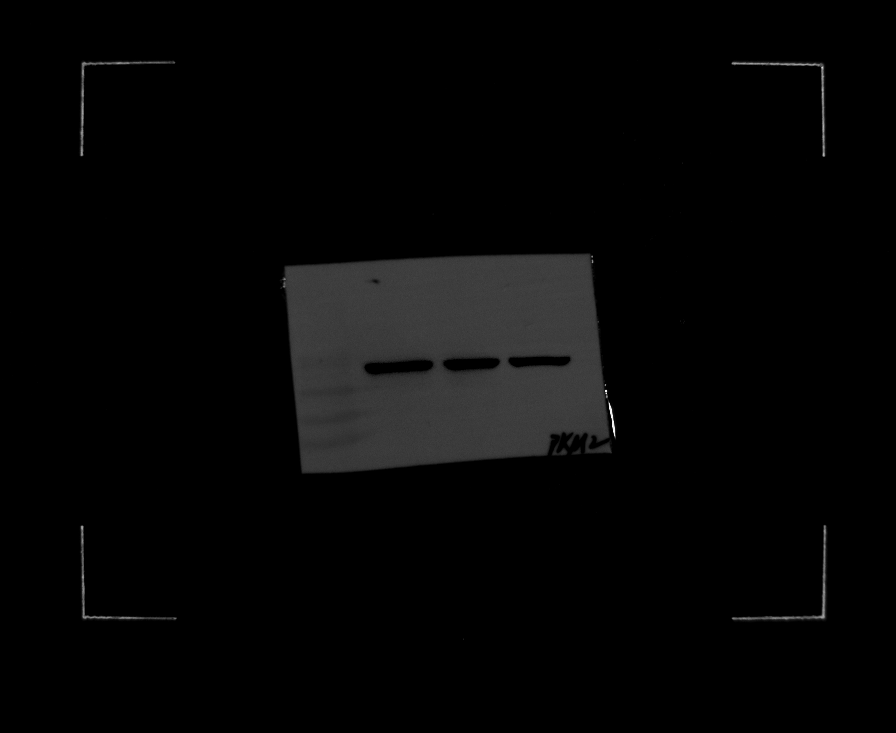

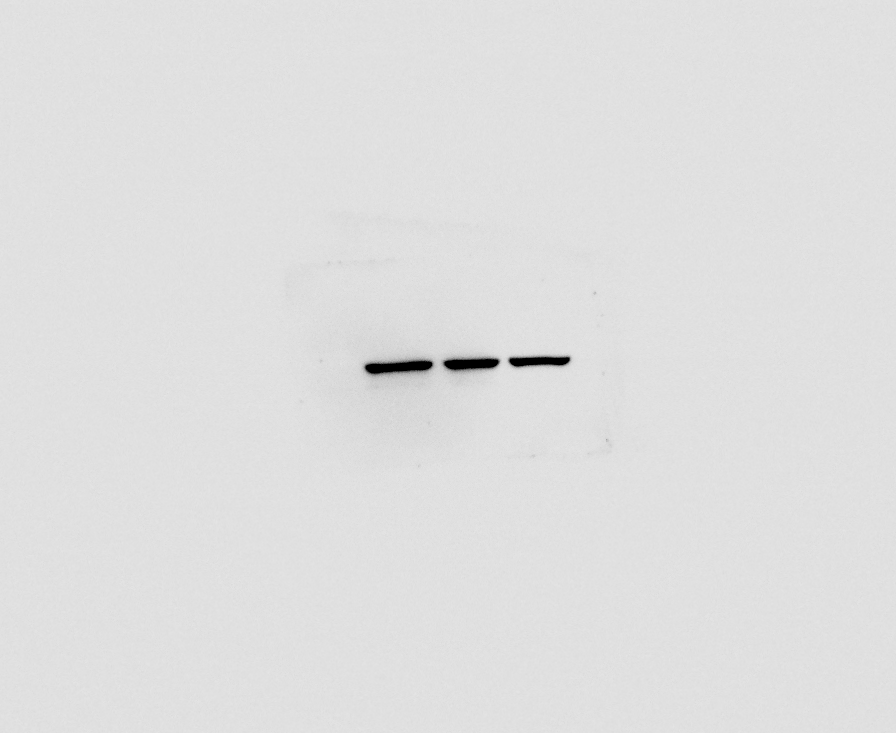


USP7


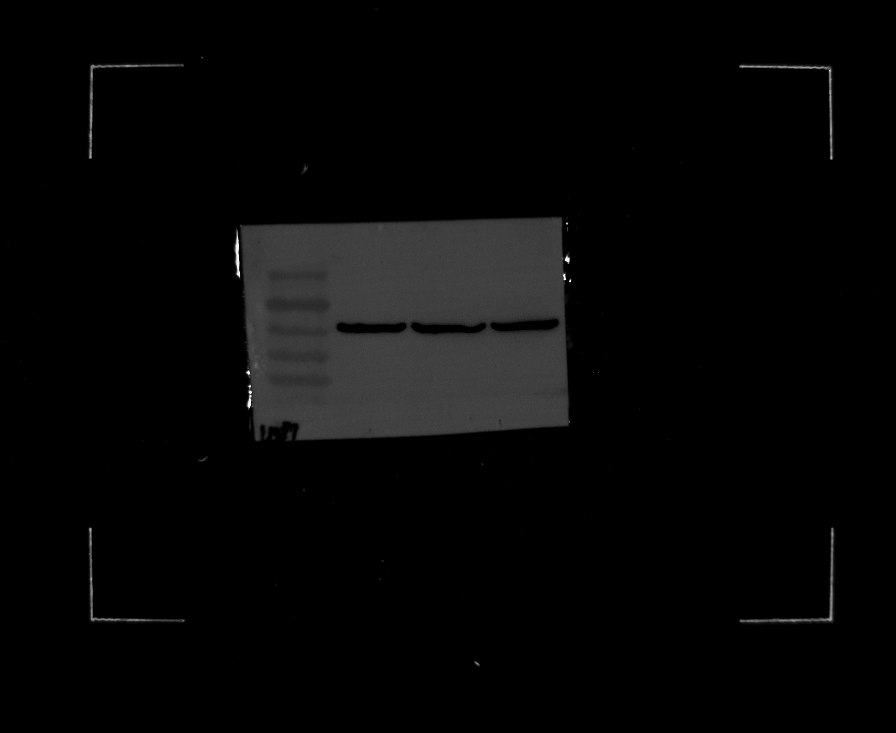

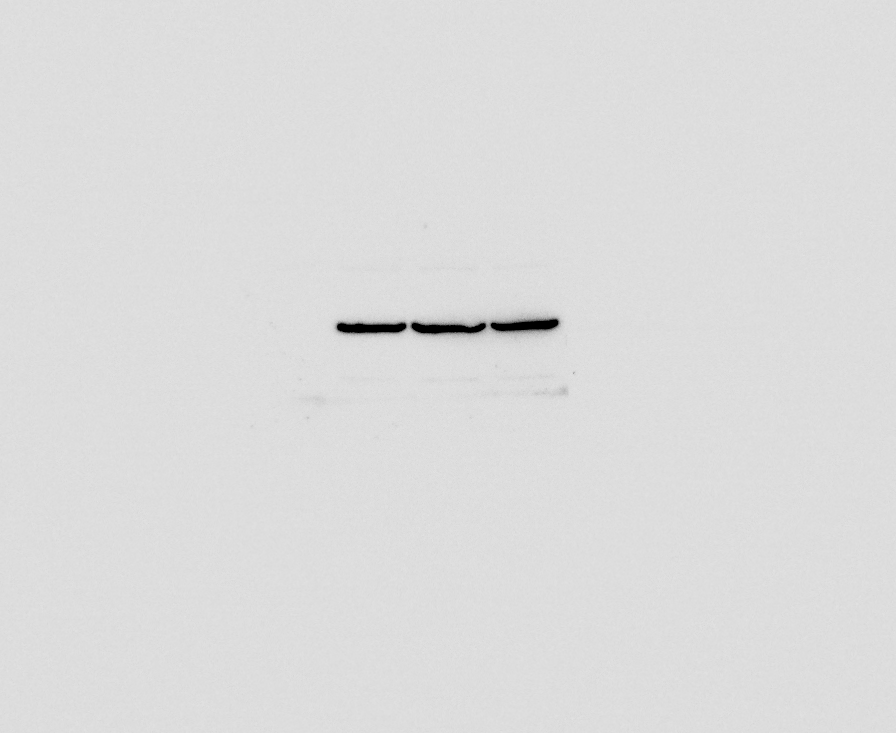


β-actin


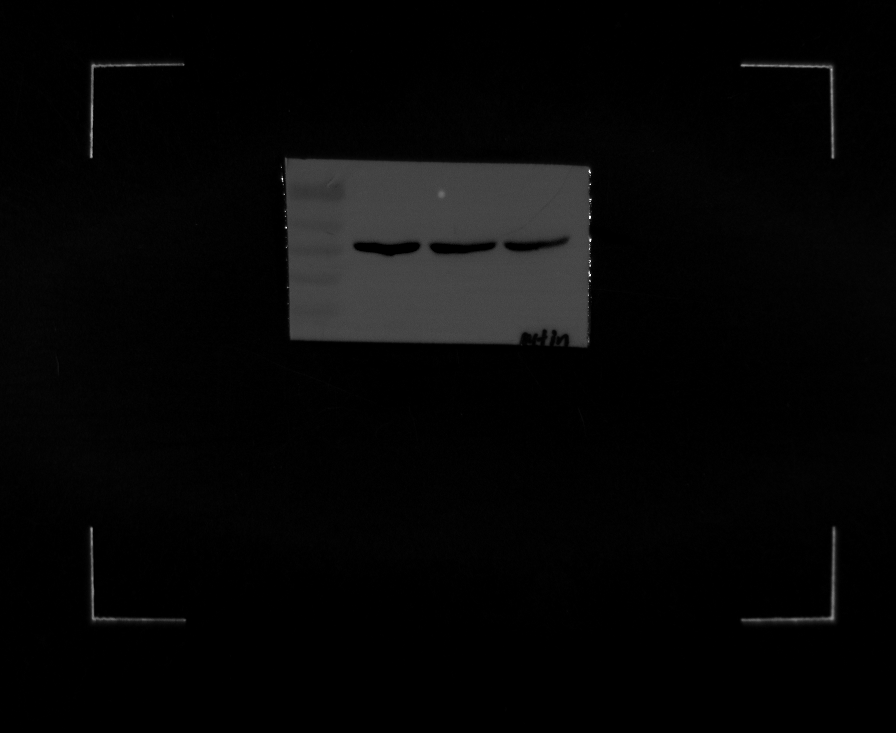

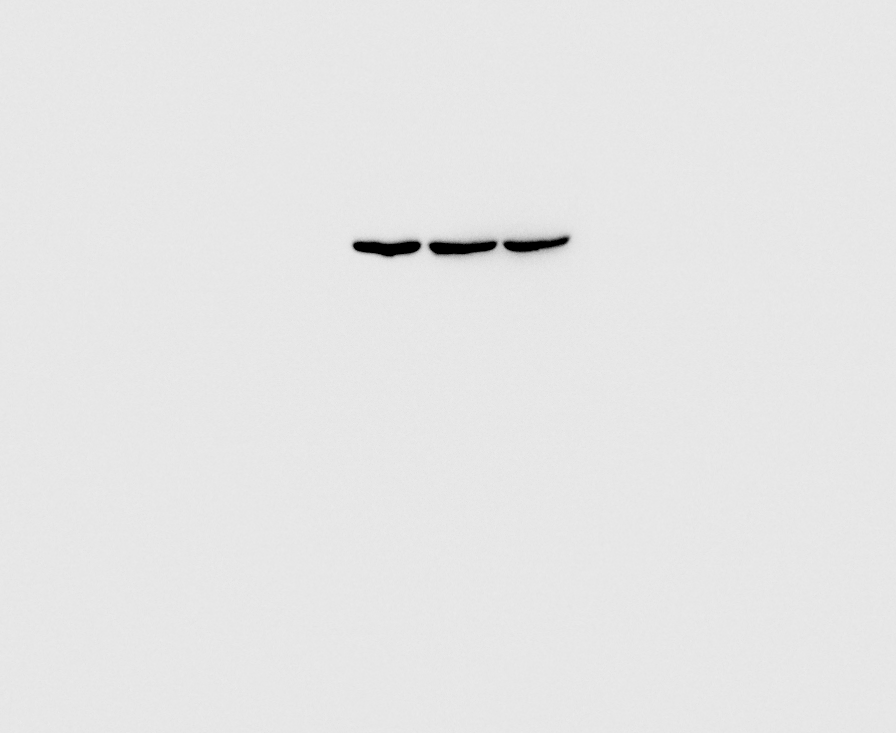


IP-PKM2

PKM2


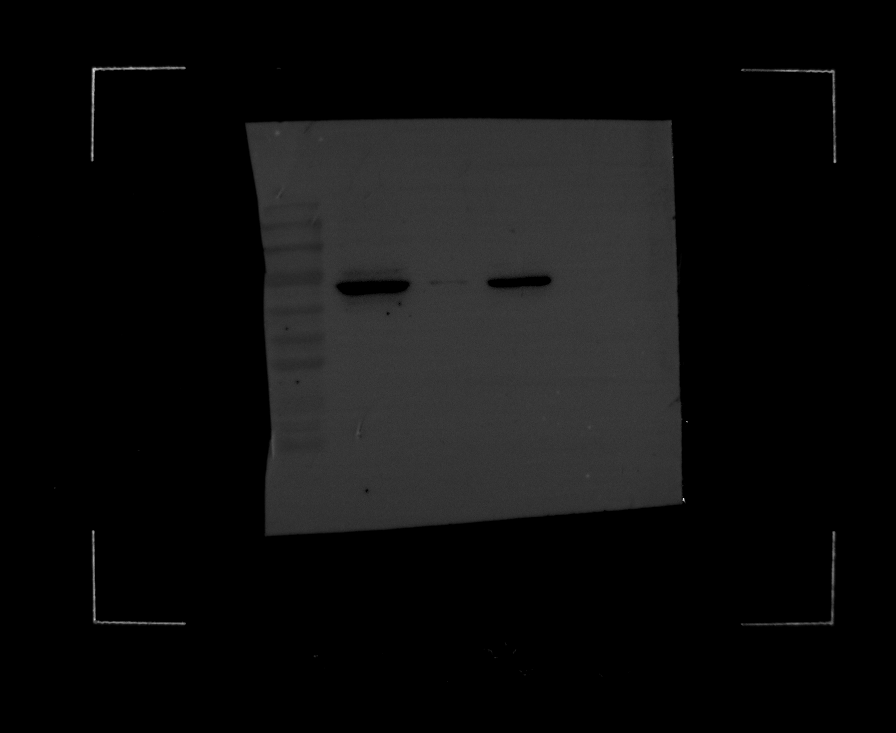

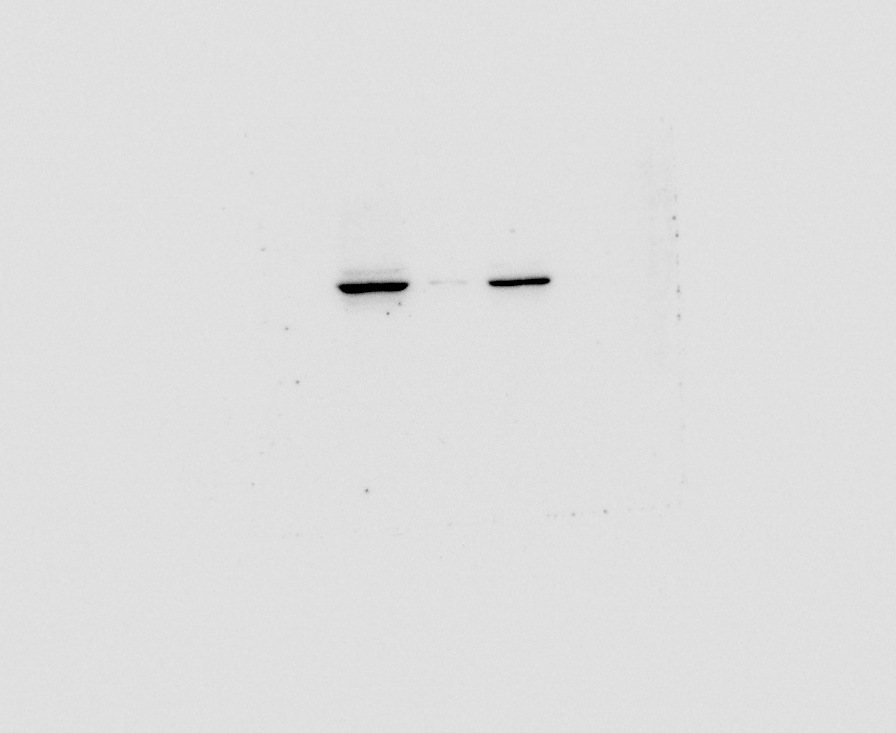


IP-PKM2

USP7


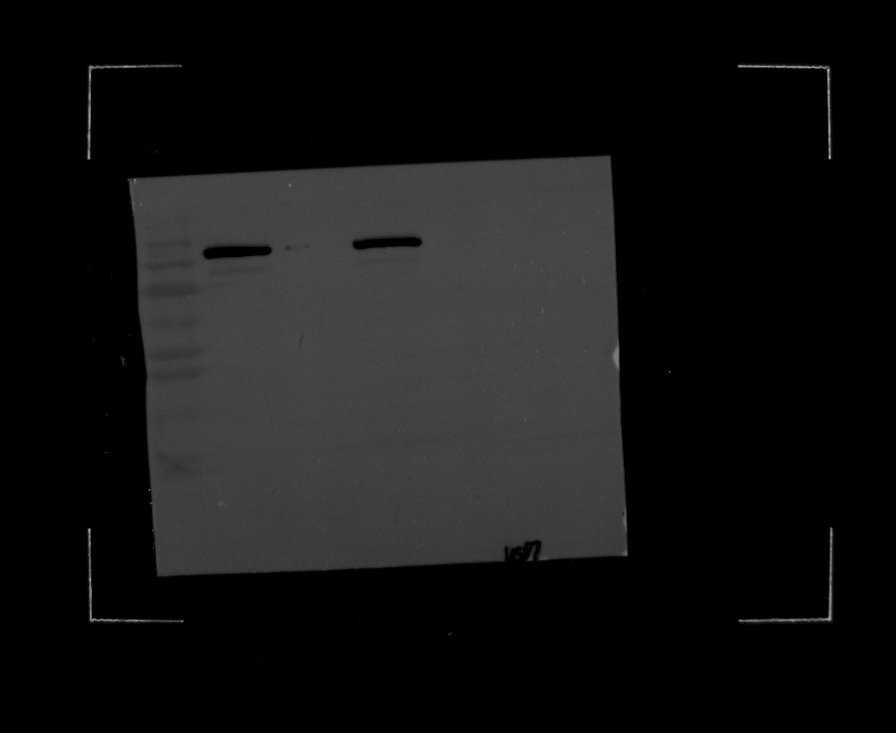

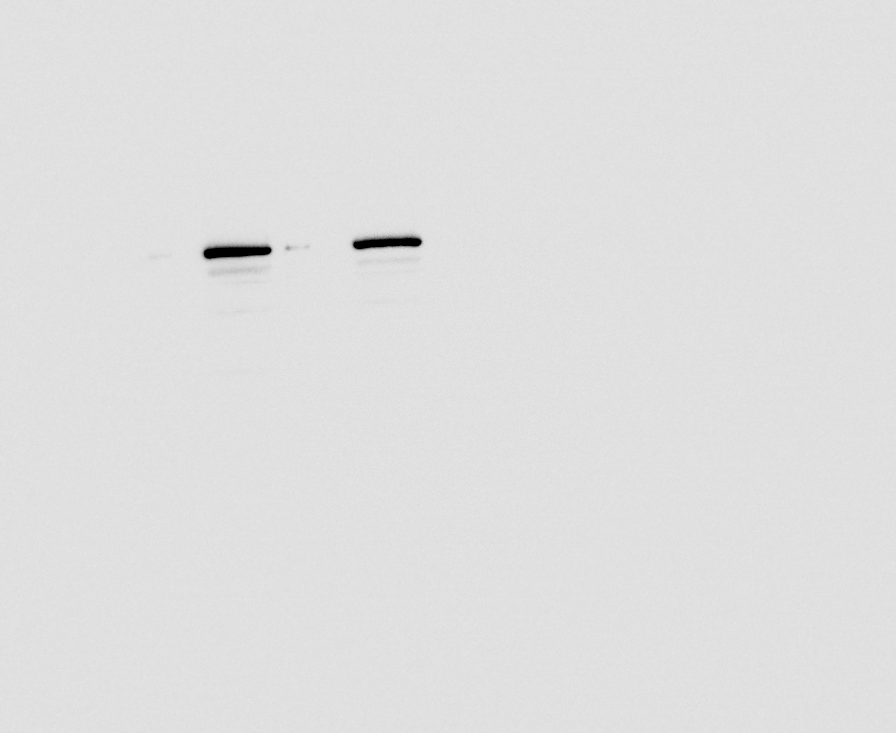


MCL

PKM2


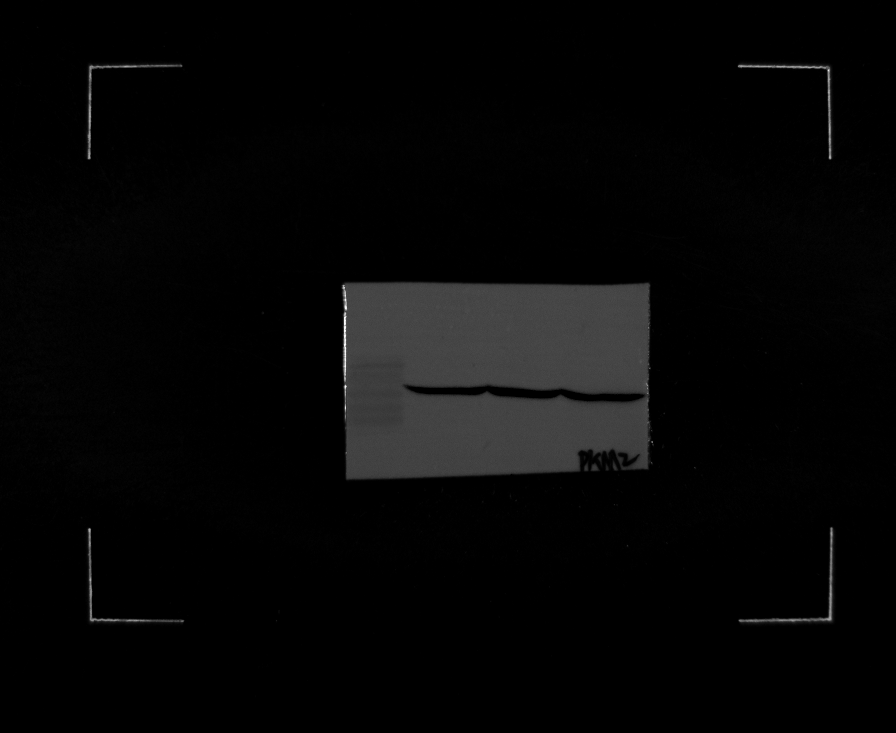

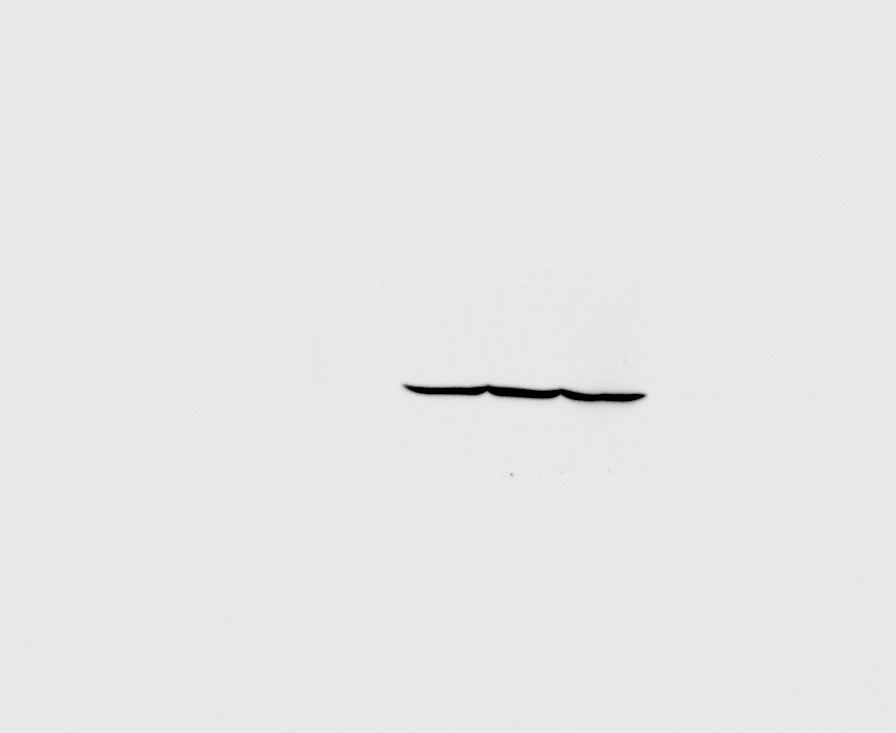


USP7


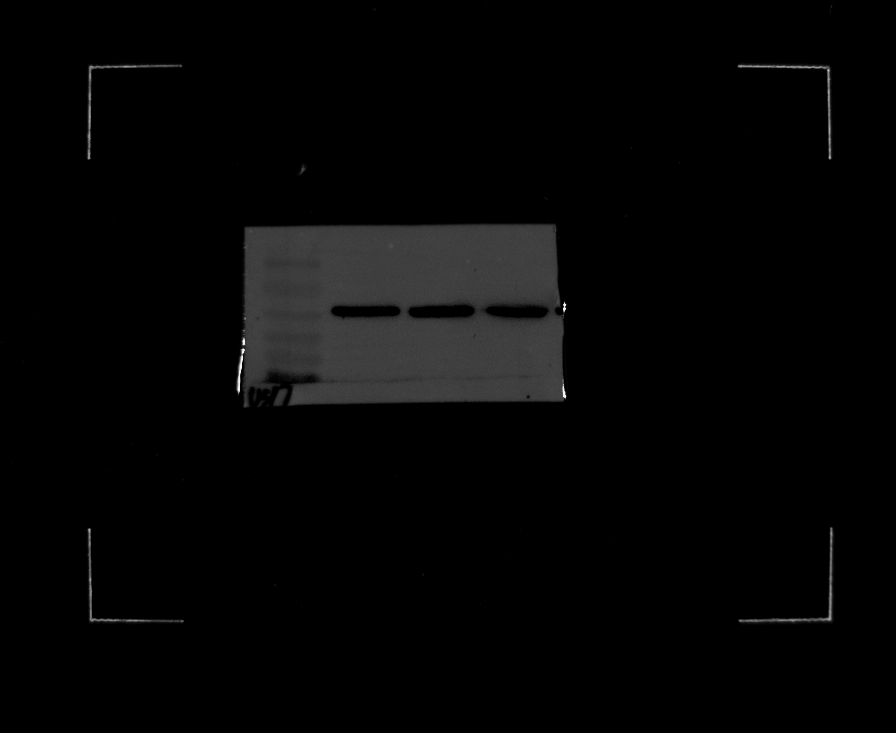

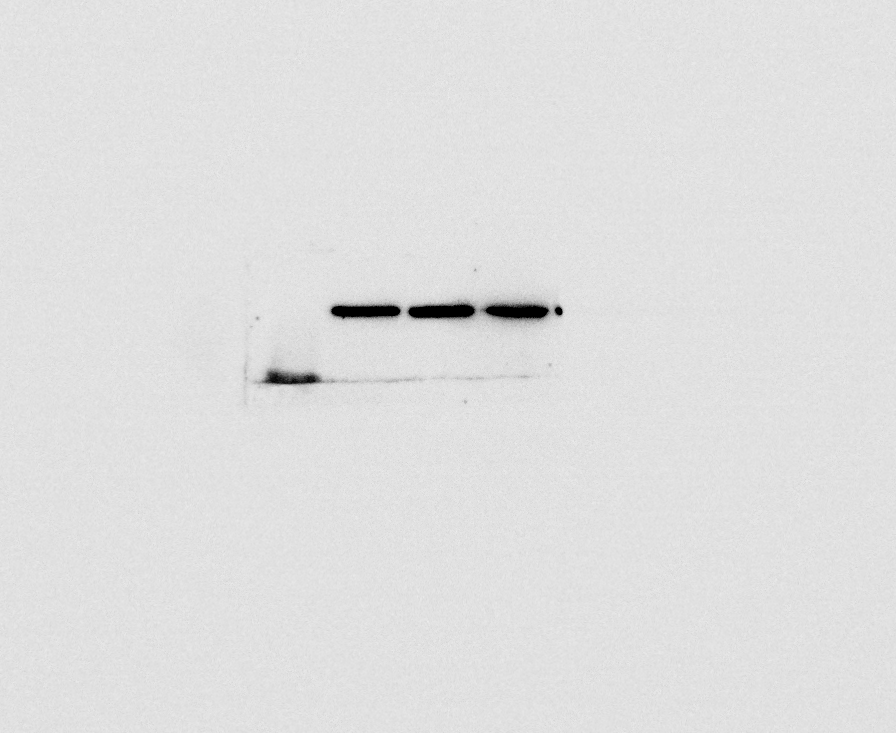


β-actin


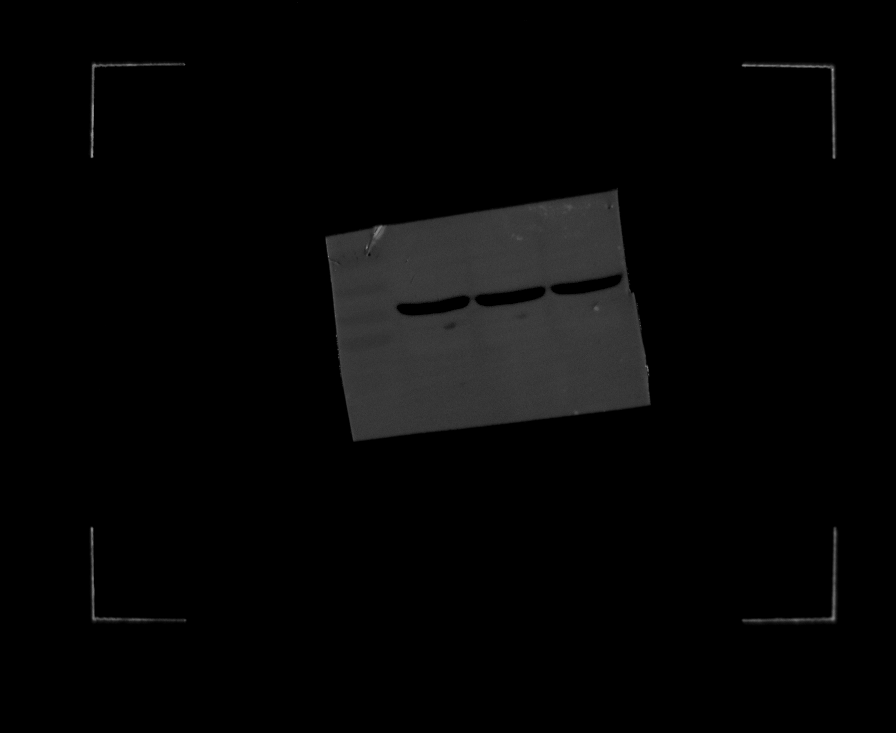

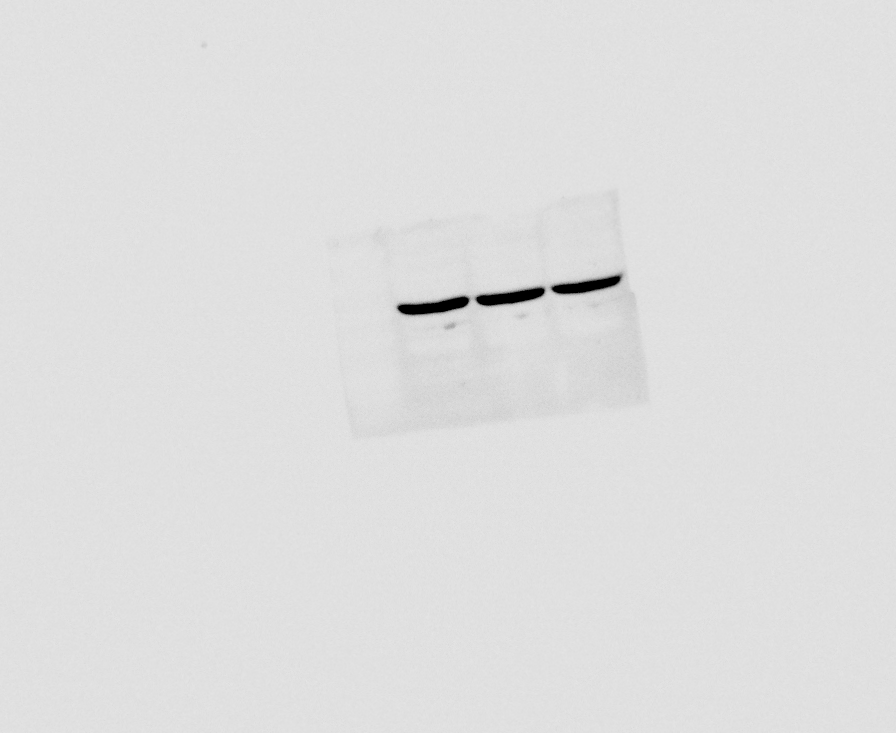


LPS/IFN-γ

IP: USP7

PKM2


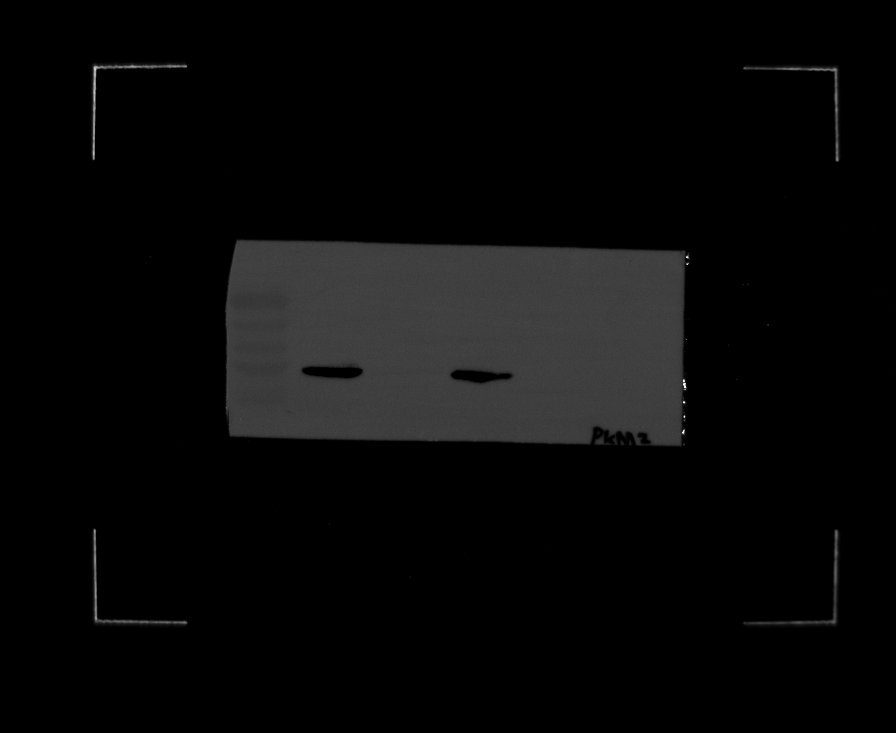

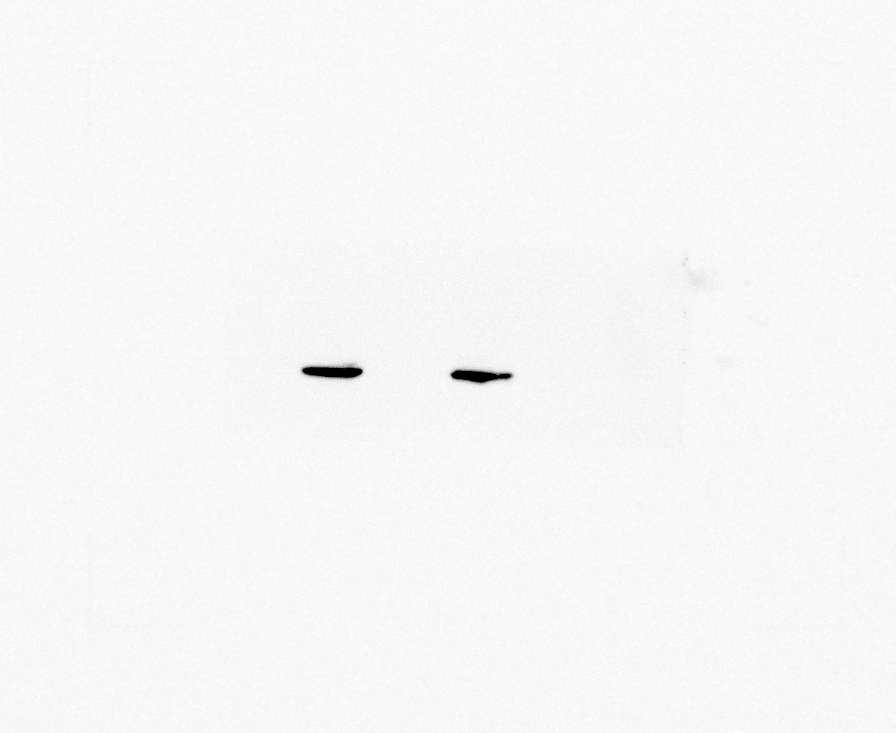


USP7


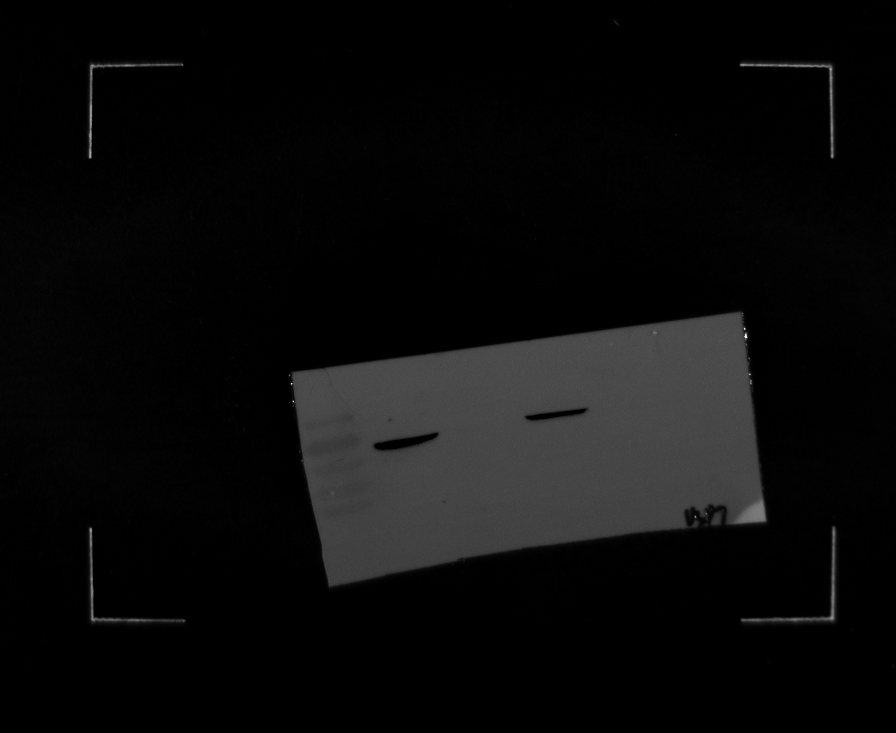

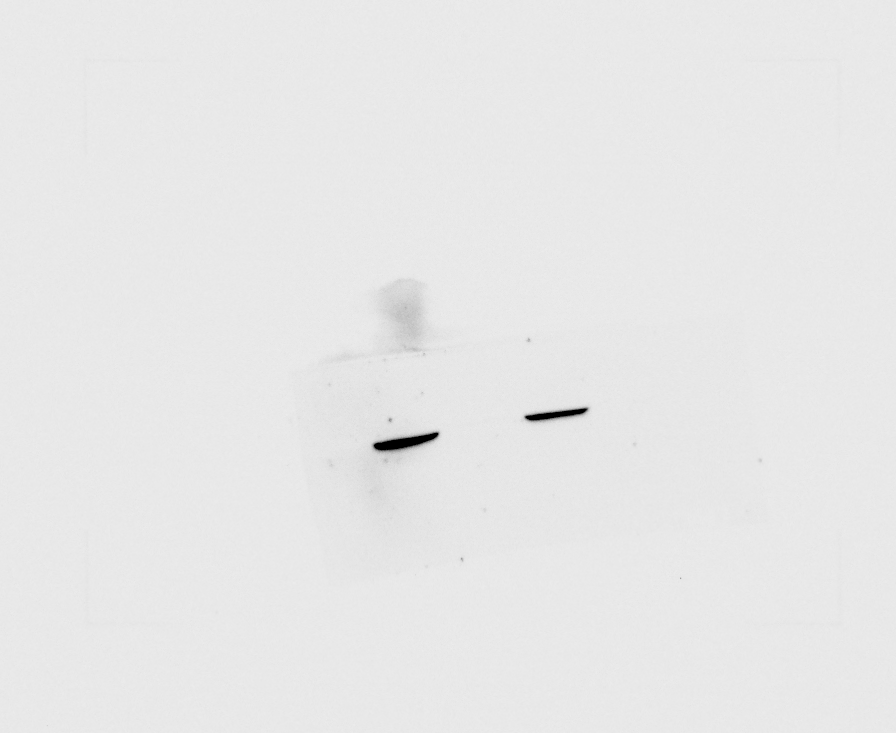


LPS/IFN-γ

IP: PKM2

USP7


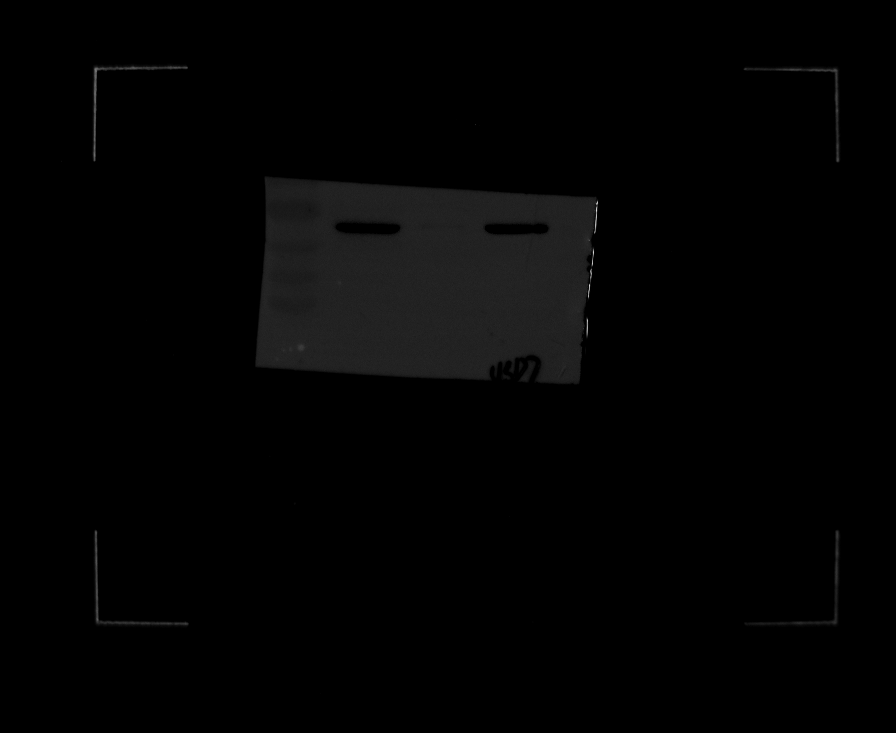

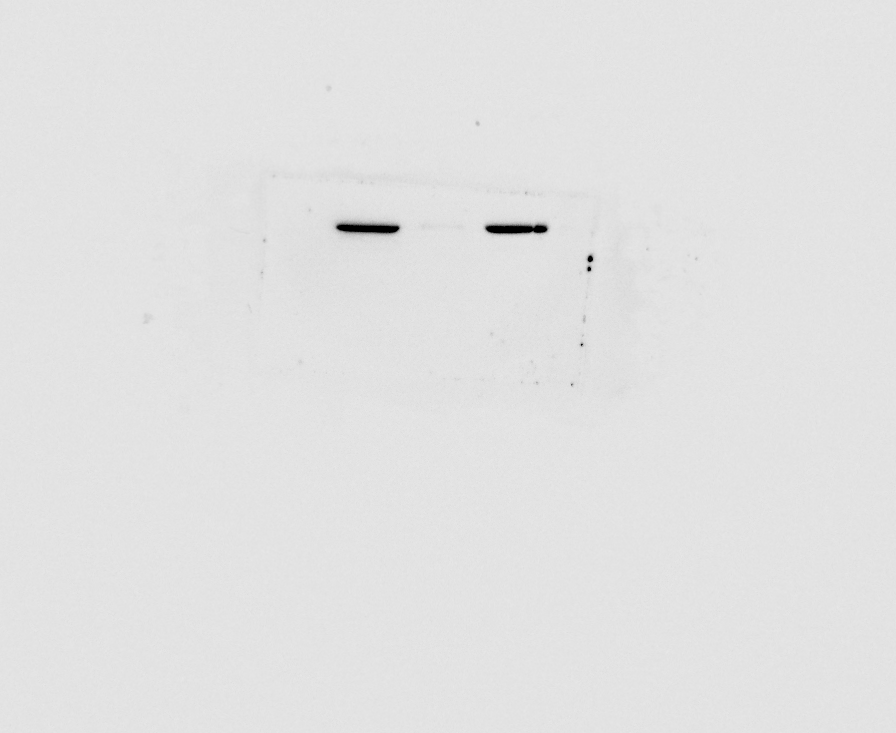


PKM2


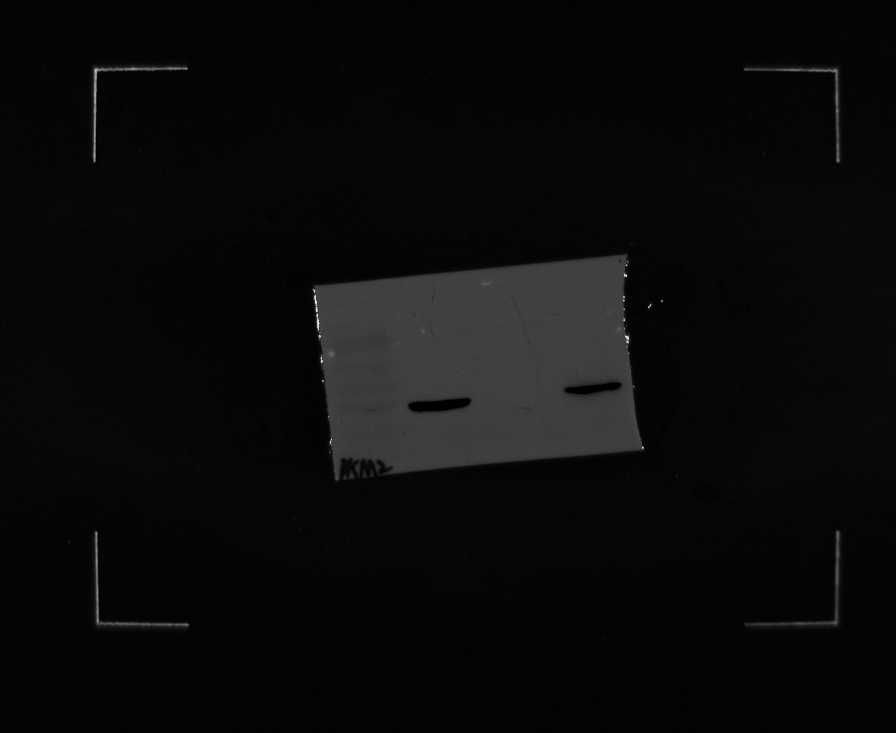

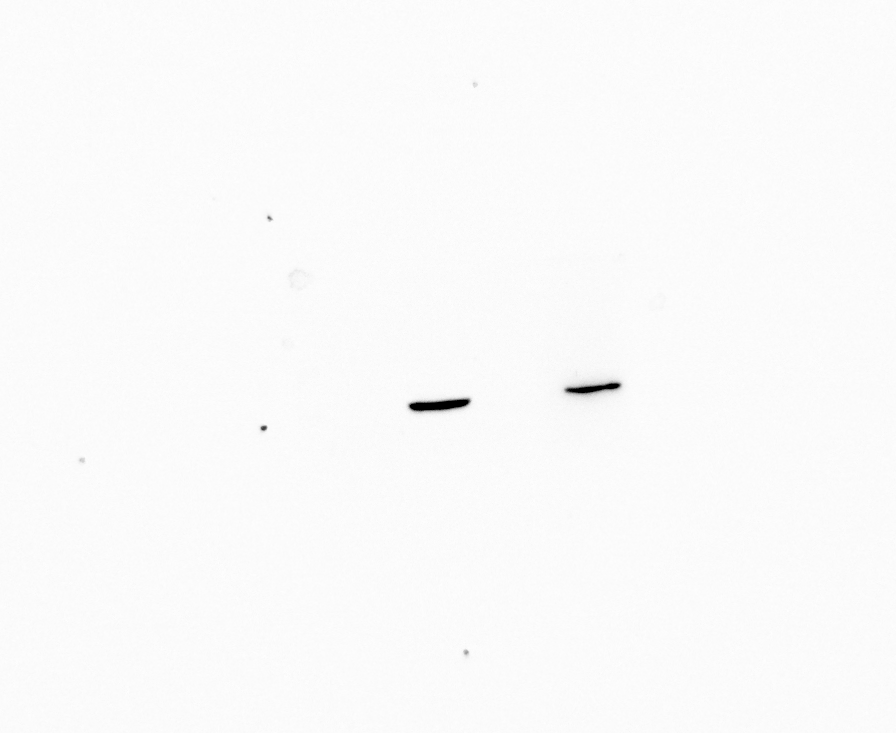


**Figure 6B**

PKM2


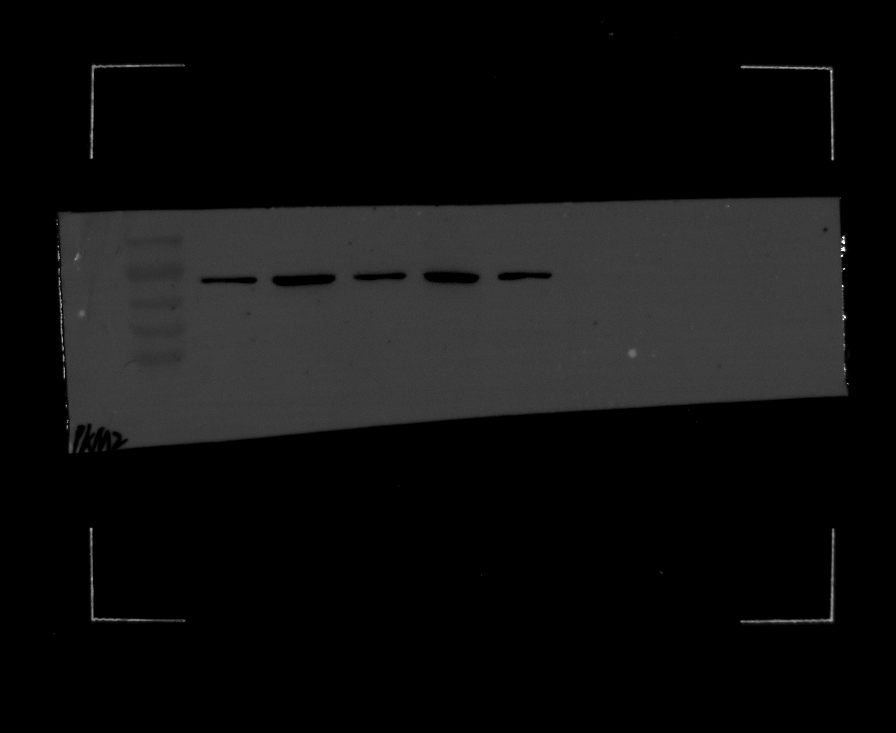

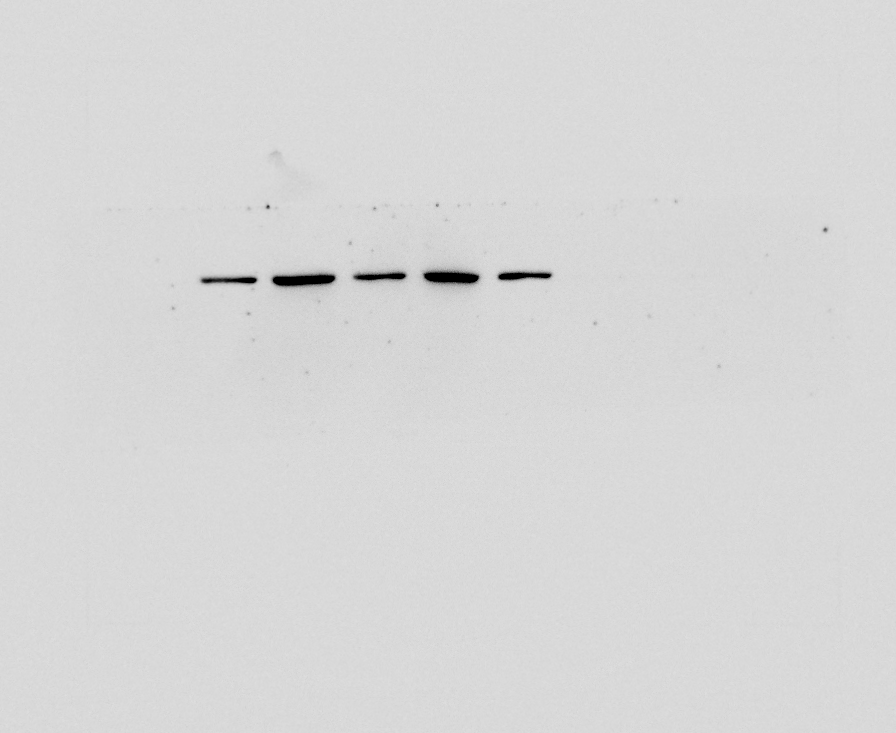


Ub


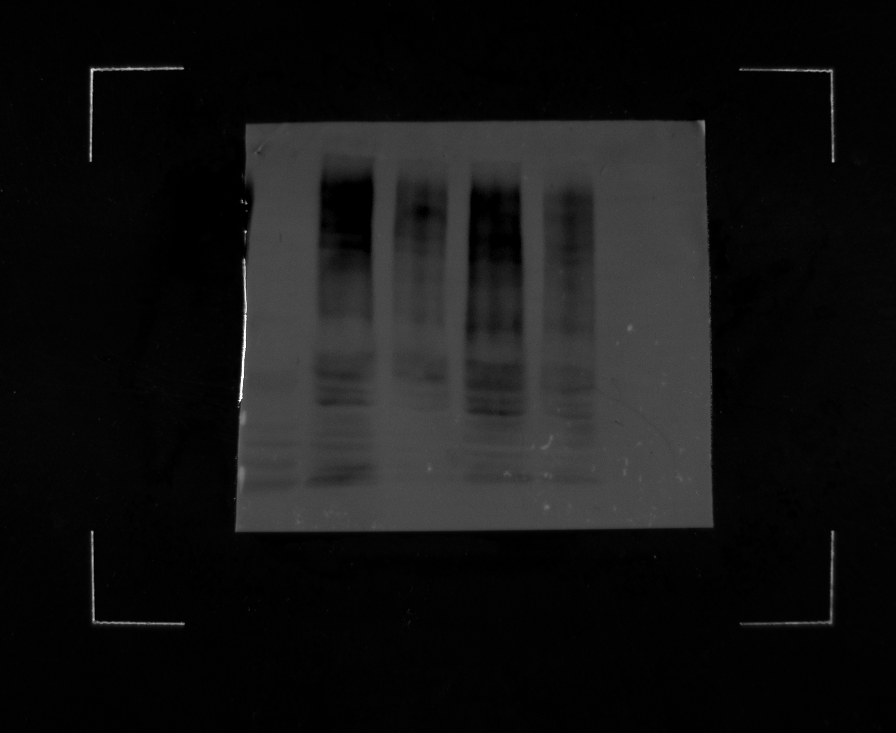

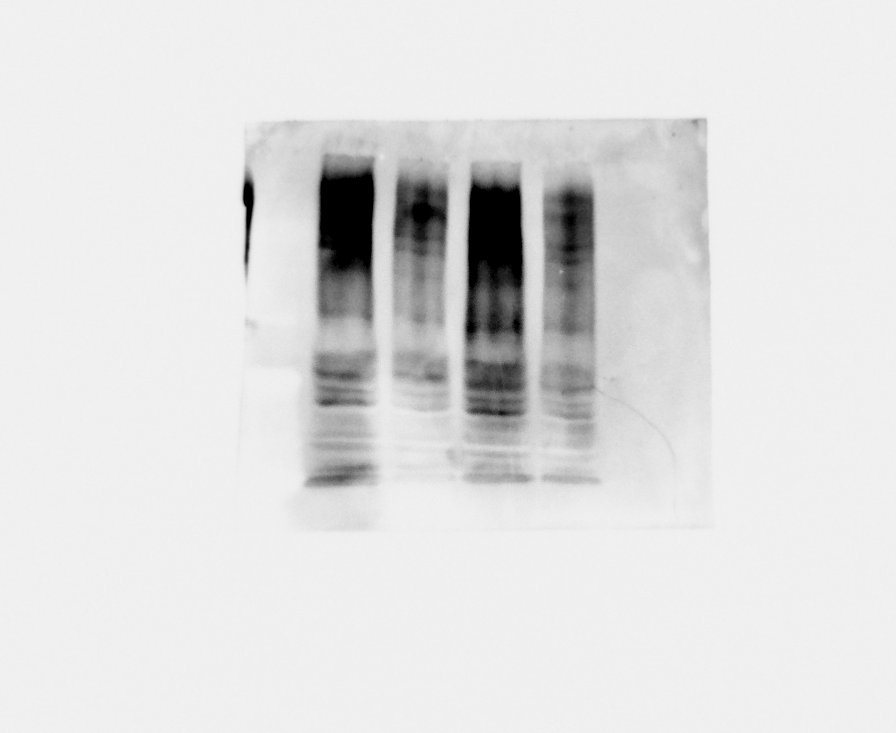


β-actin


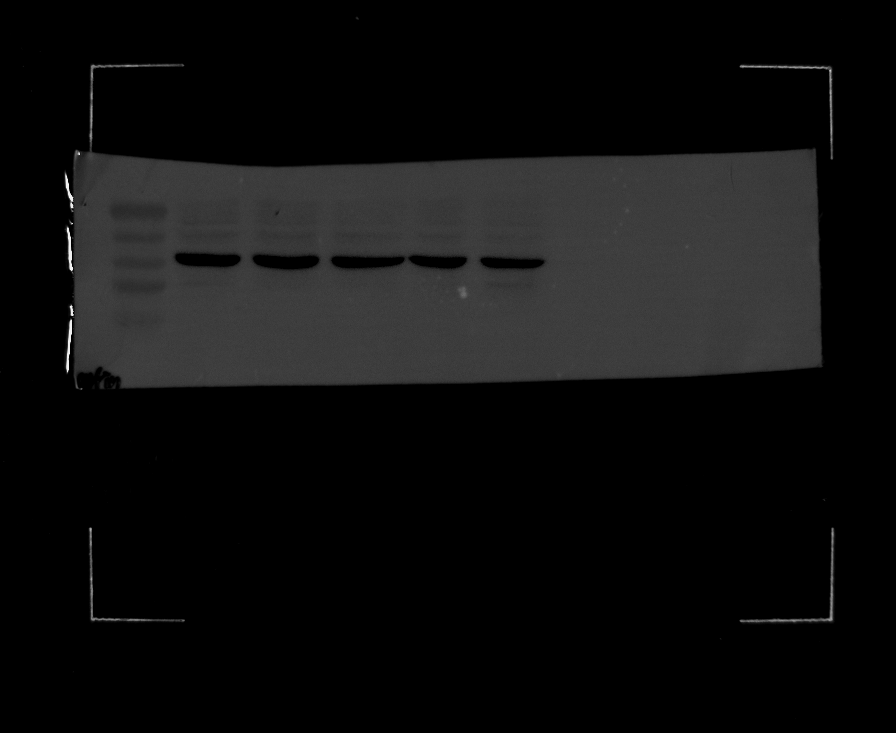

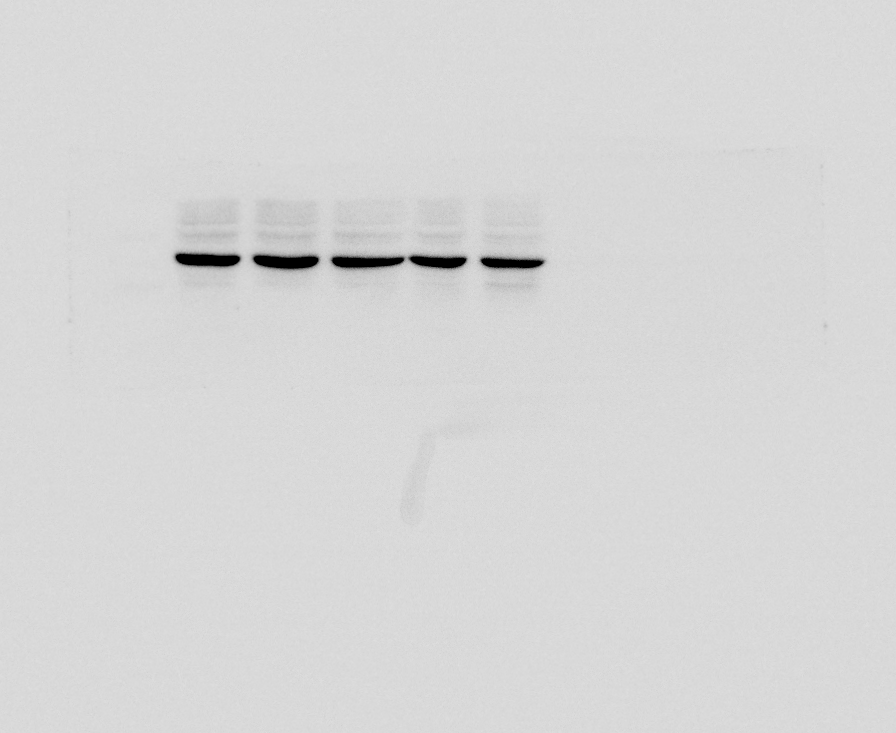


**Figure 6C**

p-pKM2 Y105


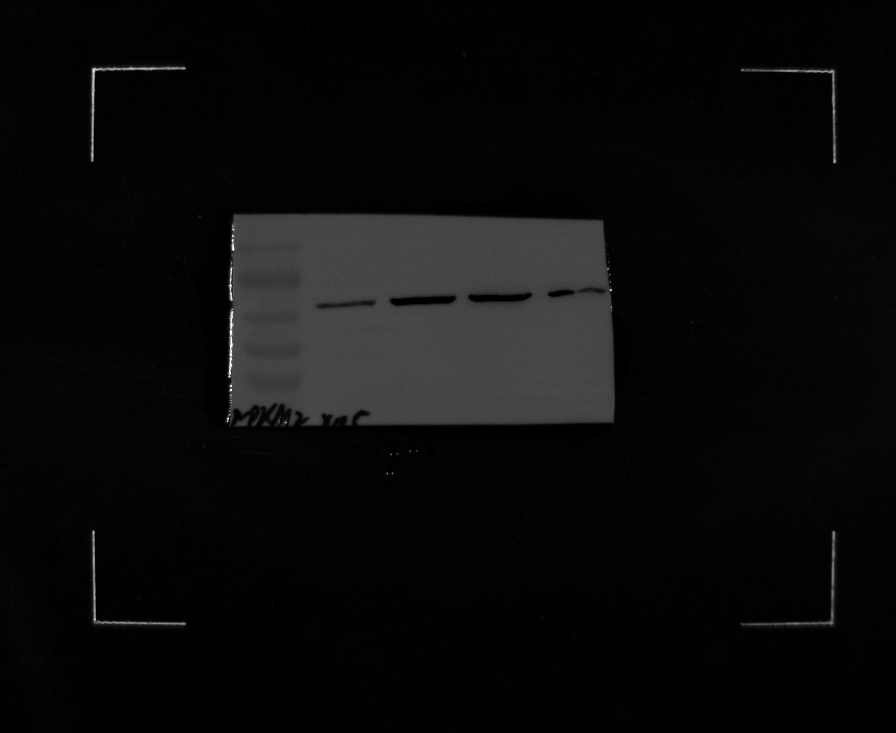

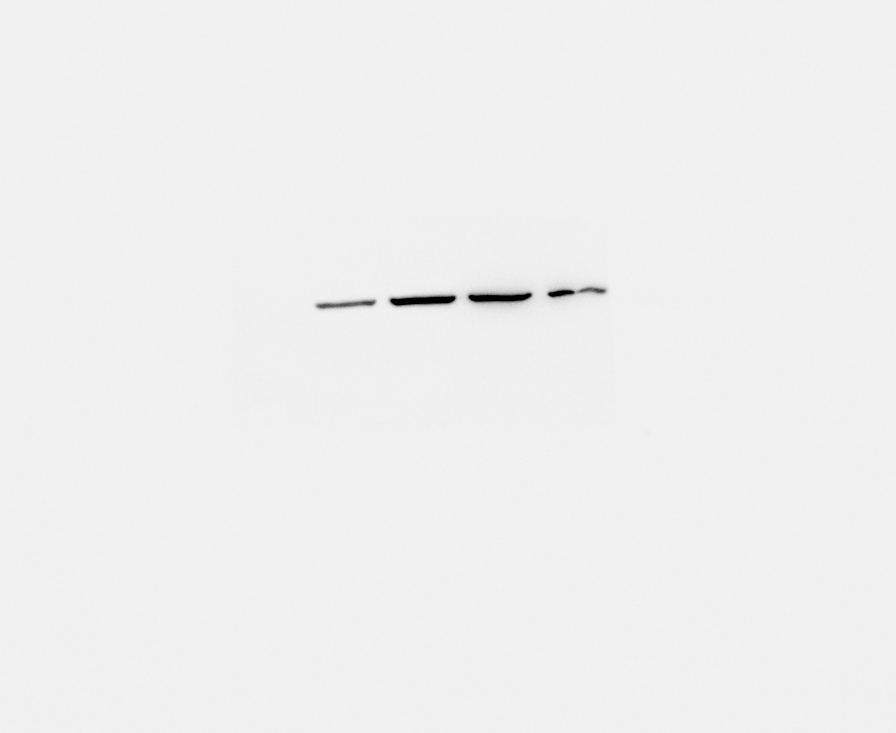


p-pKM2 ser37


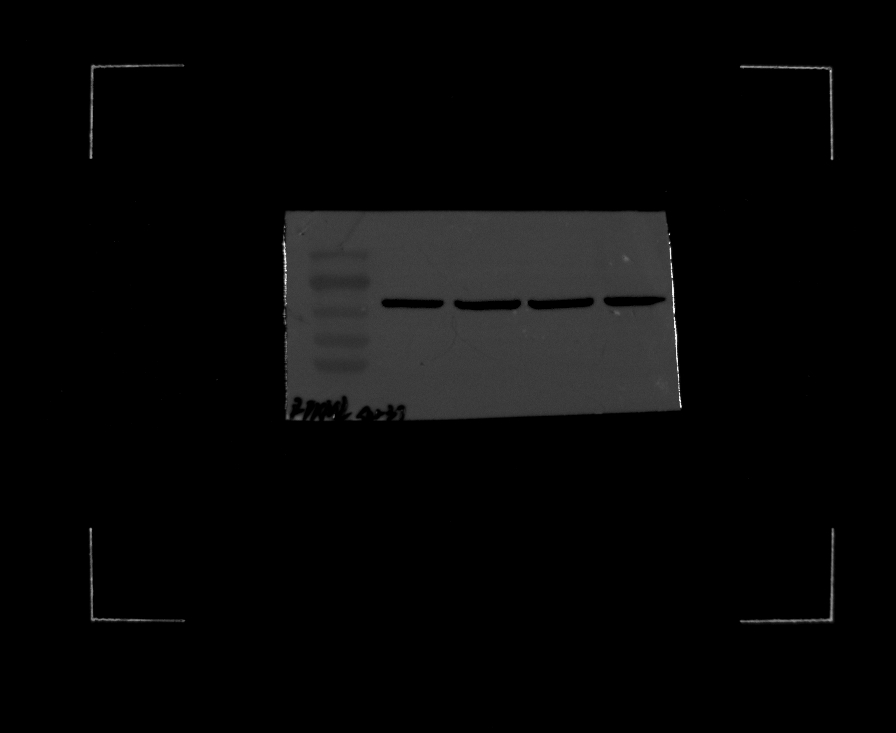

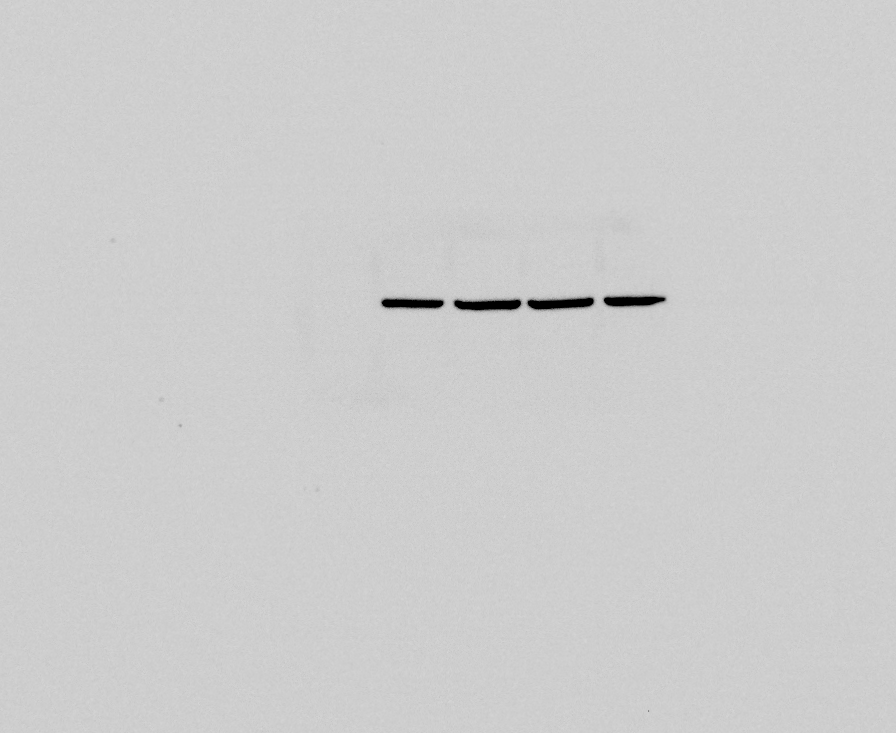


PKM2


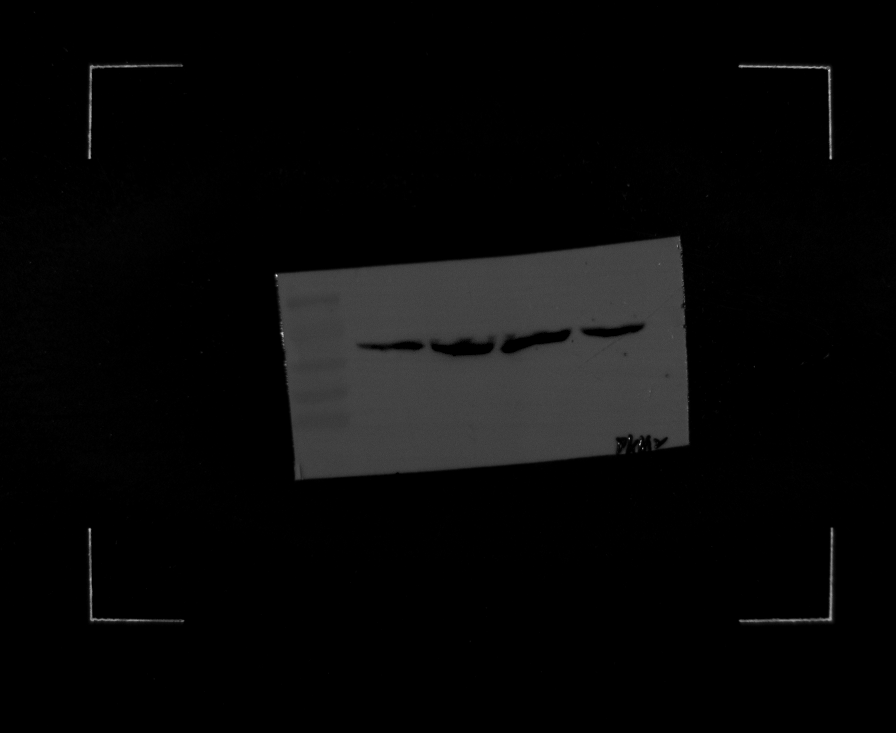

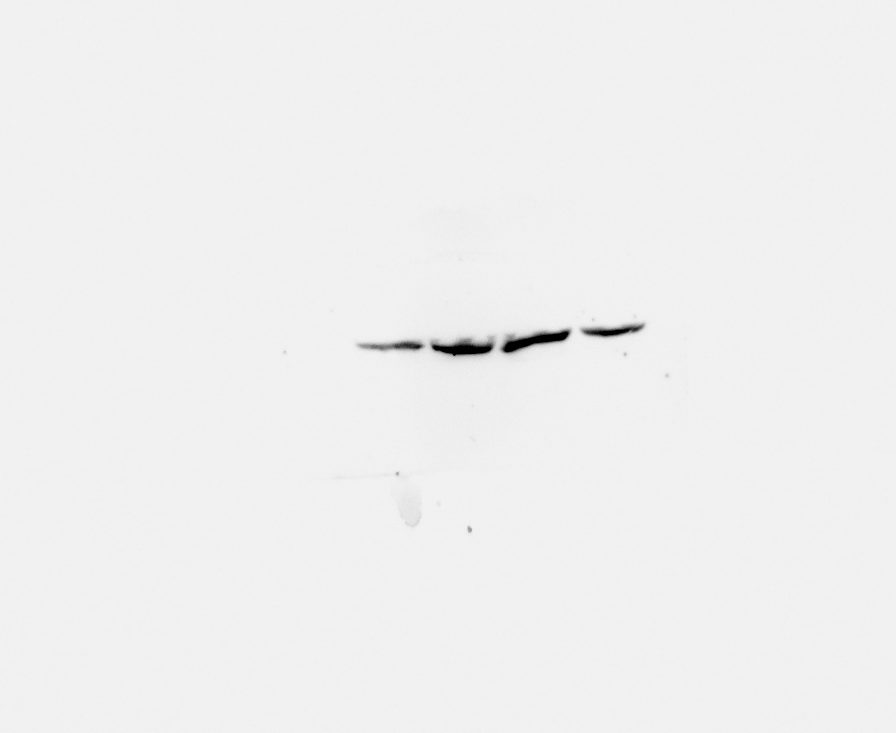


β-actin


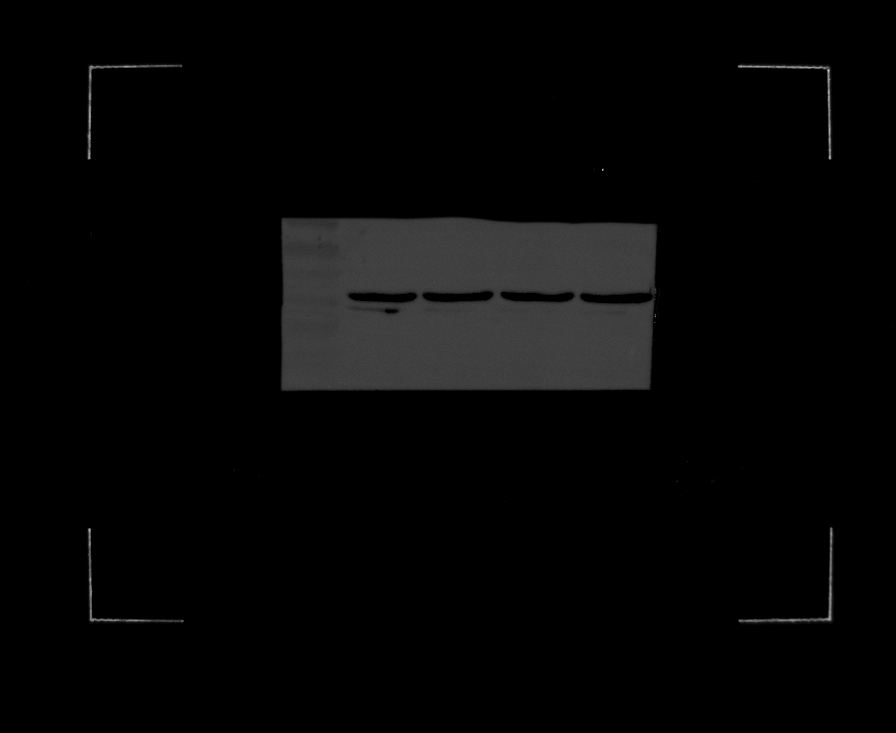

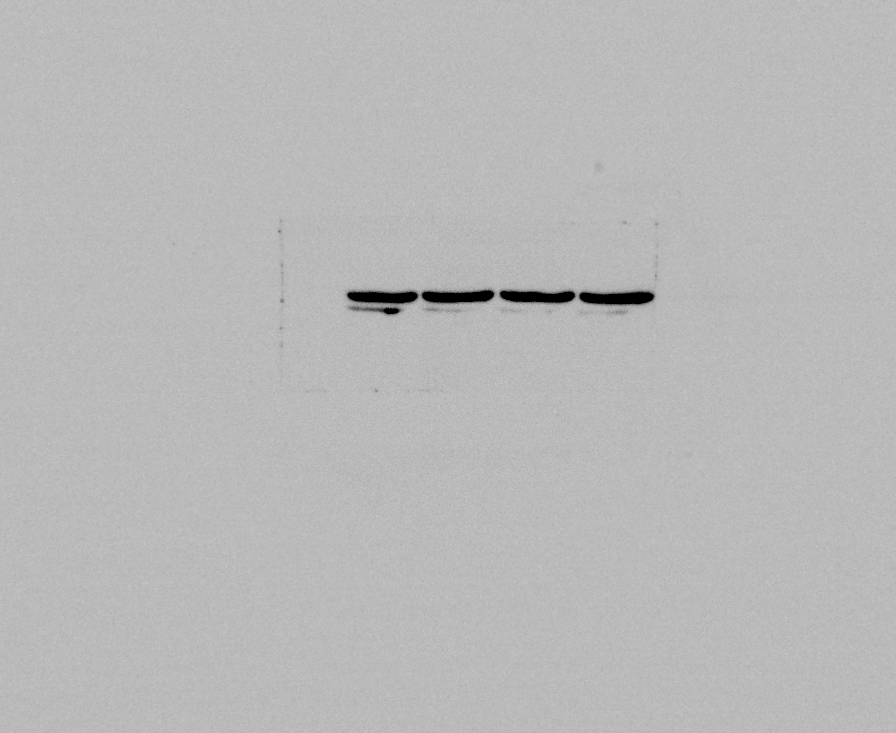


**Figure 6D**

PKM2


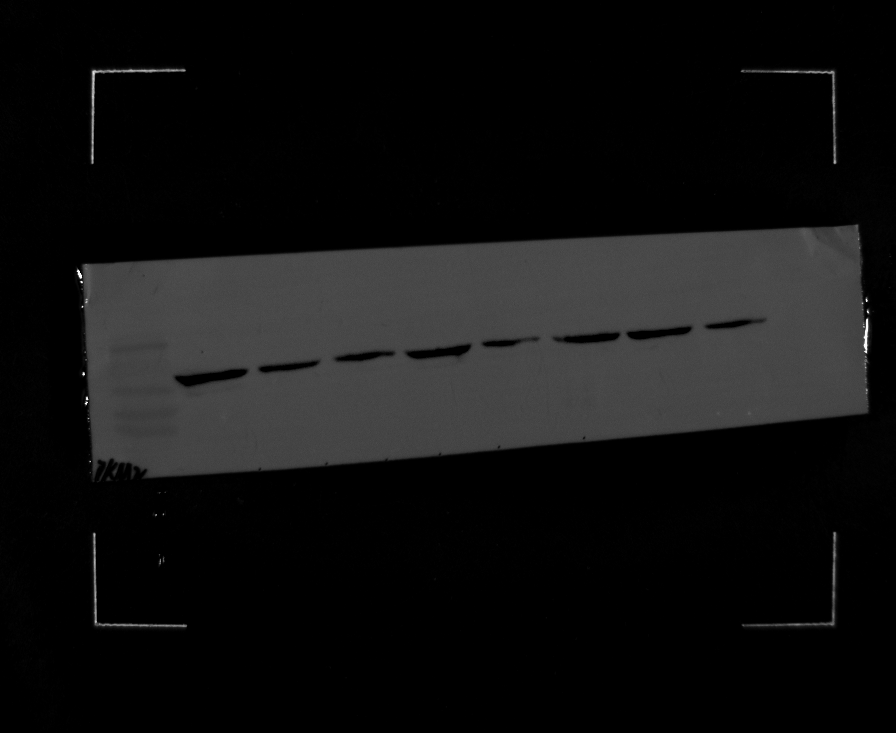

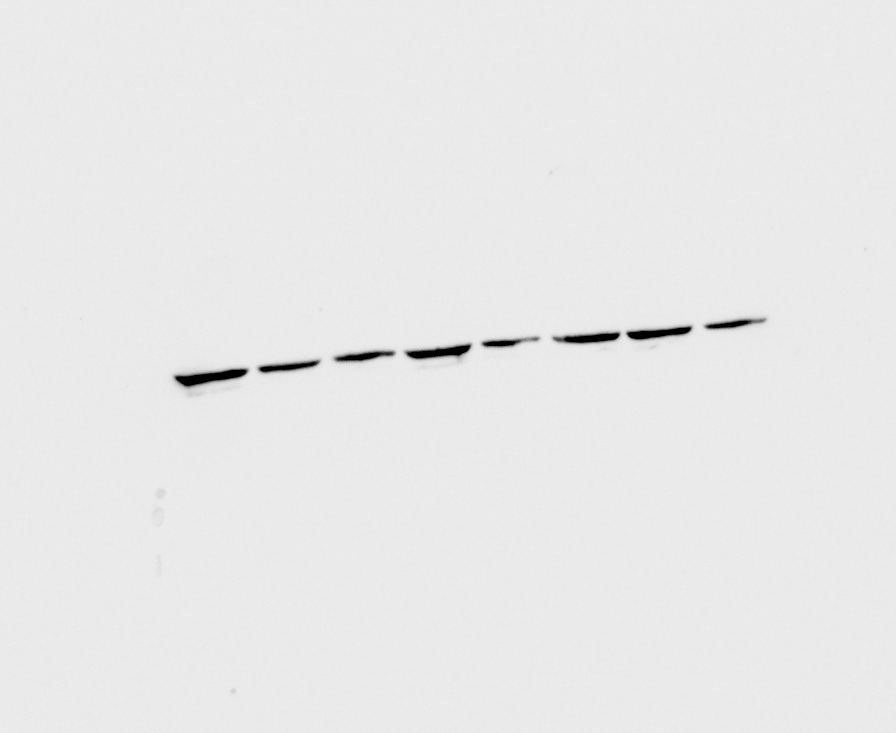


Lamin B


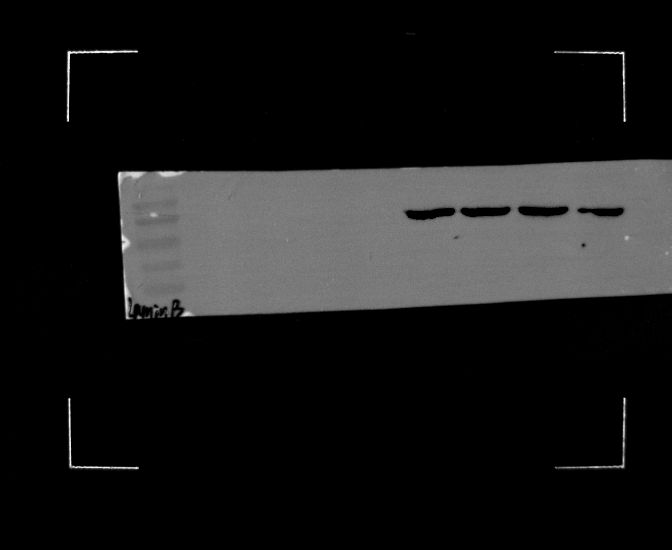

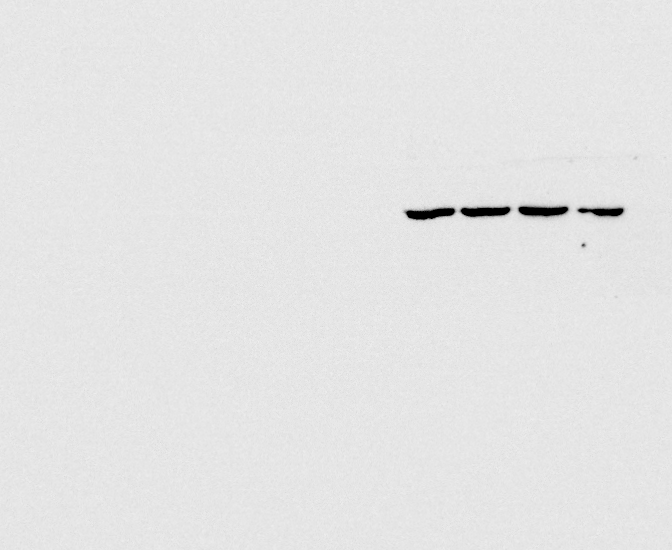


β-actin


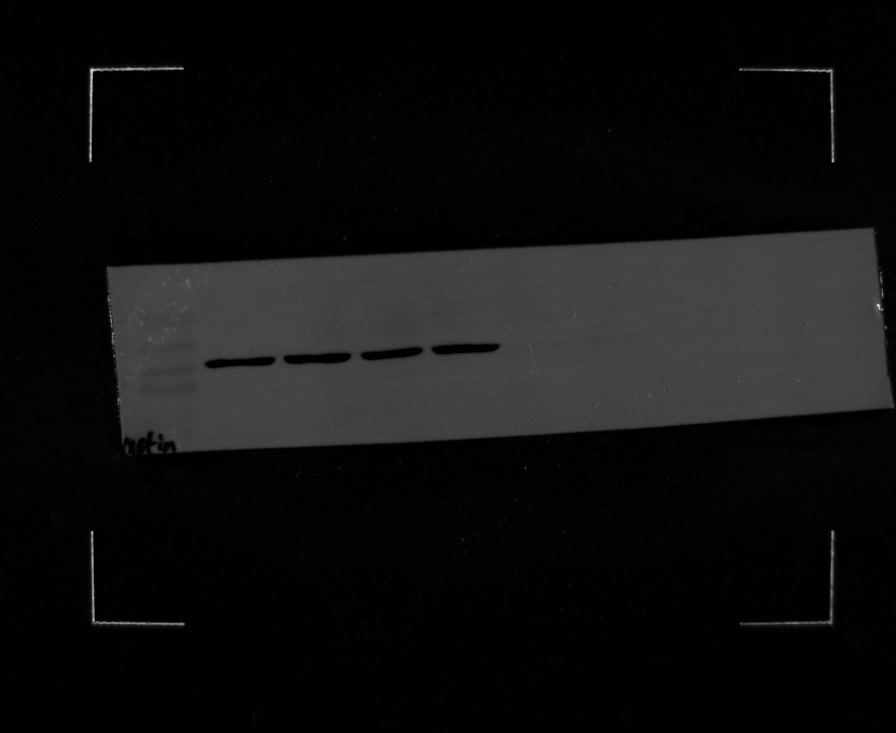

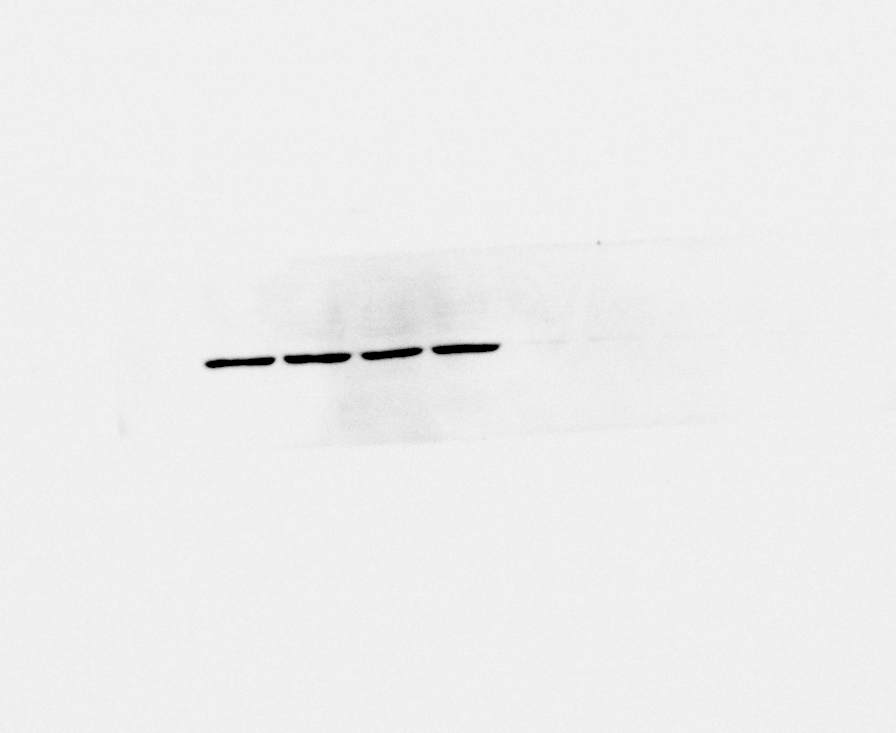


**Figure 8F**

iNOS


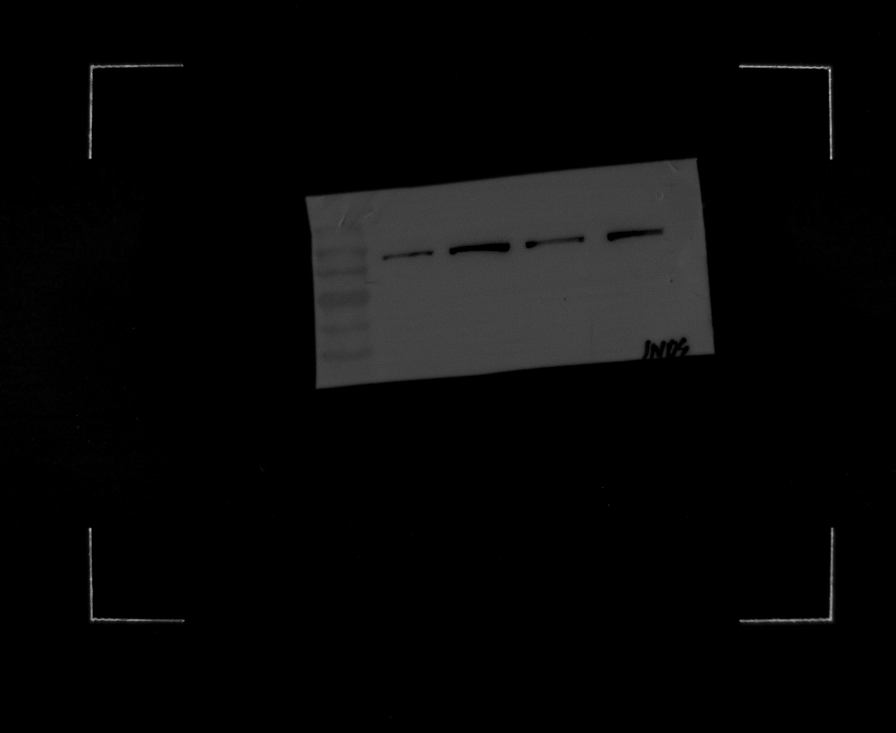

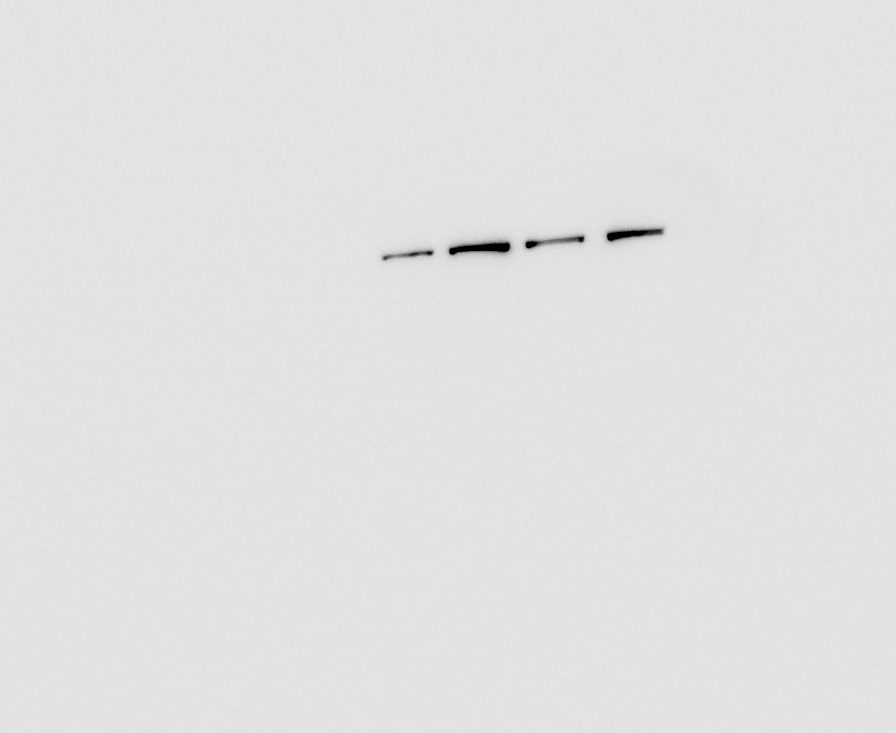


CD86


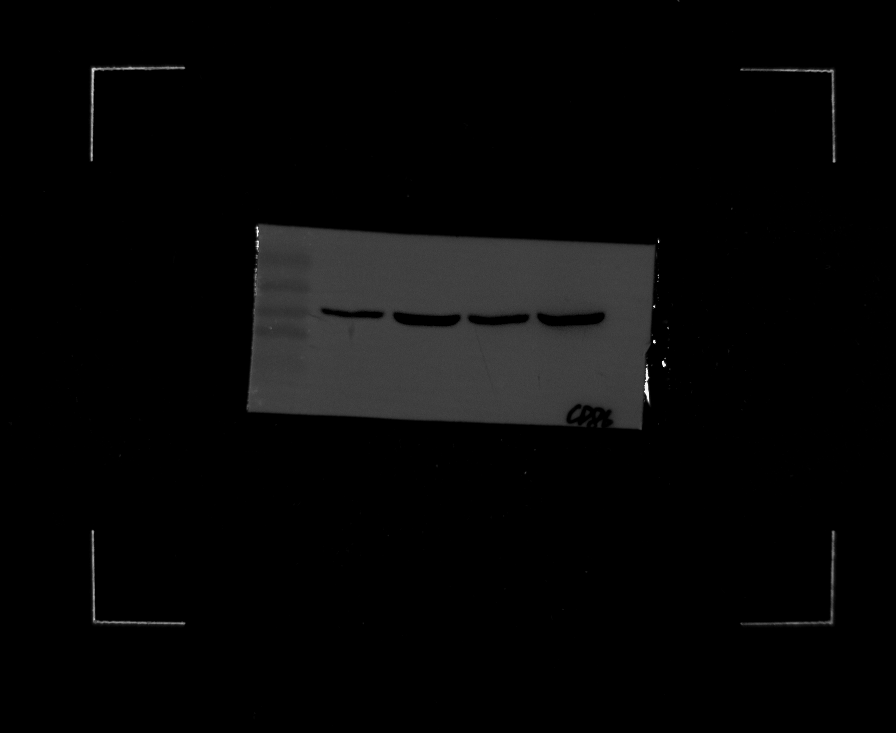

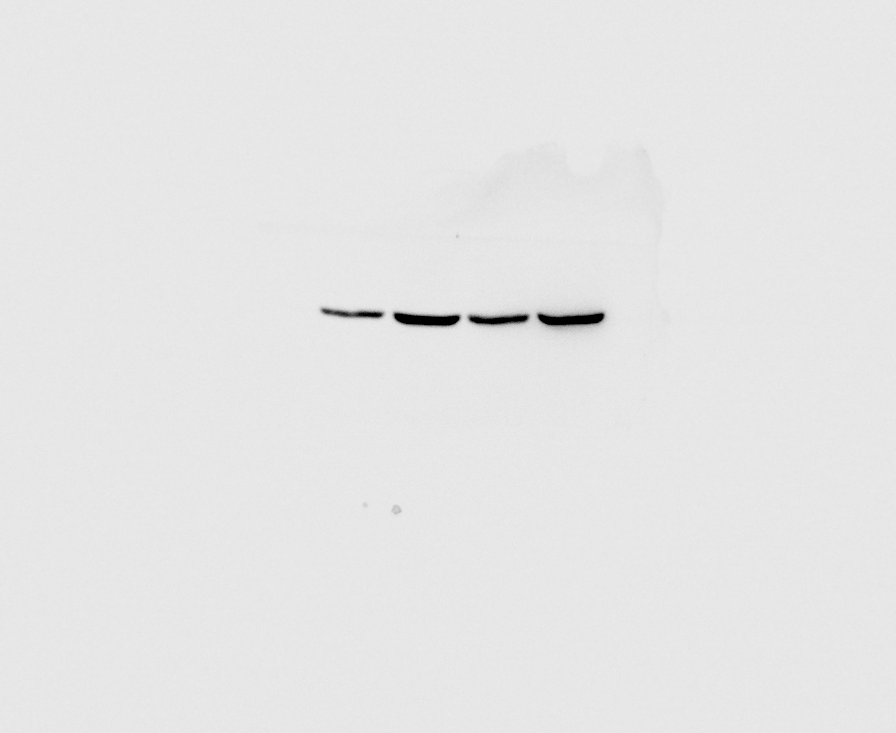


Arg-1

Fizz1

β-actin

**Figure S1G**

iNOS

CD86

Arg-1

β-actin
